# ICOSLG Is Associated with Anti-PD-1 and Concomitant Antihistamine Treatment Response in Advanced Melanoma

**DOI:** 10.3390/ijms252212439

**Published:** 2024-11-19

**Authors:** Domenico Mallardo, Mario Fordellone, Margaret Ottaviano, Giuseppina Marano, Maria Grazia Vitale, Mario Mallardo, Mariagrazia Capasso, Teresa De Cristofaro, Mariaelena Capone, Teresa Meinardi, Miriam Paone, Patrizia Sabatelli, Rosaria De Filippi, Alessandra Cesano, Ernesta Cavalcanti, Corrado Caracò, Sarah Warren, Alfredo Budillon, Ester Simeone, Paolo Antonio Ascierto

**Affiliations:** 1 Melanoma, Cancer Immunotherapy and Development Therapeutics Unit, Istituto Nazionale Tumori IRCCS “Fondazione G. Pascale”, Via Mariano Semmola, 53, 80131 Naples, Italy; margaretottaviano@gmail.com (M.O.); giuseppina.marano@istitutotumori.na.it (G.M.); dott.mariagrazia.vitale@gmail.com (M.G.V.); mario.mallardo39@gmail.com (M.M.); mariagrazia.capasso@istitutotumori.na.it (M.C.); teresa.decristofaro@istitutotumori.na.it (T.D.C.); marilenacapone@gmail.com (M.C.); t.meinardi97@gmail.com (T.M.); miriam.paone@gmail.com (M.P.); p.sabatelli@istitutotumori.na.it (P.S.); ester.simeone@gmail.com (E.S.); paolo.ascierto@gmail.com (P.A.A.); 2 Medical Statistics Unit, Universitiy of Campania “Luigi Vanvitelli”, 81100 Naples, Italy; mario.fordellone@unicampania.it; 3 Department of Clinical Medicine and Surgery, University of Naples Federico II, 80138 Naples, Italy; rdefilip@unina.it; 4 ESSA Pharma, South San Francisco, CA 94080, USA; acesano@essapharma.com (A.C.); swarren@nanostring.com (S.W.); 5 Division of Laboratory Medicine, Istituto Nazionale Tumori IRCCS “Fondazione G. Pascale”, 80131 Naples, Italy; e.cavalcanti@istitutotumori.na.it; 6 Division of Surgery of Melanoma and Skin Cancer, Istituto Nazionale Tumori IRCCS “Fondazione G. Pascale”, 80131 Naples, Italy; corrado.caraco@istitutotumori.na.it; 7 Istituto Nazionale Tumori IRCCS “Fondazione G. Pascale”, 80131 Naples, Italy; a.budillon@istitutotumori.na.it

**Keywords:** cutaneous melanoma, checkpoint inhibition, cetirizine, biomarker

## Abstract

We previously demonstrated that patients with metastatic unresectable stage IIIb–IV melanoma receiving cetirizine (a second-generation H1 antagonist antihistamine) premedication with immunotherapy had better outcomes than those not receiving cetirizine. In this retrospective study, we searched for a gene signature potentially predictive of the response to the addition of cetirizine to checkpoint inhibition (nivolumab or pembrolizumab with or without previous ipilimumab). Transcriptomic analysis showed that inducible T cell costimulator ligand (ICOSLG) expression directly correlated with the disease control rate (DCR) when detected with a loading value > 0.3. A multivariable logistic regression model showed a positive association between the DCR and ICOSLG expression for progression-free survival and overall survival. ICOSLG expression was associated with CD64, a specific marker of M1 macrophages, at baseline in the patient samples who received cetirizine concomitantly with checkpoint inhibitors, but this association was not present in subjects who had not received cetirizine. In conclusion, our results show that the clinical advantage of concomitant treatment with cetirizine during checkpoint inhibition in patients with malignant melanoma is associated with high ICOSLG expression, which could predict the response to immune checkpoint inhibitor blockade.

## 1. Introduction

Checkpoint inhibition has become a mainstay of treatment for advanced cutaneous melanoma, providing improved survival compared with previous therapies [1,2]. However, a proportion of patients still experience poor outcomes after immunotherapy, as confirmed by several studies [3,4,5,6,7]. As a result, identifying predictive biomarkers of the response to immunotherapy is an important area of research, aiming to prevent overtreatment, unnecessary risk of toxicity, and wasteful resource usage. Since gene expression signatures in liquid biopsies are non-invasive repeatable tests that may provide high specificity and sensitivity [8], they are widely investigated as potential predictive or prognostic markers in cancer patients, specifically in subjects with cutaneous melanoma [9,10,11].

Patients with unresectable cutaneous melanoma were observed to have improved overall survival (OS) and progression-free survival (PFS) when premedicated with cetirizine (a second-generation H1 antagonist antihistamine) at the beginning of checkpoint-inhibiting immunotherapy compared with non-premedicated subjects [12]. Nonetheless, some patients did not show clinical benefits from adding cetirizine [12]. The effect of cetirizine administration was associated with a shift in circulating macrophages from the M2 to the M1 phenotype. This observation suggests that the antihistamine may promote the antitumor activity of macrophages through an interferon-gamma pathway that drives macrophages from the immunosuppressive phenotype to the M1 phenotype, which has antitumoral activities based on phagocytosis and antigen presentation [13]. Building on these results and taking advantage of our experience with gene expression profiling techniques [14,15], we aimed to identify a gene expression signature linked to the immune state of patients and associated with the clinical response to the addition of cetirizine to checkpoint inhibition.

In a retrospective study, we compared clinical outcomes and blood sample transcriptomic analysis in patients with unresectable cutaneous malignant melanoma treated with checkpoint inhibitors (the anti-PD1 nivolumab or pembrolizumab, with or without previous anti-CTLA4 ipilimumab), either with or without cetirizine premedication.

## 2. Results

### 2.1. Patient Characteristics and Outcomes

Blood and tissue samples were collected between April 2016 and June 2018. Patient characteristics at baseline are reported in Table 1. Overall, 116 patients were included in the study; 51 (44.0%) were females, 22 (20.4%) had a *BRAF* mutation, 86 (79.6%) had a wild-type *BRAF* (BRAF state was missing for eight patients), and 84 (75.7%) had brain metastases. Sixty-seven (57.7%) patients had received cetirizine before immunotherapy and 49 (42.3%) had not. These two groups of patients had similar characteristics. However, those treated with cetirizine had a higher frequency of mutated *BRAF* and central nervous system (CNS) metastases, along with more elevated levels of LDH. Table 1 reports anti-PD-1 agents used in the included patients. Nivolumab was administered at a dose of 3 mg/kg every 2 weeks and pembrolizumab at a dose of 2 mg/kg mg every 3 weeks. Ipilimumab was administrated at a dose of 3 mg/kg IV every 3 weeks for a maximum of 4 doses.

At the first disease response assessment (3 months), patients treated with cetirizine had significantly better response to therapy; the DCR was recorded in 55 (DCR: 47.4%) patients. Respondents were 38/67 (DCR: 56.7%) among the patients who had received cetirizine and 17/49 (DCR: 34.7%) among those who had not. The objective response rate (ORR) for the whole cohort was 29.3% of patients but was higher in the cetirizine group than in the group without cetirizine (ORR: 22.4%).

### 2.2. Gene Expression Signatures

**Figure 1:**
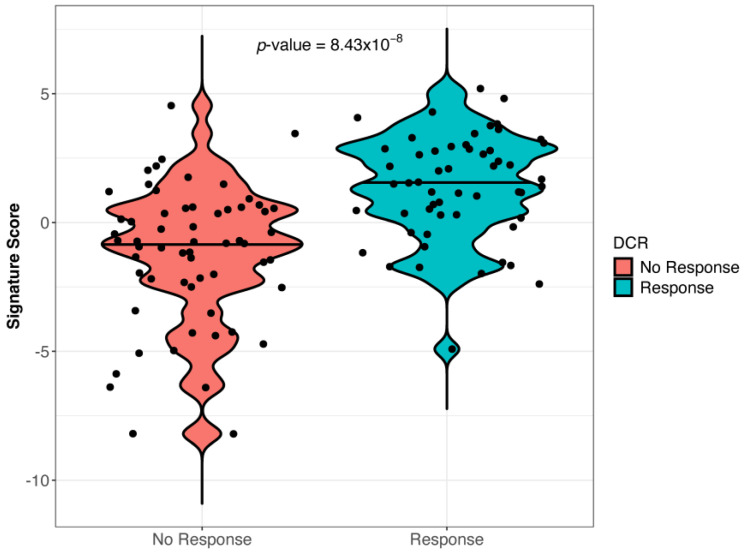
Gene signature expression in responder and non-responder patients (Wilcoxon test, *p* = 8.3 × 10^−8^). The signature included 15 genes, namely *TRAF1*, *S100A9*, *S100A8*, *S100A12*, *OLR1*, *NF1*, *MMP9*, *LDHA*, *ITGAM*, *ICOSLG*, *HLA-DPA1*, *FSTL3*, *CST2*, *CLEC5A*, and *ANLN*.

The analysis of blood samples collected at baseline showed that a gene expression signature was associated (the cut-off point was 1.76 at the 26th percentile) with response to anti-PD-1 and cetirizine treatment. It included 15 genes (*TRAF1*, *S100A9*, *S100A8*, *S100A12*, *OLR1*, *NF1*, *MMP9*, *LDHA*, *ITGAM*, *ICOSLG*, *HLA-DPA1*, *FSTL3*, *CST2*, *CLEC5A*, and *ANLN*). Only the increased expression of inducible T cell costimulator ligand (ICOSLG) with a loading value > 0.3 directly correlated with a higher probability of response to treatment (Supplementary Figure S1A). The other genes of the signature were negatively correlated with the probability of response. The accuracy of the association between signature expression and the DCR is shown in Supplementary Figure S1B. The expression of the signature was significantly more frequent in responders than in non-responders (*p* < 0.01) (Figure 1).

To investigate the effect of high versus low expression of the ICOSLG signature on oncological outcomes, the best cut-off point of signature expression was determined by the Simon permutation approach, and a low-expression group with 30 patients and a high-expression group with 86 patients were identified (Supplementary Figure S2).

**Figure 2:**
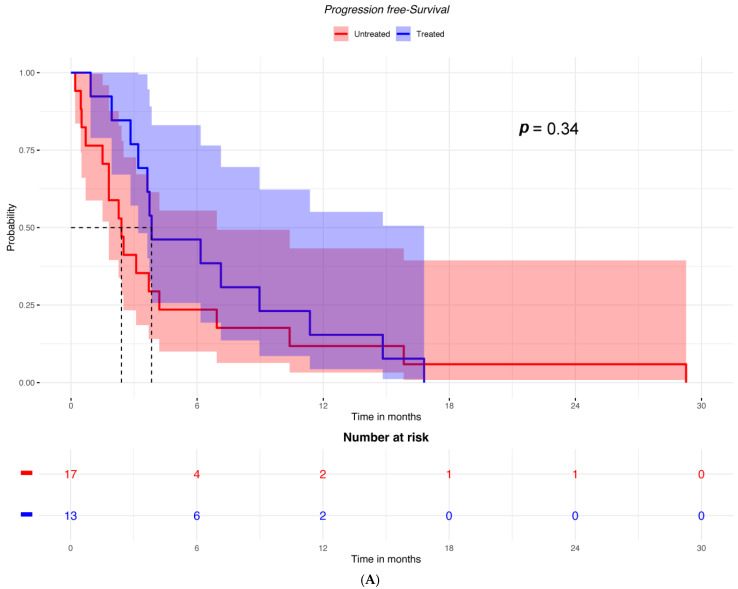

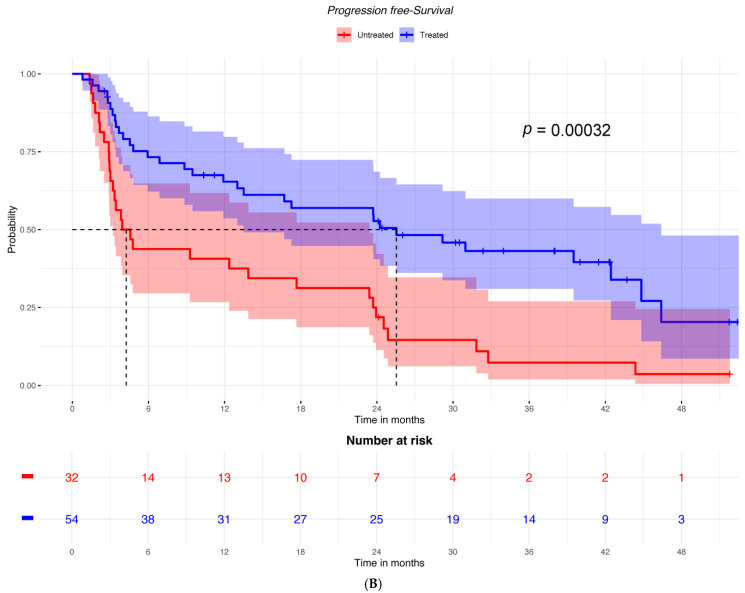
PFS according to having received or not received cetirizine in (**A**) patients with low ICOSLG expression (*n* = 30) and (**B**) patients with high ICOSLG expression (*N* = 86).

Among patients with low ICOSLG expression, PFS was not different between the two groups of patients with or without cetirizine (Figure 2A). In contrast, among patients with high ICOSLG expression, PFS was significantly better in those who had received cetirizine (Figure 2B).

**Figure 3:**
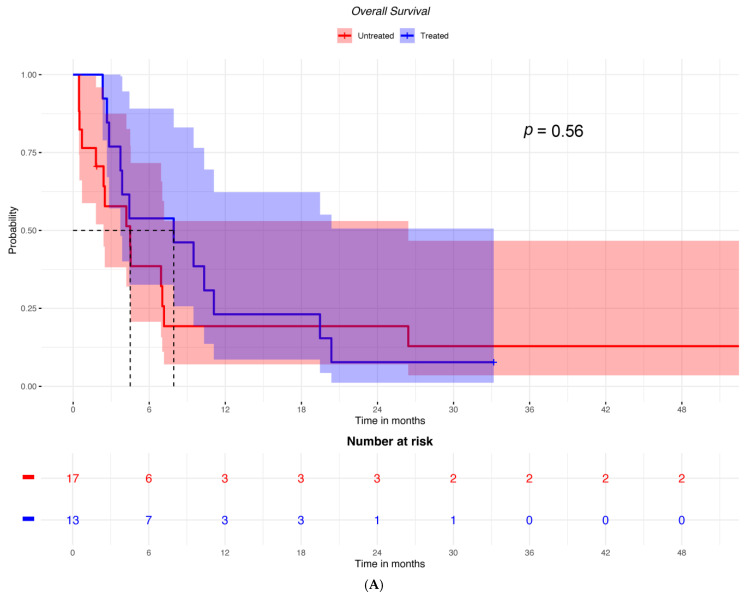

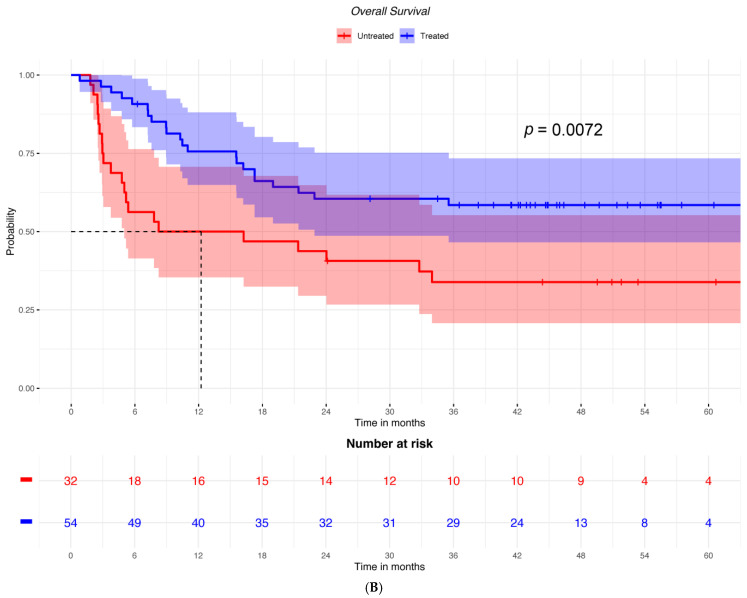
OS in patients treated or not treated with cetirizine and (**A**) with low ICOSLG expression (*n* = 30) or (**B**) with high ICOSLG expression (*N* = 86).

Accordingly, OS was not significantly different between groups treated with cetirizine versus untreated patients with low ICOSLG expression (HR = 0.789; 95% CI: 0.36–1.73) (Figure 3A). Conversely, among patients with high ICOSLG expression, it was higher in those who had received cetirizine (HR = 0.449; 95% CI: 0.25–0.82) (Figure 3B).

**Figure 4:**
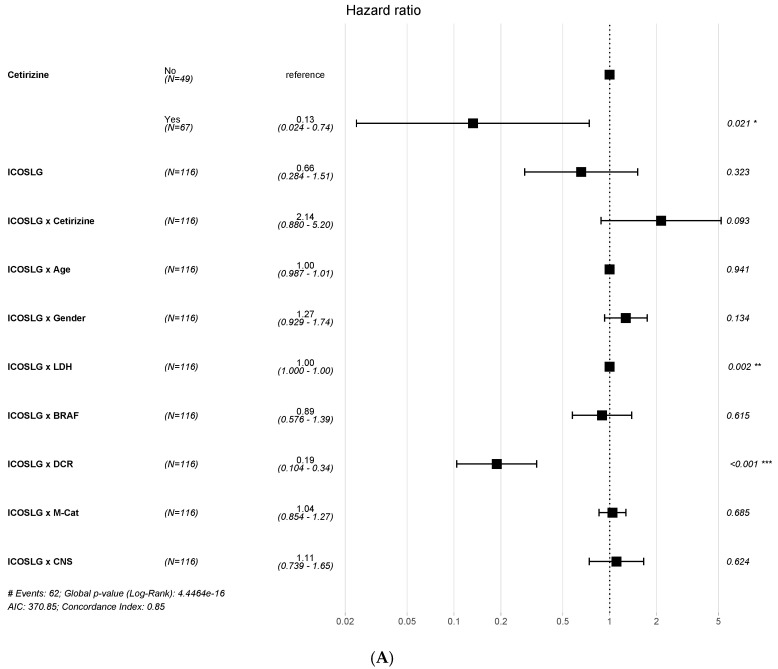

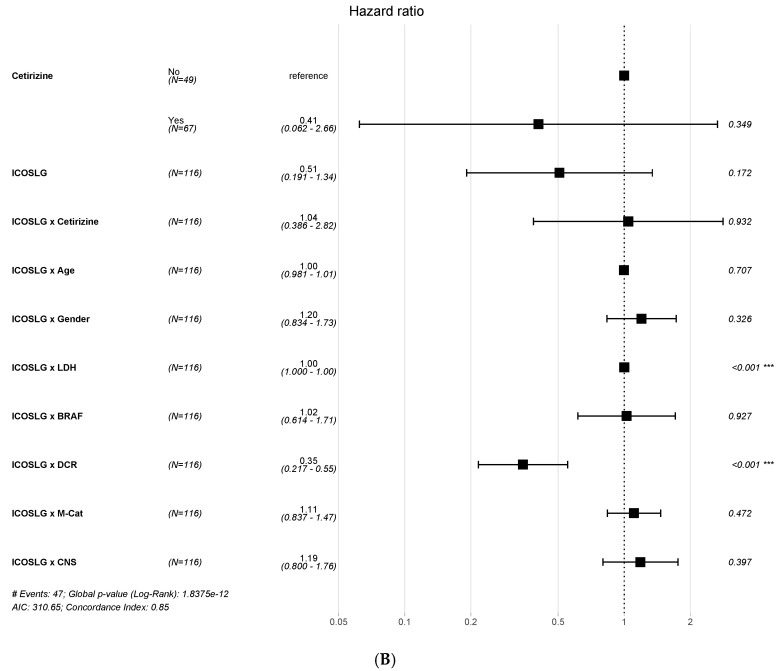
Multivariable survival analysis according to PFS (**A**) and OS (**B**) for ICOSLG expression. * *p* ≤ 0.05, ** *p* ≤ 0.01, *** *p* ≤ 0.001.

A multivariable analysis with a logistic regression model showed that patients treated with cetirizine had a better PFS than untreated ones (Figure 4A). Additionally, these results were confirmed by dichotomizing data, finding an improved DCR with cetirizine treatment in patients with high ICOSLG expression (Supplementary Figure S3A).

A similar association of ICOSLG expression was found for OS (Figure 4B and Supplementary Figure S3B). ICOSLG was associated with LDH expression, but the association was irrelevant as the HR was =1 for both PFS and OS.

Patients treated with cetirizine had a higher risk of colitis than the overall population (25.5% vs. 5.1%, *p* = 0.02), independent of ICOSLG expression. In comparison, patients with high ICOSLG expression had an increased risk of arthralgia (33.9% vs. 8.3%, *p* = 0.02), colitis (22.6% vs. 0, *p* = 0.01), pancreatitis (17.7% vs. 0, *p* = 0.03), and skin reactions (50% vs. 16.7%, *p* = 0.01).

The ICOSLG expression at baseline was associated with the blood expression of high-affinity IgG Fc receptor 1 (*FCGR1A*), coding for CD64, in the overall population (Rho = −0.219; *p* = 0.018) and patients treated with cetirizine (Rho = −0.287; *p* = 0.018) but not in the untreated ones. After 3 months, this association was not present in any subgroup.

## 3. Discussion

A previous study [12] demonstrated that patients with metastatic unresectable melanoma receiving cetirizine premedication with immunotherapy had better outcomes than those not receiving cetirizine. Since a proportion of patients had poorer results after immune checkpoint inhibitor (ICI) blockade despite cetirizine premedication, we conducted this retrospective study with the aim of identifying a predictive biomarker of the response to ICI blockade immunotherapy in patients with advanced unresectable stage IIIb–IV cutaneous melanoma with concomitant administration of cetirizine.

Circulating biomarkers and tumor molecular signatures have been previously investigated in advanced melanoma to identify patients with resistance hallmarks and a reduced probability of response to checkpoint inhibition. A tumor inflammation signature including 16 genes was associated with response to anti-PD-1 [16]; tumor mutational burden and microsatellite instability are considered predictive tissue markers of metastasis, based on results from KEYNOTE-158 [17,18,19]. Our transcriptomic analysis showed a gene expression signature associated with response to anti-PD-1 treatment in this patient population. The signature included 15 genes, but only ICOSLG was directly correlated to the DCR when detected with a loading value > 0.3 (Supplementary Figure S1). ICOSLG has a relevant role in immune antibody response modulation [20] and was a suitable candidate as a possible predictive biomarker for response to treatment. Therefore, we decided to select it for further analysis. The clinical relevance of ICOSLG expression was evaluated by comparing PFS and OS Kaplan–Meier curves of patients with high versus low gene expression; the findings showed that only when the ICOSLG expression is high, patients receiving cetirizine have better outcomes than those not receiving it. This result was confirmed by a multivariable model of logistic regression showing a positive association of the DCR with ICOSLG expression for PFS and OS in patients receiving cetirizine and anti-PD-1 versus those receiving only anti-PD-1. The advantage of receiving cetirizine in those with ICOSLG expression was present even though the cetirizine subgroup had a higher frequency of negative predictive factors such as *BRAF* mutation, an elevated LDH level, and CNS metastases. On the contrary, the association of ICOSLG with LDH had no association with survival.

We observed an association between ICOSLG expression and CD64 expression at baseline in the overall patient sample and those who received cetirizine concomitantly with ICI. However, this association was absent in subjects who had not received cetirizine, nor was it detected after 3 months of treatment in any subgroup.

ICOSLG is an immune checkpoint expressed by antigen-presenting cells and other cell types, including mesenchymal stem cells, endothelial cells, fibroblasts, and tumor cells [21,22]. It is a ligand for the T cell-specific cell surface receptor ICOS, and their interaction promotes T cell responses. ICOS is found in T regulatory cells in melanoma patients, and cells with high expression can induce diverse immune responses in responder cells depending on activation signals and cytokines in the microenvironment [23]. ICOSLG is more highly expressed by M2-type than M1-type macrophages, and the activity of M1 and M2 is modulated by ICOSLG activation [24].

CD64 is a functional high-affinity IgG Fc receptor coded by the *FCGR1A* gene in humans [25]. It is a specific marker of M1 macrophages, playing a role in immune functions, including phagocytosis, antigen presentation, degranulation, cytokine production, immune complex clearance, and inflammation (UniProt https://www.uniprot.org/uniprot/P12314, accessed on 7 February 2024) [26,27]. Its expression was increased in cetirizine-treated patient blood macrophages after 3 months of checkpoint inhibition compared with baseline, and CD64 expression correlated with genes linked to the interferon pathway [12].

We speculate that the high expression of both ICOSLG and CD64 before ICI treatment in a subset of patients may be linked to a baseline immune activation condition [28,29]. These subjects benefit from concomitant cetirizine treatment, which may promote M1 polarization [12,30] and reduce ICOSLG expression in immune cells, resulting in the loss of the ICOSLG/CD64 association after 3 months of treatment.

In conclusion, our results show that the clinical advantage of concomitant treatment with cetirizine during checkpoint inhibition in patients with malignant melanoma is associated with high expression of ICOSLG. This gene signature could predict the response to ICI blockade, and a prospective study is necessary to confirm this hypothesis. Additionally, treatment with cetirizine is associated with an increased risk of colitis, while high ICOSLG expression predicts a higher risk of colitis, arthralgia, pancreatitis, and skin reactions.

## 4. Materials and Methods

### 4.1. Study Design

A retrospective cohort study was carried out at the Istituto Nazionale Tumori–IRCCS–Fondazione “G. Pascale”, Naples, Italy, upon communication with the local Ethics Committee [protocol no. 32/22 oss]. The study was performed in accordance with the revised version of the Declaration of Helsinki (52nd WMA General Assembly, Edinburgh, UK, October 2000).

Data were retrieved from clinical records. Consecutive adult patients with histologically confirmed unresectable or metastatic melanoma (stage IIIb–IV), treated with an anti-PD-1 agent (nivolumab or pembrolizumab), either first-line or pretreated with ipilimumab, and aged over 18 years, were enrolled between April 2014 and June 2018. The study compared the group of patients exposed to cetirizine to the not exposed group. The concurrent use of cetirizine at each immunotherapy administration was retrospectively ascertained. Cetirizine was used as a premedication on the day of immune therapy (10 mg, once). B-RAF mutation status was assessed at baseline in tumor tissue samples. All patients provided their written informed consent. Raw data generated in this study have been deposited in Zenodo: https://doi.org/10.5281/zenodo.13838853.

### 4.2. Evaluation of Outcomes

RECIST 1.1 criteria were used to evaluate the tumor response as complete response (CR), partial response (PR), stable disease (SD), or progressive disease. The following parameters were collected at baseline: demographic data, concurrent use of cetirizine, history of allergy, *BRAF* mutation status, American Joint Committee on Cancer (AJCC) distant metastases category, lactate dehydrogenase (LDH) serum level, and the presence of brain metastases.

The treatment response was evaluated during the first tumor response assessment, conducted 3 months after starting immunotherapy. During the follow-up, the following data were collected: PFS (defined as the time from the administration of the first dose of an anti-PD-1 agent to documented radiological progression, death, or loss at follow-up, whichever occurred first), OS (defined as the time from the administration of the first dose of an anti-PD-1 agent to death or loss at follow-up, whichever occurred first), the disease control rate (DCR; defined as the sum of CR, PR, and SD > 1 year), and the objective response (OR; defined as the sum of CR and PR).

### 4.3. Transcriptomic Analysis

To conduct a gene expression profile analysis, baseline peripheral blood samples were collected from enrolled patients treated with an anti-PD-1 agent. RNA from whole blood was extracted using the QIAamp RNA Blood Mini Kit (Qiagen, Hilden, Germany). A quantification and purity assessment were performed with a NanoDrop spectrophotometer (Thermo Fisher Scientific Inc., Waltham, MA, USA). The purified RNA of PBMCs was used for hybridization and subjected to gene profiling analysis on NanoString nCounter through the PanCancer IO 360 panel [31], characterized by 770 human genes involved in the interplay between the tumor microenvironment and immune response. Gene expression data were normalized using nSolver Version 4.0 Software; NanoString counts were normalized to External RNA Controls Consortium (ERCC; https://www.nist.gov/programs-projects/external-rna-controls-consortium, accessed on 30 November 2023) technical controls and 30 housekeeping genes. Analyzed genes are reported in Supplementary Table S1.

### 4.4. Statistical Analysis

Demographic and clinical data were tabulated using descriptive statistics. PFS was calculated from the start of treatment with anti-PD-1 to the evidence of progressive disease or death, whichever occurred first; OS was calculated from the start of treatment with anti-PD-1 to death or censoring at the last follow-up. Survival times for groups were analyzed by univariate analysis and expressed using the Kaplan–Meier method; differences among curves were assessed by the log-rank test. Continuous interactions were analyzed by multivariable analysis using a Cox regression model, and hazard ratios (HRs) and their 95% CIs were estimated. Spearman’s Rho analysis was used to evaluate the associations between variables [32].

To identify the mRNA gene signatures associated with the patient’s response groups, a discriminant analysis for sparse data was performed via the partial least squares procedure. The sparse variant of the partial least squares discriminant analysis (PLS-DA) allows for the selection of the most predictive or discriminative features in the data to classify the samples [15,33].

The identified gene expression signature scores were used as synthetic risk indices; to define the subgroups of low and high risk, an optimal cut-point was selected by maximizing the area under the curve of the receiver operating characteristic performed by a cross-validation approach.

## Figures and Tables

**Table 1:** Demographic and clinical data at baseline.

	Cetirizine		
Characteristics	No, *n* = 49 ^1^	Yes, *n* = 67 ^1^	Overall, *n* = 116 ^1^
Age	66.00 (13.00)	65.00 (26.50)	66.00 (22.00)
Sex			
Female	22.0 (44.9%)	29.0 (43.3%)	51.0 (44.0%)
Male	27.0 (55.1%)	38.0 (56.7%)	65.0 (56.0%)
BRAF			
MUT	13.0 (27.7%)	9.0 (14.8%)	22.0 (20.4%)
WT	34.0 (72.3%)	52.0 (85.2%)	86.0 (79.6%)
Missing	2	6	8
LDH [U/L]	333.00 (235.50)	278.00 (204.75)	304.00 (260.50)
Missing	10	23	33
M-Category			
M0	2.0 (4.1%)	3.0 (4.5%)	5.0 (4.3%)
M1A	5.0 (10.2%)	9.0 (13.4%)	14.0 (12.1%)
M1B	6.0 (12.2%)	9.0 (13.4%)	15.0 (12.9%)
M1C	36.0 (73.5%)	46.0 (68.7%)	82.0 (70.7%)
CNS			
NO	31.0 (67.4%)	53.0 (81.5%)	84.0 (75.7%)
YES	15.0 (32.6%)	12.0 (18.5%)	27.0 (24.3%)
Missing	3	2	5
Anti-PD1 agent			
Nivolumab	32 (65.3%)	42 (62.7%)	74 (63.8%)
Pembrolizumab	17 (34.7%)	25 (37.3%)	42 (36.2%)
First-line treatment	39 (79.6%)	49 (73.1%)	88 (75.9%)
Pretreated with ipilimumab	10 (20.4%)	18 (26.9%)	28 (24.1%)

^1^ Median (IQR) or frequency (%).

## Data Availability

Availability of data and material: https://doi.org/10.5281/zenodo.13838853.

## Supplementary Materials

The following supporting information can be downloaded at: https://www.mdpi.com/article/10.3390/ijms252212439/s1.
