# Supplementary material for: ICOSLG Is Associated with Anti-PD-1 and Concomitant Antihistamine Treatment Response in Advanced Melanoma
Source: Int J Mol Sci. 2024 Nov 19;25(22):12439. doi: 10.3390/ijms252212439 (PMC11594639; doi:10.3390/ijms252212439)
Supplement: Supplementary file 1 [file ijms-25-12439-s001.zip › ijms-3265871-supplementary.pdf]

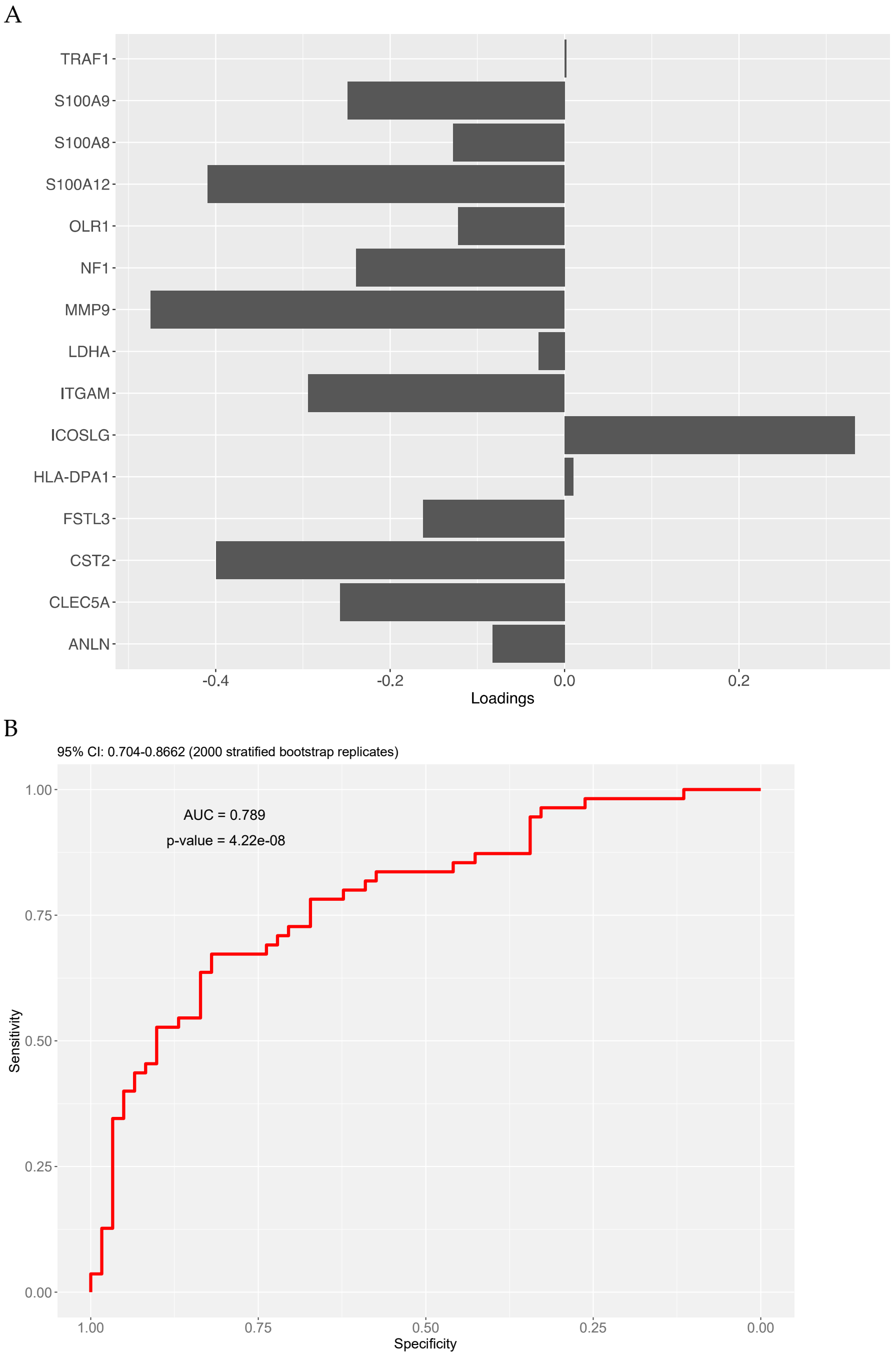

Supplementary Figure S1. A) Genes included in the signature associated with the response. B) Accuracy of the association between signature expression and DCR. Loading z value minimizes the equation y=zX, where y=signature (score, and x is the predictor (gene).

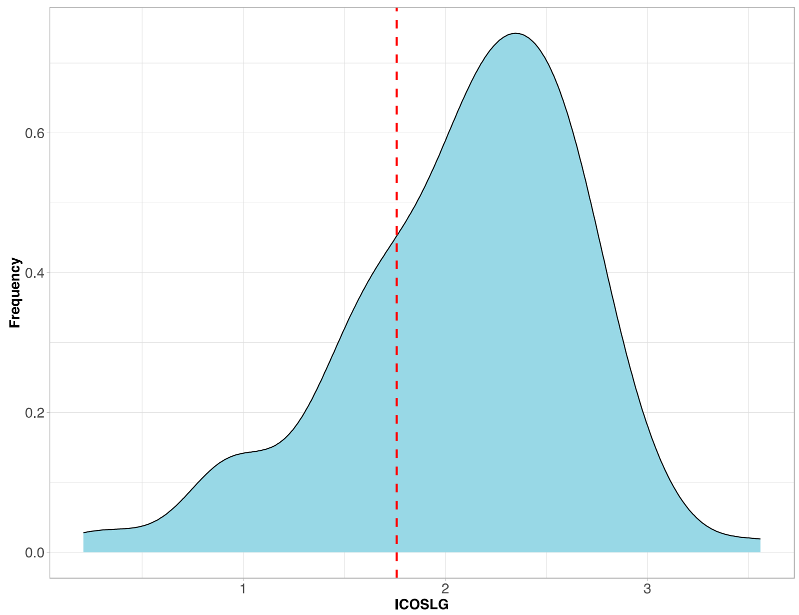

Supplementary Figure S2. Cut-off for the expression of the signature; Simon permutation approach.

A

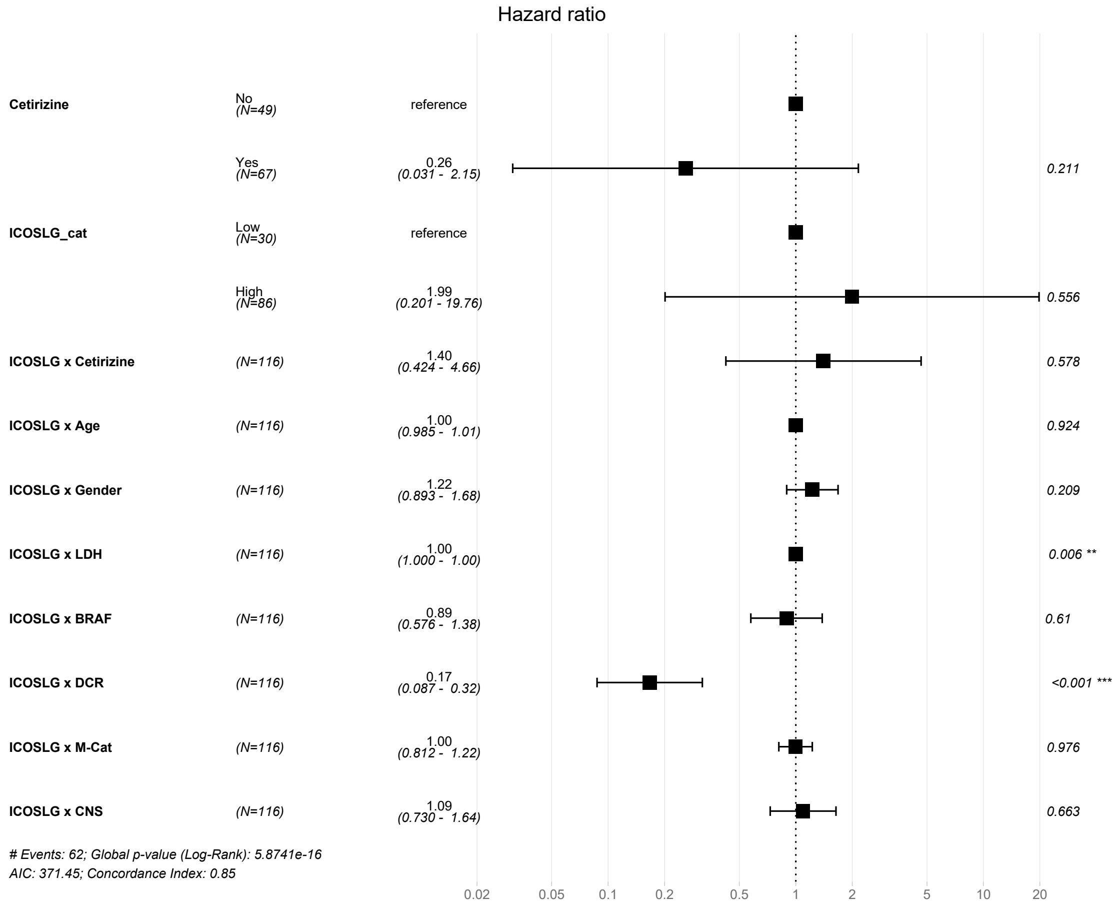

Hazard ratio
Cetirizine
No (N=49) reference
Yes (N=67) 0.26 (0.031 - 2.15) 0.211
ICOSLG_cat
Low (N=30) reference
High (N=86) 1.99 (0.201 - 19.76) 0.556
ICOSLG x Cetirizine (N=116) 1.40 (0.424 - 4.66) 0.578
ICOSLG x Age (N=116) 1.00 (0.985 - 1.01) 0.924
ICOSLG x Gender (N=116) 1.22 (0.893 - 1.68) 0.209
ICOSLG x LDH (N=116) 1.00 (1.000 - 1.00) 0.006 **
ICOSLG x BRAF (N=116) 0.89 (0.576 - 1.38) 0.61
ICOSLG x DCR (N=116) 0.17 (0.087 - 0.32) <0.001 ***
ICOSLG x M-Cat (N=116) 1.00 (0.812 - 1.22) 0.976
ICOSLG x CNS (N=116) 1.09 (0.730 - 1.64) 0.663
Events: 62; Global p-value (Log-Rank): 5.8741e-16
AIC: 371.45; Concordance Index: 0.85
0.02 0.05 0.1 0.2 0.5 1 2 5 10 20

B

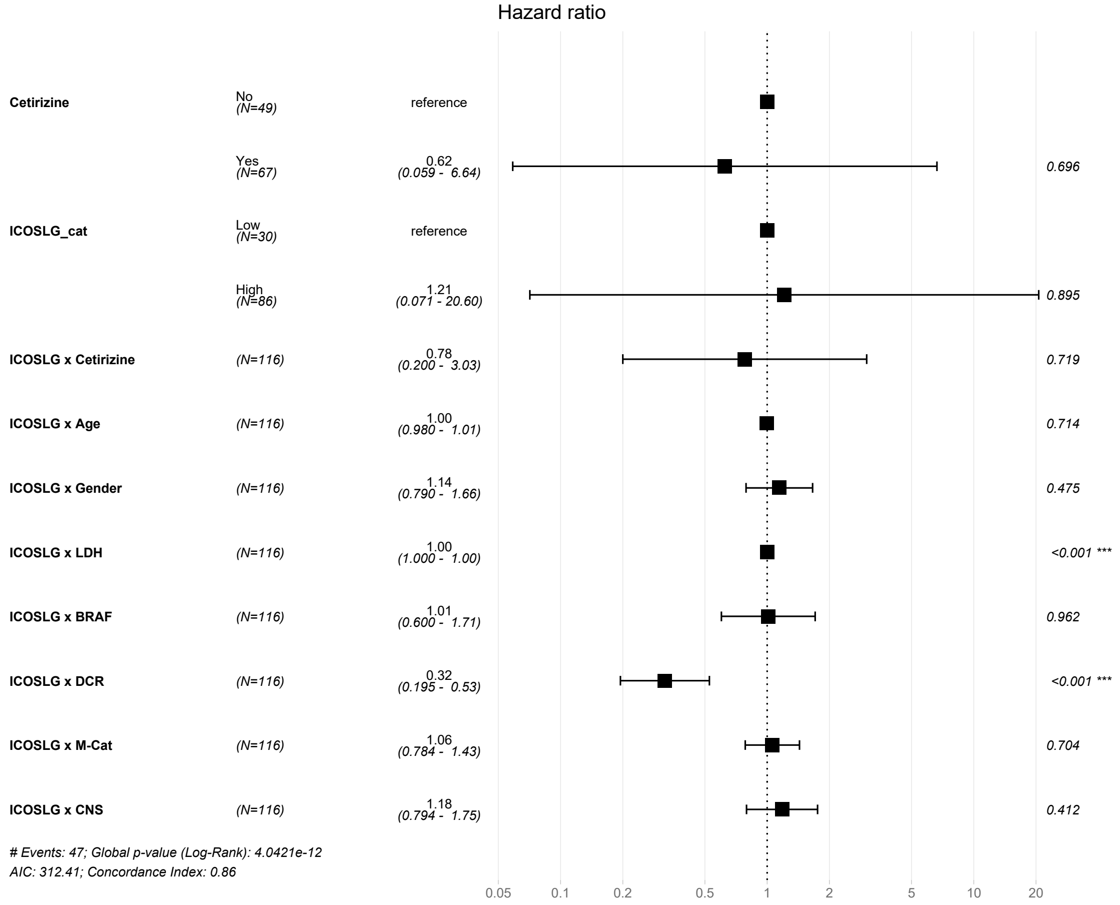

Supplementary Figure S3. Multivariable analysis with a logistic regression model with dichotomized data, A) PFS, B) OS.

Supplementary Table S1: List of analyzed genes

A2M	NM_000014.4	FWP007,S863-7,CPAMD5	TCCATCTCAATCCCTGTGAAGTCAGACATTGCTCCTGTCGCTCGGTTGCTCATCTATGCTGTTTTACCTACCGGGGACGTGATTGGGGATTCTGCAAAAT	alpha-2-macroglobulin
ACVR1C	NM_145259.2	activin A receptor, type IC;ALK7,ACVRLK7	GGAATTTTGCCACCATGTGACTTATTGGGGCAGAGAAAACTCAGGGTTGTCTTTGAGTCTGCACAAAAGCACCAGGGAACCTGCTTAGCAAATCGTCTGA	activin A receptor type 1C
ADAM12	NM_003474.5	a disintegrin and metalloproteinase domain 12 (meltrin alpha);MCMPMltna,MLTN;meltrin alpha	AGCAAAGAACTGATCATAAATCTGGAAAGAAATGAAGGTCTCATTGCCAGCAGTTTCACGGAAACCCACTATCTGCAAGACGGTACTGATGTCTCCCTCG	ADAM metallopeptidase domain 12
ADGRE1	NM_001256252.1	TM7LN3,EMR1;egf-like module containing, mucin-like, hormone receptor-like sequence 1,egf-like module containing, mucin-like, hormone receptor-like 1	GGAAAGCATGACACTGGCATCTTTTTGGAAACCCTCAGCAAATATCACTCCGGCTGTTCGGACGGAATACTTAGACATTGAGAGCAAAGTTATCAACAAA	adhesion G protein-coupled receptor E1
ADM	NM_001124.2	AM	CAGCGAGGTGTAAAGTTGTTCGCCGCGTGGAATGTGAGTGTGTTTGTGTGCATGAAAGAGAAAGACTGATTACCTCCTGTGTGGAAGAAGGAAACACCGA	adrenomedullin
ADORA2A	NM_000675.5	ADORA2;RDC8	GCCTGTCTCTTTGAGGATGTGGTCCCCATGAACTACATGGTGTACTTCAACTTCTTTGCCTGTGTGCTGGTGCCCCTGCTGCTCATGCTGGGTGTCTATT	adenosine A2a receptor
AKT1	NM_00516	v-akt murine thymoma viral oncogene homolog 1;RAC,PKB,PRKBA,AKT,RAC-alpha;protein kinase B	TATTTCATCCAGTTTGTTCTCCGGGTGTGGCCTCAGCCCTCAGAACAATCCGATTCACGTAGGGAAATGTTAAGGACTTCTGCAG	AKT serine/threonine kinase 1

	3.2		CTATGCGCAATGTGG		
ALDOA	NM_000034.3	aldolase A, fructose-bisphosphate	GAAGGCTGCGCAGGAGGAGTATGTCAAGCGAGCCCTGGCCAACAGCCTTGCCTGTCAAGGAAAGTACACTCCGAGCGGTCAGGCTGGGGCTGCTGCCAGC	aldolase, fructose-bisphosphate A	LOC112694756 (NM_001365307;NM_001365304;NM_001365305)
ALDOC	NM_005165.2	aldolase C, fructose-bisphosphate	TCAGTGCTGATGACCGTGTGAAAAAGTGCATTGGAGGCGTCATTTTCTTCCATGAGACCCTCTACCAGAAAGATGATAATGGTGTTCCCTTCGTCCGAAC	aldolase, fructose-bisphosphate C	
ANGPT1	NM_001199859.1	KIAA0003,Ang1	CTACTGGGCCTCCTCTCATAAAAGAGACAGTTGTTGGCAAGGTAGCAATACCAGTTTCAAACTTGGTGACTTGATCCACTATGCCTTAATGGTTTCCTCC	angiopoietin 1	
ANGPT2	NM_001147.2	Ang2	TCGCTCAAGGCCACAACCATGATGATCCGACCAGCAGATTTCTAAACATCCCAGTCCACCTGAGGAACTGTCTCGAACTATTTTCAAAGACTTAAGCCCA	angiopoietin 2	
ANGPTL4	NM_001039667.2	angiopoietin-like 4;pp1158,PGAR,ARP4,HFARP,FIAF,NL2;fasting-induced adipose factor,hepatic angiopoietin-related protein,PPARG angiopoietin related protein,hepatic fibrinogen/angiopoietin-related protein,peroxisome proliferator-activated receptor (PPAR) gamma induced angiopoietin-related protein,angiopoietin-related protein 4	CAGGGACAAGAACTGCGCCAAGAGCCTCTCTGGAGGCTGGTGGTTTGGCACCTGCAGCCATTCCAACCTCAACGGCCAGTACTTCCGCTCCATCCCACAG	angiopoietin like 4	
ANLN	NM_018685.2	anillin (Drosophila Scraps homolog), actin binding protein,anillin, actin binding protein (scraps homolog, Drosophila);ANILLIN,Scraps,scra	CGTGCCAGGCGAGAGAATCTTCAGAGAAAAATGGCTGAGAGGCCCACAGCAGCTCCAAGGTCTATGACTCATGCTAAGCGAGCTAGACAGCCACTTTCAG	anillin actin binding protein	

APC	NM_001127510.2	adenomatosis polyposis coli,adenomatous polyposis coli,APC, WNT signaling pathway regulator;DP2,DP3,DP2.5,PPP1R46;protein phosphatase 1, regulatory subunit 46	AATGTCCCTCCGTTCTTATGGAAGCCGGGAAGGATCTGTATCAAGCCGTTCTGGAGAGTGCAGTCCTGTTCCTATGGGTTCATTTCCAAGAAGAGGGTTT	APC regulator of WNT signaling pathway
APH1B	NM_001145646.1	anterior pharynx defective 1 homolog B (C. elegans),APH1B gamma secretase subunit;PSFL,APH-1B,DKFZp564D0372	GCTGGTGTCAGCCCAGACCTTCATAAGTTCTTATTATGGAATAAACCTGGCGTCAGCATTTATAATCCTGGTGCTCATGGGCACCTGGGCATTCTTAGCT	aph-1 homolog B, gamma-secretase subunit
API5	NM_001142930.1	AAC-11,API5L1,AAC11;API5-like 1,fibroblast growth factor 2-interacting factor 2,migration-inducing protein MIG8	AACTTCAGGTTCACCACCCAAGAAATCTTCAGCAGGACCAAAAAGAGATGCCAGGCAGATTTATAACCCTCCCAGTGGGAAATATAGCAGCAATTTGGGC	apoptosis inhibitor 5
APLNR	NM_005161.4	AGTRL1;angiotensin II receptor-like 1;FLJ90771,APJ,APJR;APJ (apelin) receptor	AGGCGCTCAGCTGATATCTTCATTGCTAGCCTGGCGGTGGCTGACCTGACCTTCGTGGTGACGCTGCCCCTGTGGGCTACCTACACGTACCGGGACTATG	apelin receptor
APOE	NM_000041.2	AD2;Alzheimer disease 2 (APOE*E4-associated, late onset)	GGGCTGCGTTGCTGGTCACATTCCTGGCAGGATGCCAGGCCAAGGTGGAGCAAGCGGTGGAGACAGAGCCGGAGCCCGAGCTGCGCCAGCAGACCGAGTG	apolipoprotein E
APOL6	NM_030641.3	APOL-VI,APOLVI	GGCTGATGGACAACCAGGCGGAGAGAGAAAGTGAGGCTGGTGTTGGTTTGCAAAGGGATGAGGATGACGCTCCTCTGTGTGAAGACGTGGAGCTACAAGA	apolipoprotein L6

AQP9	NM_001320635.1	SSC1,HsT17287	TATGTCCTTTGCTGGTGGAAAACTGCTGATCGTGGGAGAAAATGCAACAGCACACATTTTTGCAACATACCCAGCTCCGTATCTATCTCTGGCGAACGCA	aquaporin 9
AREG	NM_001657.3	SDGF,AREGB;schwannoma-derived growth factor,amphiregulin B	GAGAATGCAAATATATAGAGCACCTGGAAGCAGTAACATGCAAATGTCAGCAAGAATATTTCGGTGAACGGTGTGGGGAAAAGTCCATGAAAACTCACAG	amphiregulin
ARG1	NM_000045.3	arginase, liver	AAAGAAAAGGCCAATTCATCTAAGTTTTGATGTTGACGGACTGGACCCATCTTTCACACCAGCTACTGGCACACCAGTCGTGGGAGGTCTGACATACAGA	arginase 1
ARG2	NM_001172.3	arginase, type II	CACTGCCCAGACCTTTGTGTTGTCTGGGTTGATGCCCATGCTGACATCAACACACCCCTTACCACTTCATCAGGAAATCTCCATGGACAGCCAGTTTCAT	arginase 2
ARID1A	NM_006015.4	C1orf4,SMARCF1;SWI/SNF related, matrix associated, actin dependent regulator of chromatin, subfamily f, member 1,AT rich interactive domain 1A (SWI- like),AT rich interactive domain 1A (SWI-like);B120,P270,C10rf4,BAF250,BAF250a	CCATGCAGGGCCGGGCGCAGAGTGCCATGGGCGGCCTCTCTTATACACAGCAGATTCCTCCTTATGGACAACAAGGCCCCAGCGGGTATGGTCAACAGGG	AT-rich interaction domain 1A
ARNT2	NM_014862.3	KIAA0307,bHLHe1	CTGTATGCATGGACATGAATGGGATGTCGGTGCCCACAGAGTTCTTATCCCGGCATAACTCCGATGGAATCATCACATTTGTGGATCCAAGATGTATCAG	aryl hydrocarbon receptor nuclear translocator 2
ATF3	NM_0167		AAGATATTCAGGTGGCCAGAAGAGCTTGTCAGCAAGAGGAGGACAGAATTCTCCCAGCGTTAACACAAAATCCATGGGCAGT	activating transcription factor 3

	4.3		ATGATGGCAGGTCCTCTG	
ATM	NM_000051.3	ATA,ATDC,ATC,ATD;ataxia telangiectasia mutated (includes complementation groups A, C and D),ataxia telangiectasia mutated;TEL1,TELO1;TEL1, telomere maintenance 1, homolog (S. cerevisiae)	AGTACTTAATGATCTGCTTATCTGCTGCCGTCAACTAGAACATGATAGAGCTACAGAACGAAAGAAAGAAGTTGAGAAATTTAAGCGCCTGATTCGAGAT	ATM serine/threonine kinase
AXIN1	NM_181050.1	PPP1R49;protein phosphatase 1, regulatory subunit 49	AGCCCGATGGGGAACAGTGTTGGGTGTACCACCCATCCCTGTGGTCTACCCGTGTCTAGAGGCAGGTAGGGGGTCCCTCCAAGTGGTCCACAAGCTTCTG	axin 1
AXL	NM_001699.4	UFO,JTK11,Tyro7,ARK	TCGTGGCCGCTGCCTGTGTCCTCATCTTGGCTCTCTTCCTTGTCCACCGGCGAAAGAAGGAGACCCGTTATGGAGAAGTGTTTGAACCAACAGTGGAAAG	AXL receptor tyrosine kinase
B2M	NM_004048.2		TAATTCTACTTTGAGTGCTGTCTCCATGTTTGATGTATCTGAGCAGGTTGCTCCACAGGTAGCTCTAGGAGGGCTGGCAACTTAGAGGTGGGGAGCAGAG	beta-2-microglobulin
BAD	NM_004322.3	BCL2L8,BBC2	AGGGAGGGCTGACCCAGATTCCCTTCCGGTGCGTGTGAAGCCACGGAAGGCTTGGTCCCATCGGAAGTTTTGGGTTTTCCGCCCACAGCCGCCGGAAGTG	BCL2 associated agonist of cell death
BAMBI	NM_012342.2	BMP and activin membrane-bound inhibitor homolog (Xenopus laevis);NMA	AGCTGACTTCTTCCAAAGAGTTGTGGTTCCGGGCAGCGGTCATTGCCGTGCCCATTGCTGGAGGGCTGATTTTAGTGTTGCTTATTATGTTGGCCCTGAG	BMP and activin membrane bound inhibitor
BATF3	NM_018666	basic leucine zipper transcription factor, ATF-like 3;JUNDM1,SNFT,JDP1;Jun dimerization protein 1	TCCAGGACCAGCCAGCTGTGTTCCCTGCAGACTGGGCTCAGCCCGACATCCAACAGGCGCCAAACTCACAGAGCCCTTGTGCAG	basic leucine zipper ATF-like transcription factor 3

	4.2		ATCCAGCATGGAGGCC		
BAX	NM_004324.3	BCL2-associated X protein,BCL2 associated X protein;BCL2L4	CCCCGAGAGGTCTTTTTCCGAGTGGCAGCTGACATGTTTTCTGACGGCAACTTCAACTGGGGCCGGGTTGTCGCCCTTTTCTACTTTGCCAGCAAACTGG	BCL2 associated X, apoptosis regulator	
BBC3	NM_014417.4	JFY1,PUMA;p53-upregulated modulator of apoptosis	GGGGCCCGTGAAGAGCAAATGAGCCAAACGTGACCACTAGCCTCCTGGAGCCAGAGAGTGGGGCTCGTTTGCCGGTTGCTCCAGCCCGGCGCCCAGCCAT	BCL2 binding component 3	
BBS1	NM_024649.4	FLJ23590	TGGATGCGCACTACGACCCAATGGCCAATATCCACACCTTTTCTGCCTGCCTAGCGCTGGCAGATTTACATGGGGATGGGGAATACAAGCTGGTGGTAGG	Bardet-Biedl syndrome 1	
BCAT1	NM_005504.6	BCT1;branched chain aminotransferase 1, cytosolic	TACCAAAGCCCTGCTCTTTGTACTCTTGAGCCCAGTGGGACCTTATTTTTCAAGTGGAACCTTTAATCCAGTGTCCCTGTGGGCCAATCCCAAGTATGTA	branched chain amino acid transaminase 1	
BCL2	NM_000633.2	B-cell CLL/lymphoma 2,BCL2, apoptosis regulator;Bcl-2,PPP1R50;protein phosphatase 1, regulatory subunit 50	CCAAGCACCGCTTCGTGTGGCTCCACCTGGATGTTCTGTGCCTGTAAACATAGATTCGCTTTCCATGTTGTTGGCCGGATCACCATCTGAAGAGCAGACG	BCL2 apoptosis regulator	Isoform-specific Probe
BCL2L1	NM_001191.3	BCLX,BCL2L,Bcl-X,bcl-xL,bcl-xS,PPP1R52;protein phosphatase 1, regulatory subunit 52	CTGATTGGTGCAACCCTTACCCCTTAGCCTCCCTGAAAATGTTTTTCTGCCAGGGAGCTTGAAAGTTTTCAGAACCTCTTCCCCAGAAAGGAGACTAGAT	BCL2 like 1	
BCL6B	NM_181844	ZNF62;zinc finger protein 62,B-cell CLL/lymphoma 6, member B (zinc finger protein),B-cell CLL/lymphoma 6, member B,B cell CLL/lymphoma 6B,BCL6B, transcription repressor;ZBTB28,BAZF	AGTCCAGCCAGCCCTGACCCCAAGGCCTGCAACTGGAAAAAGTACAAGTACATCGTGCTAAACTCTCAGGCCTCCCAAGCAGGG	BCL6B transcription repressor	

	4.3		AGCCTGGTCGGGGAGA	
BID	NM_001196.3		CCTGGTAATAGCTGTAAACTTCCAATTTCTAGCCATACGCTCAGCTCATCCATGCCTCAGAAGTGCATCTGGAGAGAACAGGTTTCTAAGCATAAAAGAT	BH3 interacting domain death agonist
BIRC3	NM_001165.4	API2;baculoviral IAP repeat-containing 3;cIAP2,hiap-1,MIHC,RNF49,MALT2,c-IAP2;apoptosis inhibitor 2,TNFR2-TRAF signaling complex protein,mammalian IAP homolog C,inhibitor of apoptosis protein 1	TTTTGCCTCTGCTAAGAGGCTCTGCTGGCTACCCATGTACTAGCCAGTGTCCTGCATGGGTGCTAGGCTGAATTATTTGTAATTGTGCTTAGGTGATTTG	baculoviral IAP repeat containing 3
BIRC5	NM_001168.2	API4;apoptosis inhibitor 4,baculoviral IAP repeat-containing 5;EPR-1,survivin;survivin variant 3 alpha	CCATTCTAAGTCATTGGGGAAACGGGGTGAACTTCAGGTGGATGAGGAGACAGAATAGAGTGATAGGAAGCGTCTGGCAGATACTCCTTTTGCCACTGCT	baculoviral IAP repeat containing 5
BLK	NM_001330465.1	B lymphoid tyrosine kinase;MGC10442	ACAGGAAGAGAAGGCTATGTGCCCAGTAACTTTGTGGCCCGAGTGGAGAGCCTGGAAATGGAAAGGTGGTTCTTTAGATCACAGGGTCGGAAGGAGGCTG	BLK proto-oncogene, Src family tyrosine kinase
BLM	NM_000057.3	Bloom syndrome,Bloom syndrome RecQ like helicase;BS,RECQL3,RECQ2	GGAGGGTGATTCCTGCCCTACAGGGAATTCTATGAAGGAGTTAAATTTTCACACCTTCCCTCAAATTCTGTTTCTCCTGGGGACTGTTTACTGACTACC	BLM RecQ like helicase
BMP2	NM_001200.3	BMP2A	AGTTCTATCCCCACGGAGGAGTTTATCACCTCAGCAGAGCTTCAGGTTTTCCGAGAACAGATGCAAGATGCTTTAGGAAACAATAGCAGTTTCCATCACC	bone morphogenetic protein 2
BNIP3	NM_004	BCL2/adenovirus E1B 19kDa interacting protein 3;Nip3;nineteen kD interacting protein-3	CGCTCCGACCTCCGCTTTCCCACCGCCCGCAGCTGAAGCACATCCCGCAGCCCGGCGCG	BCL2 interacting protein 3

	052.3		GACTCCGATCGCCGCAGTTGCCCTCTGGCGCCATGTCGCAG	
BNIP3L	NM_001330491.1	BCL2/adenovirus E1B 19kDa interacting protein 3 like;Nix,BNIP3a;NIP-3-like protein X	CCTGAATAAAATTGGAAAGACTGGAAAGTTAGGAGAACTGACTAGCTAAACTGCTACAGTATGCAATTTCTATTACAATTGGTATTACAGGGGGGAAAAG	BCL2 interacting protein 3 like
BRCA1	NM_007294.3	breast cancer 1, early onset,breast cancer 1;RNF53,BRCC1,PPP1R53,FANCS;BRCA1/BRCA2-containing complex, subunit 1,protein phosphatase 1, regulatory subunit 53,Fanconi anemia, complementation group S	TAGTTCTACCAGTAAAAATAAAGAACCAGGAGTGGAAAGGTCATCCCCTTCTAAATGCCCATCATTAGATGATAGGTGGTACATGCACAGTTGCTCTGGG	BRCA1 DNA repair associated
BRCA2	NM_000059.3	FANCD1,FACD,FANCD;Fanconi anemia, complementation group D1,breast cancer 2, early onset,breast cancer 2;FAD,FAD1,BRCC2,XRCC11;BRCA1/BRCA2-containing complex, subunit 2	ATCTCCAAGGAAGTTGTACCGTCTTTGGCCTGTGAATGGTCTCAACTAACCCTTTCAGGTCTAAATGGAGCCCAGATGGAGAAAATACCCCTATTGCATA	BRCA2 DNA repair associated
BRD3	NM_007371.3	bromodomain-containing 3;RING3L,ORFX,KIAA0043;RING3-like	AACTTCAAATGCTAACCCGATGACCCCAGAAAACCGTGTGAGATTCGTACCGAAGAACCTTGTGGAATCCCTTTGCTTAGGCCCAACCTGGTCGATAGCT	bromodomain containing 3
BRD4	NM_001330384.1	bromodomain-containing 4;HUNKI,MCAP,CAP,HUNK1;chromosome-associated protein,mitotic chromosome-associated protein	ATGAGTCGGAGGAAGAGGACAAGTGCAAGCCTATGTCCTATGAGGAGAAGCGGCAGCTCAGCTTGGACATCAACAAGCTCCCCGGCGAGAAGCTGGGCCG	bromodomain containing 4
BRIP1	NM_032043.2	OF,BACH1,FANCJ;BRCA1/BRCA2-associated helicase 1	TAGATAGTATGGTCAACAATAATATAAGGAAGAAAGATCATGAACCCCTACGAGCTGTGTGCTGTAGCCTCATTAATTGGTTAGAAGCAAACGCTGAATA	BRCA1 interacting protein C-terminal helicase 1

BTLA	NM_001085357.1	BTLA1,CD272	GAAATACTGTGCTAACAGGCCTCATGTGACTTGGTGCAAGCTCAATGGAACAACATGTGTAAAACTTGAAGATAGACAAACAAGTTGGAAGGAAGAGAAG	B and T lymphocyte associated
C1QA	NM_015991.3	complement component 1, q subcomponent, alpha polypeptide,complement component 1, q subcomponent, A chain,complement C1q chain A	CAGAACCACTCCGGCCGATTCGTCTGCACTGTACCCGGCTACTACTACTTCACCTTCCAGGTGCTGTCCCAGTGGGAAATCTGCCTGTCCATCGTCTCCT	complement C1q A chain
C1QB	NM_000491.3	complement component 1, q subcomponent, beta polypeptide,complement component 1, q subcomponent, B chain,complement C1q chain B	AAGAACTCACTACTGGGCATGGAGGGTGCCAACAGCATCTTTTCCGGGTTCCTGCTCTTTCCAGATATGGAGGCCTGACCTGTGGGCTGCTTCACATCCA	complement C1q B chain
C2	NM_000063.5	complement component 2	CCAACCCAGGCATTTCACTGGGCGCAGTGCGGACAGGCTTCCGCTTTGGTCATGGGGACAAGGTCCGCTATCGCTGCTCCTCGAATCTTGTGCTCACGGG	complement C2
C5	NM_001317163.1	complement component 5;CPAMD4,C5a,C5b;prepro-C5,C5a anaphylatoxin	CTTGGAAAGAGTATTTCAATTCTTAGAGAAGAGTGATCTGGGCTGTGGGGCAGGTGGTGGCCTCAACAATGCCAATGTGTTCCACCTAGCTGGACTTACC	complement C5
C5AR1	NM_001736.3	C5R1;complement component 5 receptor 1 (C5a ligand),complement component 5a receptor 1;C5A,C5AR,CD88	GCTGACCATACCCTCCTTCCTGTACCGGGTGGTCCGGGAGGAGTACTTTCCACCAAAGGTGTTGTGTGGCGTGGACTACAGCCACGACAAACGGCGGGAG	complement C5a receptor 1
C7	NM_00	complement component 7	CCTTGTCAAAATGGTGGTTTGGCTACTGTTGAGGGGACCCATTGTCTG	complement C7

	0587.2		TGCCATTGCAAACCGTACACATTTGGTGCGGCGTGTGAGCAAGGAGTCCTCG	
CASP1	NM_001223.4	IL1BC;caspase 1, apoptosis-related cysteine protease (interleukin 1, beta, convertase),caspase 1, apoptosis-related cysteine peptidase (interleukin 1, beta, convertase),caspase 1, apoptosis-related cysteine peptidase;ICE;caspase-1,interleukin 1, beta, convertase	TGCAAGAATATGCCTGTTCCTGTGATGTGGAGGAAATTTTCCGCAAGGTTCGATTTTCATTTGAGCAGCCAGATGGTAGAGCGCAGATGCCCACCACTGA	caspase 1
CASP3	NM_032991.2	caspase 3, apoptosis-related cysteine protease,caspase 3, apoptosis-related cysteine peptidase;CPP32,CPP32B,Yama,apopain	CCCTCTCATTTTGACCTACTCTCATGCTGCAGAGGGTACTTTAAGACATACTCCTTCCATCAAATAGAACCACTATGAAGCTACCTCAAACTTCCAGTCA	caspase 3
CASP8	NM_001228.4	caspase 8, apoptosis-related cysteine protease,caspase 8, apoptosis-related cysteine peptidase;MCH5,MACH,FLICE,Casp-8	TTTCTTTTGCAAGAGGAAATCTCCAAATGCAAACTGGATGATGACATGAACCTGCTGGATATTTTCATAGAGATGGAGAAGAGGGTCATCCTGGGAGAAG	caspase 8
CASP9	XM_011542270.1	caspase 9, apoptosis-related cysteine protease,caspase 9, apoptosis-related cysteine peptidase;MCH6,ICE-LAP6,APAF-3,PPP1R56;protein phosphatase 1, regulatory subunit 56	GGCCAGGCAGCTGATCATAGATCTGGAGACTCGAGGGAGTCAGGCTCTTCCTTTGTTCATCTCCTGCTTAGAGGACACAGGCCAGGACATGCTGGCTTCG	caspase 9
CBLC	NM_001130852.1	Cas-Br-M (murine) ectropic retroviral transforming sequence c,Cas-Br-M (murine) ecotropic retroviral transforming sequence c,Cbl proto-oncogene C, E3 ubiquitin protein ligase;CBL-3,CBL-SL,RNF57	GACCTCACCTGCAGCGGGCACGTGTCCATCTTCGAGTTCGACGTCTTCACCAGGCTCTTTCAGCCATGGCCAACACTCCTCAAGAACTGGCAGCTCCTGG	Cbl proto-oncogene C
CCL13	NM_00540	SCYA13;small inducible cytokine subfamily A (Cys-Cys), member 13,chemokine (C-C motif) ligand 13;MCP-4,NCC-1,SCYL1,CKb10,MGC17134	CCCCAGGGACTTGCTCAGCCAGATGCACTCAACGTCCCATCTACTTGCTGCTTCACATTTAGCAGTAAGAAGATCTCCTTGCAG	C-C motif chemokine ligand 13

	8.2		AGGCTGAAGAGCTATG	
CCL14	NM_032962.4	SCYA14;small inducible cytokine subfamily A (Cys-Cys), member 14,chemokine (C-C motif) ligand 14;HCC-1,HCC-3,NCC-2,SCYL2,CKb1,MCIF	AAGATCCCGCGTCAGCGGATTATGGATTACTATGAGACCAACAGCCAGTGCTCCAAGCCCGGAATTGTCTTCATCACCAAAAGGGGCCATTCCGTCTGTA	C-C motif chemokine ligand 14
CCL18	NM_002988.3	SCYA18;small inducible cytokine subfamily A (Cys-Cys), member 18, pulmonary and activation-regulated,chemokine (C-C motif) ligand 18 (pulmonary and activation-regulated),chemokine (C-C motif) ligand 18;DC-CK1,PARC,AMAC-1,DCCK1,MIP-4,CKb7;pulmonary and activation-regulated	GACCACTTCTCTGCCTGCCCAGCATCATGAAGGGCCTTGCAGCTGCCCTCCTTGTCCTCGTCTGCACCATGGCCCTCTGCTCCTGTGCACAAGTTGGTAC	C-C motif chemokine ligand 18
CCL19	NM_006274.2	SCYA19;small inducible cytokine subfamily A (Cys-Cys), member 19,chemokine (C-C motif) ligand 19;ELC,MIP-3b,exodus-3,CKb11;CC chemokine ligand 19,macrophage inflammatory protein 3-beta,beta chemokine exodus-3,CK beta-11,EBI1-ligand chemokine	CCTCCATGGCCCTGCTACTGGCCCTCAGCCTGCTGGTTCTCTGGACTTCCCCAGCCCCAACTCTGAGTGGCACCAATGATGCTGAAGACTGCTGCCTGTC	C-C motif chemokine ligand 19
CCL2	NM_002982.3	SCYA2;small inducible cytokine A2 (monocyte chemotactic protein 1, homologous to mouse Sig-je),chemokine (C-C motif) ligand 2;MCP1,MCP-1,MCAF,SMC-CF,GDCF-2,HC11,MGC9434;monocyte chemotactic protein 1, homologous to mouse Sig-je,monocyte chemoattractant protein-1,monocyte chemotactic and activating factor,monocyte secretory protein JE,small inducible cytokine subfamily A (Cys-Cys), member 2	CCCCAAGCAGAAGTGGGTTCAGGATTCCATGGACCACCTGGACAAGCAAACCCAAACTCCGAAGACTTGAACACTCACTCCACAACCCAAGAATCTGCAG	C-C motif chemokine ligand 2
CCL20	NM_001130046.1	SCYA20;small inducible cytokine subfamily A (Cys-Cys), member 20,chemokine (C-C motif) ligand 20;LARC,MIP-3a,exodus-1,ST38,CKb4	CCAAAACAGACTTGGGTGAAATATATTGTGCGTCTCCTCAGTAAAAAAGTCAAGAACATGTAAAAACTGTGGCTTTTCTGGAATGGAATTGGACATAGCC	C-C motif chemokine ligand 20
CCL21	NM_002989.2	SCYA21;small inducible cytokine subfamily A (Cys-Cys), member 21,chemokine (C-C motif) ligand 21;SLC,exodus-2,TCA4,CKb9,6Ckine,ECL;beta chemokine exodus-2,secondary lymphoid tissue chemokine,Efficient Chemoattractant for Lymphocytes	TGATGGAGGGGCTCAGGACTGTTGCCTCAAGTACAGCCAAAGGAAGATTCCCGCCAAGGTTGTCCGCAGCTACCGGAAGCAGGAACCAAGCTTAGGCTGC	C-C motif chemokine ligand 21
CCL22	NM_002	SCYA22;small inducible cytokine subfamily A (Cys-Cys), member 22,chemokine (C-C motif) ligand 22;MDC,STCP-1,ABCD-1,DC/B-CK,A-152E5.1,MGC34554	CTCGCCCAAGCAGCTGGTAATTCCATTTCATGTATTAGATGTCCCCTGGCCCTCTGTCCCCT	C-C motif chemokine ligand 22

	990.3		CTTAATAACCCTAGTCACAGTCTCCGCAGATTCTTGGG		
CCL3/L1	NM_021006.5	SCYA3;small inducible cytokine A3 (homologous to mouse Mip-1a),chemokine (C-C motif) ligand 3;G0S19-1,LD78ALPHA,MIP-1-alpha	GGGGAGGAGCAGGAGCCTGAGCCTTGGGAACATGCGTGTGACCTCTACAGCTACCTCTTCTATGGACTGGTTATTGCCAAACAGCCACACTGTGGGACTC		CCL3L3 (NM_001001437)
CCL4	NM_002984.2	LAG1,SCYA4;small inducible cytokine A4 (homologous to mouse Mip-1b),chemokine (C-C motif) ligand 4;MIP-1-beta,Act-2,AT744.1	TTCTGCAGCCTCACCTCTGAGAAAACCTCTTTGCCACCAATACCATGAAGCTCTGCGTGACTGTCCTGTCTCTCCTCATGCTAGTAGCTGCCTTCTGCTC	C-C motif chemokine ligand 4	CCL4L1 (NM_207007), CCL4L2 (NM_001291474;NM_001291471;NM_001291475;NM_001291469;NM_001291472;NM_001291468;NM_001291473;NM_001291470)
CCL5	NM_002985.2	D17S136E,SCYA5;small inducible cytokine A5 (RANTES),chemokine (C-C motif) ligand 5;RANTES,SISd,TCP228,MGC17164;T-cell specific protein p288,T-cell specific RANTES protein,SIS-delta,regulated upon activation, normally T-expressed, and presumably secreted,beta-chemokine RANTES,small inducible cytokine subfamily A (Cys-Cys), member 5	CCAAGTGTGTGCCAACCCAGAGAAGAAATGGGTTCGGGAGTACATCAACTCTTTGGAGATGAGCTAGGATGGAGAGTCCTTGAACCTGAACTTACACAAA	C-C motif chemokine ligand 5	
CCL7	NM_006273.3	SCYA6,SCYA7;small inducible cytokine A7 (monocyte chemotactic protein 3),chemokine (C-C motif) ligand 7;MCP-3,NC28,FIC,MARC,MCP3;monocyte chemoattractant protein 3,monocyte chemotactic protein 3	TAAAGCCTTGGATGTATATGTCATCTCAGTGCTGTAAAAACTGTGGGATGCTCCTCCCTTCTCTACCTCATGGGGGTATTGTATAAGTCCTTGCAAGAAT	C-C motif chemokine ligand 7	
CCL8	NM_005623.2	SCYA8;small inducible cytokine subfamily A (Cys-Cys), member 8 (monocyte chemotactic protein 2),chemokine (C-C motif) ligand 8;MCP-2,HC14	GAGCTCAACTAAGTTCACGGCAAAATGTCATTGTTCTCCCTCCTACCTGTCTGTAGTGTTGTGGGGTCCTCCCATGGATCATCAAGGTGAAACACTTTGG	C-C motif chemokine ligand 8	
CCNA1	NM_001111045.1	CT146	TTCCCCTATGCTGGTAGATTCATCTCTCCTCTCCCAGTCTGAAGATATATCCAGTCTTGGCACAGATGTGATAAATGTGACTGAATATGCTGAAGAAATT	cyclin A1	

CCNB1	NM_031966.2	CCNB;G2/mitotic-specific cyclin B1	GAGACAACTTGAGGAAGAGCAAGCAGTCAGACCAAAATACCTACTGGGTCGGGAAGTCACTGGAAACATGAGAGCCATCCTAATTGACTGGCTAGTACAG	cyclin B1
CCND1	NM_053056.2	BCL1,D11S287E,PRAD1;cyclin D1 (PRAD1: parathyroid adenomatosis 1);U21B31;parathyroid adenomatosis 1,B-cell CLL/lymphoma 1,G1/S-specific cyclin D1	CTGAGGAGCCCCAACAACTTCCTGTCCTACTACCGCCTCACACGCTTCCTCTCCAGAGTGATCAAGTGTGACCCGGACTGCCTCCGGGCCTGCCAGGAGC	cyclin D1
CCND2	NM_001759.2	G1/S-specific cyclin D2	AGCCTGCATCCCTTCGCCTGCAGCCTACTTTGGGGAAATAAAGTGCCTTACTGACTGTAGCCATTACAGTATCCAATGTCTTTTGACAGGTGCCTGTCCT	cyclin D2
CCND3	NM_001136017.2		CCATGTACCCGCCATCCATGATCGCCACGGGCAGCATTGGGGCTGCAGTGCAAGGCCTGGGTGCCTGCTCCATGTCCGGGGATGAGCTCACAGAGCTGCT	cyclin D3
CCNE1	NM_001238.1	CCNE;cyclin Es,cyclin Et	GAGAACTGTGTCAAGTGGATGGTTCCATTTGCCATGGTTATAAGGGAGACGGGGAGCTCAAAACTGAAGCACTTCAGGGGCGTCGCTGATGAAGATGCAC	cyclin E1
CCNO	NM_021147.4	CCNU;cyclin U;UDG2,FLJ22422,UNG2	GCTGGAGGACTGTATGGGCAAGTTGCAGCTGCTGGTGGCCATAAACAGTACTTCCTTGACTCACATGCTGCCCGTTCAGATCTGCGAGAAGTGCAGCCTG	cyclin O
CCR2	NM_001123.2	CMKBR2;chemokine (C-C motif) receptor 2;CC-CKR-2,CKR2,MCP-1-R,CD192,FLJ78302	GAGAAAGTGGATTGAACAAGGACGCATTTCCCCAGTACATCCACAACATGCTGTCCACATCTCGTTCTCGGTTTATCAGAAATA	C-C motif chemokine receptor 2

	3041.2		CCAACGAGAGCGGTGA	
CCR4	NM_005508.4	chemokine (C-C motif) receptor 4;CC-CKR-4,CMKBR4,CKR4,k5-5,ChemR13,CD194	TCCCTTCCTGGCTTTCTGTTCAGCACTTGTTATACTGAGCGCAACCATACCTACTGCAAAACCAAGTACTCTCTCAACTCCACGACGTGGAAGGTTCTCA	C-C motif chemokine receptor 4
CCR5	NM_000579.3	CMKBR5;chemokine (C-C motif) receptor 5,chemokine (C-C motif) receptor 5 (gene/pseudogene),C-C motif chemokine receptor 5 (gene/pseudogene);CKR-5,CC-CKR-5,CKR5,CD195,IDDM22	CAAGTGTCAAGTCCAATCTATGACATCAATTATTATACATCGGAGCCCTGCCAAAAAATCAATGTGAAGCAAATCGCAGCCCGCCTCCTGCCTCCGCTCT	C-C motif chemokine receptor 5
CD14	NM_001174105.1	CD14 antigen	GCCCCACAGCCTAGACCTCAGCCACAACTCGCTGCGCGCCACCGTAAACCCTAGCGCTCCGAGATGCATGTGGTCCAGCGCCCTGAACTCCCTCAATCTG	CD14 molecule
CD163	NM_203416.2	CD163 antigen;M130,MM130,SCARI1	CATCTTTCACTCTGCCCAGTAGCACCCCGCCCAGAAGGAACTTGTAGCCACAGCAGGGATGTTGGAGTAGTCTGCTCAAGATACACAGAAATTCGCTTGG	CD163 molecule
CD19	NM_001770.4	CD19 antigen	AGATTCACACCTGACTCTGAAATCTGAAGACCTCGAGCAGATGATGCCAACCTCTGGAGCAATGTTGCTTAGGATGTGTGCATGTGTGTAAGTGTGTGTG	CD19 molecule
CD1C	NM_001765.2	CD1;CD1C antigen, c polypeptide,CD1c antigen	CCTCTTTGGATTAACTCGGGAGATTCAAGACCATGCAAGTCAAGATTACTCGAAATATCCCTTTGAAGTACAGGTGAAAGCGGGCTGTGAGCTGCATTCT	CD1c molecule

CD2	NM_001767.3	SRBC;CD2 antigen (p50), sheep red blood cell receptor	GGAGGCAGCCTCTTGATGGTCTTTGTGGCACTGCTCGTTTTCTATATCACCAAAAGGAAAAAACAGAGGAGTCGGAGAAATGATGAGGAGCTGGAGACAA	CD2 molecule
CD209	NM_021155.2	CD209 antigen;DC-SIGN,CDSIGN,DC-SIGN1,CLEC4L	TTATCTCATACATGCAAACCTACCATCTGTTCAACTTCCACCTACCACCTCCTGCACCCCTTTGATCGGGGACTTACTGGTTGCAAGAGCTCATTTTGCA	CD209 molecule
CD244	NM_001166663.1	natural killer cell receptor 2B4,CD244 natural killer cell receptor 2B4,CD244 molecule, natural killer cell receptor 2B4;2B4,NAIL,NKR2B4,Nmrk,SLAMF4	CGAAGATGTCAAGGATCTGAAAACCAGGAGAAATCACGAGCAGGAGCAGACTTTTCCTGGAGGGGGGAGCACCATCTACTCTATGATCCAGTCCCAGTCT	CD244 molecule
CD247	NM_198053.2	CD3Z;CD3z antigen, zeta polypeptide (TiT3 complex),CD247 antigen;CD3H,CD3Q;T-cell surface glycoprotein CD3 zeta chain	CCGCGTACCAGCAGGGCCAGAACCAGCTCTATAACGAGCTCAATCTAGGACGAAGAGAGGAGTACGATGTTTTGGACAAGAGACGTGGCCGGGACCCTGA	CD247 molecule
CD27	NM_001242.4	TNFRSF7;tumor necrosis factor receptor superfamily, member 7;S152,Tp55	GCTGCCAGATGTGTGAGCCAGGAACATTCCTCGTGAAGGACTGTGACCAGCATAGAAAGGCTGCTCAGTGTGATCCTTGCATACCGGGGGTCTCCTTCTC	CD27 molecule
CD274	NM_014143.3	PDCD1LG1;programmed cell death 1 ligand 1,CD274 antigen;B7-H,B7H1,PD-L1,PDL1,B7-H1;B7 homolog 1	AAGGATACTTCTGAACAAGGAGCCTCCAAGCAAATCATCCATTGCTCATCCTAGGAAGACGGGTTGAGAATCCCTAATTTGAGGGTCAGTTCCTGCAGAA	CD274 molecule
CD276	NM_00102	CD276 antigen;B7-H3,B7H3,B7RP-2	TACATTTCTTAGGGACACAGTACACTGACCACATCACCACCCTCTTCTTCCAGTGCTGCGTGGACCATCTGGCTGCCTTTTTTCTC	CD276 molecule

	47366.1		CAAAAGATGCAATA	
CD28	NM_001243077.1	CD28 antigen (Tp44);T-cell-specific surface glycoprotein	GTAACAGTGGCCTTTATTATTTTCTGGGTGAGGAGTAAGAGGAGCAGGCTCCTGCACAGTGACTACATGAACATGACTCCCCGCCGCCCCGGGCCCACCC	CD28 molecule
CD300A	NM_001256841.1	CD300a antigen;Irp60,CMRF35H,CMRF-35-H9,IRC1,IRC2,IGSF12	AATGACACCTGCAAGTATCACTGCGGCCAAGACCTCAACAATCACAACTGCATTTCCACCTGTATCATCCACTACCCTGTTTGCAGTGGGTGCCACCCAC	CD300a molecule
CD36	NM_001001548.2	CD36 antigen (collagen type I receptor, thrombospondin receptor),CD36 molecule (thrombospondin receptor);SCARB3,GPIV,FAT,GP4,GP3B	CTAGCCAAGGAAAATGTAACCCAGGACGCTGAGGACAACACAGTCTCTTTCCTGCAGCCCAATGGTGCCATCTTCGAACCTTCACTATCAGTTGGAACAG	CD36 molecule
CD38	NM_001775.2	CD38 antigen (p45);ADP-ribosyl cyclase 1,NAD(+) nucleosidase	CTTTCCCGAGACCGTCCTGGCGCGATGCGTCAAGTACACTGAAATTCATCCTGAGATGAGACATGTAGACTGCCAAAGTGTATGGGATGCTTTCAAGGGT	CD38 molecule
CD3D	NM_000732.4	T3D;CD3d antigen, delta polypeptide (TiT3 complex),CD3d molecule, delta (CD3-TCR complex)	CCGCTGGGAGATGGAACATAGCACGTTTCTCTCTGGCCTGGTACTGGCTACCCTTCTCTCGCAAGTGAGCCCCTTCAAGATACCTATAGAGGAACTTGAG	CD3d molecule
CD3E	NM_00	CD3e antigen, epsilon polypeptide (TiT3 complex),CD3e molecule, epsilon (CD3-TCR complex)	AGTAACAGTCCCATGAAACAAAGATGCAGTCGGGCACTCACTGGAGAGTTCTGGGCCTCT	CD3e molecule

	733.3		GCCTCTTATCAGTTGGCGTTTGGGGGCAAGATGGTAATGA		
CD3G	NM_000073.2	CD3g antigen, gamma polypeptide (TiT3 complex),CD3g molecule, gamma (CD3-TCR complex)	GAACTAAATGCAGCCACCATATCTGGCTTTCTCTTTGCTGAAATCGTCAGCATTTTCGTCCTTGCTGTTGGGGTCTACTTCATTGCTGGACAGGATGGAG	CD3g molecule	
CD4	NM_000616.4	CD4 antigen (p55),T-cell surface glycoprotein CD4	CGGCACCGAAGGCGCCAAGCAGAGCGGATGTCTCAGATCAAGAGACTCCTCAGTGAGAAGAAGACCTGCCAGTGTCCTCACCGGTTTCAGAAGACATGTA	CD4 molecule	
CD40	NM_001250.5	TNFRSF5;tumor necrosis factor receptor superfamily, member 5,CD40 molecule, TNF receptor superfamily member 5;p50,Bp50	GCAGAGAAAAACAGTACCTAATAAACAGTCAGTGCTGTTCTTTGTGCCAGCCAGGACAGAAACTGGTGAGTGACTGCACAGAGTTCACTGAAACGGAATG	CD40 molecule	
CD40LG	NM_000074.2	HIGM1,IMD3,TNFSF5;tumor necrosis factor (ligand) superfamily, member 5 (hyper-IgM syndrome);CD40L,TRAP,gp39,hCD40L,CD154;CD40 antigen ligand,tumor necrosis factor (ligand) superfamily member 5,T-B cell-activating molecule,TNF-related activation protein,hyper-IgM syndrome	GCATTTGATTTATCAGTGAAGATGCAGAAGGGAAATGGGGAGCCTCAGCTCACATTCAGTTATGGTTGACTCTGGGTTCCTATGGCCTTGTTGGAGGGGG	CD40 ligand	
CD44	NM_001001392.1	MIC4,MDU2,MDU3;CD44 antigen (homing function and Indian blood group system);IN,MC56,Pgp1,CD44R,HCELL,CSPG8;hematopoietic cell E- and L-selectin ligand,chondroitin sulfate proteoglycan 8	TCTCGGACGGAGGCCGCTGACCTCTGCAAGGCTTTCAATAGCACCTTGCCCACAATGGCCCAGATGGAGAAAGCTCTGAGCATCGGATTTGAGACCTGCA	CD44 molecule (Indian blood group)	
CD45RA	NM_002838.4	MIC4,MDU2,MDU3;CD44 antigen (homing function and Indian blood group system);IN,MC56,Pgp1,CD44R,HCELL,CSPG8;hematopoietic cell E- and L-selectin ligand,chondroitin sulfate proteoglycan 8	TTTCTGGACACAGAAGTATTTGTGACAGGGCAAAGCCCAACACCTTCCCCCACTGGATTGACTACAGCAAAGATGCCCAGTGTTCCACTTTCAAGTGACC		Specific to CD45RA isoform
CD4	ENST	MIC4,MDU2,MDU3;CD44 antigen (homing function and Indian blood group system);IN,MC56,Pgp1,CD44R,HCELL,CSPG8;hemat	TTTCTGGACACAGAAGTATTTGTGACAGGGCAAAGCCCAACACCTTCCCCCACTGGTGTTTCATC		Specific to CD45RB isoform

5RB	000003673667.1	opoietic cell E- and L-selectin ligand,chondroitin sulfate proteoglycan 8	AGTACAGACGCCTCACCTTCCCACGCACGCAGACT		
CD45RO	NM_080921.3	MIC4,MDU2,MDU3;CD44 antigen (homing function and Indian blood group system);IN,MC56,Pgp1,CD44R,HCELL,CSPG8;hematopoietic cell E- and L-selectin ligand,chondroitin sulfate proteoglycan 8	TTTCTGGACACAGAAGTATTTGTGACAGGGCAAAGCCCAACACCTTCCCCCACTGATGCCTACCTTAATGCCTCTGAAACAACCACTCTGAGCCCTTCTG		Specific to CD45RO isoform
CD47	NM_001777.3	MER6;CD47 antigen (Rh-related antigen, integrin-associated signal transducer);IAP,OA3;antigen identified by monoclonal antibody 1D8,antigenic surface determinant protein OA3,integrin associated protein,Rh-related antigen,leukocyte surface antigen CD47,CD47 glycoprotein	TATTGGTTATTCAGGTGATAGCCTATATCCTCGCTGTGGTTGGACTGAGTCTCTGTATTGCGGCGTGTATACCAATGCATGGCCCTCTTCTGATTTCAGG	CD47 molecule	
CD48	NM_001778.3	BCM1;CD48 antigen (B-cell membrane protein),CD48 molecule ;BLAST,mCD48,hCD48,SLAMF2	TGAGGGTGTTGAAAAAGACTGGGAATGAGCAAGAATGGAAGATCAAGCTGCAAGTGCTTGACCCTGTACCCAAGCCTGTCATCAAAATTGAGAAGATAGA	CD48 molecule	
CD5	NM_001346456.1	LEU1;CD5 antigen (p56-62);T1	CGCCAGAAGAAGCAGCGCCAGTGGATTGGCCCAACGGGAATGAACCAAAACATGTCTTTCCATCGCAACCACACGGCAACCGTCCGATCCCATGCTGAGA	CD5 molecule	
CD58	NM_001144822.1	LFA3;CD58 antigen, (lymphocyte function-associated antigen 3)	CTGGCCGACCGCGTAGGCGGTGCTTGAACTTAGGGCTGCTTGTGGCTGGGCACTCGCGCAGAGGCCGGCCCGACGAGCCATGGTTGCTGGGAGCGACGCG	CD58 molecule	

CD6	NM_001254751.1	CD6 antigen;Tp120	AACCCTGGACACTGCATTACAGACCCGCCATCCCTGGGCCCTCAGTATCACCCGAGGAGCAACAGTGAGTCGAGCACCTCTTCAGGGGAGGATTACTGCA	CD6 molecule
CD68	NM_001251.2	CD68 antigen;SCARD1,macrosialin,GP110,DKFZp686M18236,LAMP4;scavenger receptor class D, member 1,CD68 antigen,macrophage antigen CD68	ACCGGTCCATCTTGCTGCCTCTCATCATCGGCCTGATCCTTCTTGGCCTCCTCGCCCTGGTGCTTATTGCTTTCTGCATCATCCGGAGACGCCCATCCGC	CD68 molecule
CD69	NM_001781.2	CD69 antigen (p60, early T-cell activation antigen);CLEC2C	CTGAAAATTGTTTCGTAGCAGAGAACAGCTCTTTGCATCCGGAGAGTGGACAAGAAAATGATGCCACCAGTCCCCATTTCTCAACACGTCATGAAGGGTC	CD69 molecule
CD7	NM_006137.6	CD7 antigen (p41);GP40,LEU-9,TP41,Tp40;p41 protein,T-cell antigen CD7,T-cell leukemia antigen	CCTACACCTGCCAGGCCATCACGGAGGTCAATGTCTACGGCTCCGGCACCCTGGTCCTGGTGACAGAGGAACAGTCCCAAGGATGGCACAGATGCTCGGA	CD7 molecule
CD70	NM_001252.3	CD27LG,TNFSF7;tumor necrosis factor (ligand) superfamily, member 7;CD27L	CTGGACAAGGGGCAGCTACGTATCCATCGTGATGGCATCTACATGGTACACATCCAGGTGACGCTGGCCATCTGCTCCTCCACGACGGCCTCCAGGCACC	CD70 molecule
CD74	NM_001025159.2	DHLAG;CD74 antigen (invariant polypeptide of major histocompatibility complex, class II antigen-associated),CD74 molecule, major histocompatibility complex, class II invariant chain;HLA-DR-gamma,Ia-associated invariant chain,gamma chain of class II antigens,MHC HLA-DR gamma chain	CATCTCCCACCCTGTACCTCATCCCATGAGACCCTGGTGCCTGGCTCTTTCGTCACCCCTTGGACAAGACAAACCAAGTCGGAACAGCAGATAACAATGCA	CD74 molecule
CD7	NM_0	IGA;CD79A antigen (immunoglobulin-associated alpha),CD79a molecule, immunoglobulin-associated alpha;MB-1;B-cell antigen receptor complex-associated protein alpha chain	TCACAGCCGAGGGGATCATCCTCCTGTTCTGCGCGGTGGTGCCTGGGA	CD79a molecule

9A	1783.3		CGCTGCTGCTGTTCAGGAAACGATGGCAGAACGAGAAGCTCGGGTTGGATGC		
CD79B	NM_001039933.1	IGB;CD79B antigen (immunoglobulin-associated beta),CD79b molecule, immunoglobulin-associated beta;B29;B-cell antigen receptor complex-associated protein beta chain	TGAAGCTGGCCCACCAGAGCTGCCATTTGTCTCCAGCCCCTGGTCCCCAGCTCTTGCCAAAGGGCCTGGAGTAGAAGGACAACAGGGCAGCAACTTGGAG	CD79b molecule	
CD80	NM_005191.3	CD28LG,CD28LG1;CD80 antigen (CD28 antigen ligand 1, B7-1 antigen),CD80 molecule ;B7.1,B7-1;B-lymphocyte activation antigen B7	GATATCACTAATAACCTCTCCATTGTGATCCTGGCTCTGCGCCCATCTGACGAGGGCACATACGAGTGTGTTGTTCTGAAGTATGAAAAAGACGCTTTCA	CD80 molecule	
CD84	NM_001184879.1	CD84 antigen (leukocyte antigen),CD84 molecule ;SLAMF5,hCD84,mCD84	AACACAGTTTATTCCGAAGTGCAGTTTGCTGATAAGATGGGGAAAGCCAGCACACAGGACAGTAAACCTCCTGGGACTTCAAGCTATGAAATTGTGATCT	CD84 molecule	
CD86	NM_001206924.1	CD28LG2;CD86 antigen (CD28 antigen ligand 2, B7-2 antigen);B7.2,B7-2;B-lymphocyte antigen B7-2	CATGAAATGTCTGGTCTGTCCACCCCATCAACAAGTCTTGAAACAAGCAACAGATGGATAGTCTGTCCAAATGGACATAAGACAGACAGCAGTTTCCCTG	CD86 molecule	
CD8A	NM_001768.6	CD8;CD8 antigen, alpha polypeptide (p32),T-cell surface glycoprotein CD8 alpha chain	AGCTCAGGGCTCTTTCCTCCACACCATTCAGGTCTTTCTTTCCGAGGCCCCTGTCTCAGGGTGAGGTGCTTGAGTCTCCAACGGCAAGGGAACAAGTACT	CD8a molecule	
CD8B	NM_172	CD8B1;CD8 antigen, beta polypeptide 1 (p37)	GGAGAAGATAGCTGTGTTTCGGGATGCAAGCCGGTTCATTCTCAATCTCACAAGCGTGAA	CD8b molecule	CD8B2 (NM_001349727;NM_001368307)

	213.2		GCCGGAAGACAGTGGCATCTACTTCTGCATGATCGTCGGG	
CD96	NM_005816.4	CD96 antigen;TACTILE	CCAGCTACATCTTCAGTGACCCTTGTAGATGTGAGTGCCTTGAGGCCAAACACCACTCCTCAACCCAGCAATTCCAGTATGACTACCCGAGGCTTCAACT	CD96 molecule
CDC20	NM_001255.1	CDC20 (cell division cycle 20, S. cerevisiae, homolog),CDC20 cell division cycle 20 homolog (S. cerevisiae),cell division cycle 20 homolog (S. cerevisiae);p55CDC,CDC20A	CCCGAGTGGGCTCCCTAAGCTGGAACAGCTATATCCTGTCCAGTGGTTCACGTTCTGGCCACATCCACCACCATGATGTTCGGGTAGCAGAACACCATGT	cell division cycle 20
CDC25C	NM_001790.4	CDC25;CDC25;PPP1R60;protein phosphatase 1, regulatory subunit 60	TTTTCTGGCCAAGGAAAGCTCAGGAAGGGCTTATGTTTAAAGAAGACAGTCTCTCTGTGTGACATTACTATCACTCAGATGCTGGAGGAAGATTCTAACC	cell division cycle 25C
CDH1	NM_004360.3	UVO;cadherin 1, type 1, E-cadherin (epithelial);uvomorulin,CD324;E-Cadherin	AAAAGGCCTCTACGGTTTCATAACCCACAGATCCATTTCTTGGTCTACGCCTGGGACTCCACCTACAGAAAGTTTTCCACCAAAGTCACGCTGAATACAG	cadherin 1
CDH11	NM_001797.2	cadherin 11, type 2, OB-cadherin (osteoblast);OB,CAD11;OB-Cadherin	CAGCTCAGCGGCCCTGTGACATTCCTTCGTGTTGTCATTTGTTGAGTGACCAATCAGATGGGTGGAGTGTGTTACAGAAATTGGCAGCAAGTATCCAATG	cadherin 11
CDH2	NM_001792.3	NCAD;cadherin 2, type 1, N-cadherin (neuronal);CDHN,CD325;N-cadherin	TGAGCTCCCTTAATTCCTCAAGTAGTGGTGGTGAGCAGGACTATGATTACCTGAACGACTGGGGGCCACGGTTCAAGAAACTTGCTGACATGTATGGTGG	cadherin 2
CDH5	NM_0017	cadherin 5, type 2, VE-cadherin (vascular epithelium),cadherin 5, type 2 (vascular endothelium);7B4,CD144;VE-cadherin	TCTCCCCTTCTCTGCCTCACCTGGTCGCCAATCCATGCTCTCTTTCTTTTCTCTGTCTACTCCTTATCCCTTGGTT	cadherin 5

	95.3		TAGAGGAACCCAAGATGTGGCCTT		
CDK2	NM_001290230.1		AATGATTGGCCCCAGTCCCCTTGTTTGTCCCTTCTACAGGCATGAGGAATCTGGGAGGCCCTGAGACAGGGATTGTGCTTCATTCCAATCTATTGCTTCA	cyclin dependent kinase 2	
CDK6	NM_001145306.1	PLSTIRE	CCAAAAGAATATCTGCCTACAGTGCCCTGTCTCACCCATACTTCCAGGACCTGGAAAGGTGCAAAGAAAACCTGGATTCCCACCTGCCGCCCAGCCAGAA	cyclin dependent kinase 6	
CDKN1A	NM_000389.2	CDKN1;cyclin-dependent kinase inhibitor 1A (p21, Cip1);P21,CIP1,WAF1,SDI1,CAP20,p21CIP1,p21Cip1/Waf1,p21	CATGTGTCCTGGTTCCCGTTTCTCCACCTAGACTGTAAACCTCTCGAGGGCAGGGACCACACCCTGTACTGTTCTGTGTCTTTCACAGCTCCTCCCACAA	cyclin dependent kinase inhibitor 1A	Sub-maximal isoform coverage due to non-overlapping transcripts
CDKN1C	NM_000076.2	BWCR,BWS;Beckwith-Wiedemann syndrome,cyclin-dependent kinase inhibitor 1C (p57, Kip2);P57,KIP2	TGTCCAGGCGTGGACCGCTCTGCCACGCACTAGCTCGGTTATTGGTTATGCCAAAGGCACTCTCCATCTCCCACATCTGGTTATTGACAAGTGTAACTTT	cyclin dependent kinase inhibitor 1C	
CDKN2A	NM_000077.4	CDKN2,MLM;cyclin-dependent kinase inhibitor 2A (melanoma, p16, inhibits CDK4);CDK4I,p16,INK4a,MTS1,CMM2,ARF,p19,p14,INK4,p16INK4a,p19Arf,p14ARF	GTGAGCACTCACGCCCTAAGCGCACATTCATGTGGGCATTTCTTGCGAGCCTCGCAGCCTCCGGAAGCTGTCGACTTCATGACAAGCATTTTGTGAACTA	cyclin dependent kinase inhibitor 2A	
CDKN2B	NM_004936.3	cyclin-dependent kinase inhibitor 2B (p15, inhibits CDK4);P15,MTS2,INK4B,TP15,CDK4I,p15INK4b	ACTCACAGGAAGGAGGAGCCGACCGGGAATAACCTTCCATACATTTTTTTCTTTGTCTTATCTGGCCCTCGACACTCACCATGAAGCGAAACACAGAGAA	cyclin dependent kinase inhibitor 2B	

CEACAM3	NM_001277163.2	CGM1;carcinoembryonic antigen-related cell adhesion molecule 3,carcinoembryonic antigen related cell adhesion molecule 3;CD66d	GGAAGGACATGAAGCCTGAGCCAGAGAACCAGCTATAAGTCCTGAGAAGACACTGGTGTCTGGGGGCAGGGAGGGATGGGGGTCCCTGATGAATATCTGG	CEA cell adhesion molecule 3
CEBPB	NM_005194.3	TCF5;CCAAT/enhancer binding protein (C/EBP), beta,CCAAT/enhancer binding protein beta;LAP,CRP2,NFIL6,IL6DBP,C/EBP-beta;liver-enriched transcriptional activator protein,nuclear factor of interleukin 6,interleukin 6-dependent DNA-binding protein	CTATGTGTACAGATGAATGATAAACTCTCTGCTTCTCCCTCTGCCCCTCTCCAGGCGCCGGCGGGCGGGCCGGTTTCGAAGTTGATGCAATCGGTTTAAA	CCAAT enhancer binding protein beta
CENPF	NM_016343.3	centromere protein F, 350/400kDa (mitosin),centromere protein F, 350/400kDa;hcp-1;mitosin	AGAAAATCTTGCAGAGTCCTCCAAACCAACAGCTGGTGGCAGCAGATCACAAAAGGTCAAAGTTGCTCAGCGGAGCCCAGTAGATTCAGGCACCATCCTC	centromere protein F
CEP55	NM_018131.3	C10orf3;chromosome 10 open reading frame 3,centrosomal protein 55kDa;FLJ10540,CT111;cancer/testis antigen 111	GTACTACCGCATTGCTTGAACAGCTGGAAGAGACAACGAGAGAAGGAGAAAGGAGGGAGCAGGTGTTGAAAGCCTTATCTGAAGAGAAAGACGTATTGAA	centrosomal protein 55
CES3	NM_001185176.1	carboxylesterase 3 (brain);FLJ21736,ES31;esterase 31,brain carboxylesterase BR3	CCAATTCAACCAGGCGGAACAATATCTGGAGATCAACCCAGTGCCACGGGCCGGACAGAAGTTCAGGGAGGCCTGGATGCAGTTCTGGTCAGAGACGCTC	carboxylesterase 3
CHUK	NM_001278.4	TCF16;conserved helix-loop-helix ubiquitous kinase;IKK1,IKK-alpha,IkBKA,NFKBIKA,IKKA;inhibitor of nuclear factor kappa-B kinase subunit alpha,I-kappa-B kinase	TGTGAATATTCCTTTATTTTGCTGCTTGATGATGAGAGGGAGGGCTGCTGCCACAGACTGTGGTGAGGGCTGGTTAATGTAGTATGGTATATGCACAAAA	component of inhibitor of nuclear factor kappa B kinase complex
CLEC	NM_17	C14orf27;chromosome 14 open reading frame 27,C-type lectin domain family 14, member A,C-type lectin domain family 14 member A	CTCTAAATTCCCTTTACTCCACTGAGGAGCTAAATCAGAACTGCACACT	C-type lectin domain containing 14A

14A	5060.1		CCTTCCCTGATGATAGAGGAAGTGGAAGTGCCTTTAGGATGGTGATACTGG	
CLEC4E	NM_014358.3	CLECSF9;C-type (calcium dependent, carbohydrate-recognition domain) lectin, superfamily member 9,C-type lectin domain family 4, member E;mincle;Macrophage-inducible C-type lectin	AGGGAAGGGCATGCCAGCCATCAGCTCCAAACAGGCTGTAACCAAGTCCACCCATCCCTGGGGCTTCCTTTGCTCTGCCTTATTTTCAATTGACTGAATG	C-type lectin domain family 4 member E
CLEC5A	NM_013252.2	CLECSF5;C-type (calcium dependent, carbohydrate-recognition domain) lectin, superfamily member 5,C-type lectin domain family 5, member A,C-type lectin domain family 5 member A;MDL-1	GGCGTTGGATCAACAACTCTGTGTTCAATGGCAATGTTACCAATCAGAATCAGAATTTCAACTGTGCGACCATTGGCCTAACAAAGACATTTGATGCTGC	C-type lectin domain containing 5A
CLEC7A	NM_197954.2	CLECSF12;C-type (calcium dependent, carbohydrate-recognition domain) lectin, superfamily member 12,C-type lectin domain family 7, member A,C-type lectin domain family 7 member A;dectin-1,hDectin-1,CD369,SCARE2	TGTTAAACTCCGGTAAGTACCTAGCCCACATGATTTGACTCAGAGATTCTCTTTTGTCCACAGACAGTCATCTCAGGAGCAGAAAGAAAAGAGCTCCCAA	C-type lectin domain containing 7A
CLECL1	NM_172004.3	C-type lectin-like 1;DCAL1;dendritic cell associated lectin 1	TCAACTTTTCTACTGTCCATAAATCATGTCCTGCCAAAGACTGGAAGGTGCATAAGGGAAAATGTTACTGGATTGCTGAAACTAAGAAATCTTGGAACAA	C-type lectin like 1
CMKLR1	NM_004072.2	chemokine-like receptor 1;RVER1;resolvin E1 receptor,chemerin receptor	TTCAACGTCTTCCTCCCAATCCATATCACCTATGCCGCCATGGACTACCACTGGGTTTTCGGGACAGCCATGTGCAAGATCAGCAACTTCCTTCTCATCC	chemerin chemokine-like receptor 1
CNTFR	NM_147164.2		GATTGGGACATGGAGTGACTGGAGCGTAGCCGCCCACGCTACGCCCTGGACTGAGGAACCGCGACACCTCACCACGGAGGCCCAGGCTGCGGAGACCACG	ciliary neurotrophic factor receptor
COL11	NM_001	COLL6,DFNA37;collagen, type XI, alpha 1,deafness, autosomal dominant 37;STL2,CO11A1;collagen XI, alpha-1 polypeptide	AGACAAAGAACATATCAAATCAACAGAAAATATACCTTGGTGCCACCAACCCATTTTGTG	collagen type XI alpha 1 chain

A1	854.3		CCACATGCAAGTTTTGAATAAGGATGGTATAGAAAACAAC	
COL11A2	NM_001163771.1	DFNA13,DFNB53;collagen, type XI, alpha 2;HKE5	CAAGCTCCCCTCCTGACTCTCTACAGTGCCCAGGGTGTCCGACAGCTGGGCCTGGAGCTGGGCCGACCTGTCCGCTTCCTGTATGAAGACCAGACTGGGC	collagen type XI alpha 2 chain
COL17A1	NM_000494.3	BPAG2;collagen, type XVII, alpha 1;BP180	TTTCGACTACTCAGAGCTGGCAAGCCACGTTGTGAGCTACTTACGGACTTCGGGGTACGGTGTCAGCTTGTTCTCGTCCTCCATCTCTTCTGAAGACATT	collagen type XVII alpha 1 chain
COL4A5	NM_000495.4	ASLN,ATS;Alport syndrome,collagen, type IV, alpha 5,collagen type IV alpha 5	GTGACACTTGCTTCAACTGCATTGGAACTGGTATTTCAGGGCCTCCAGGTCAACCTGGTTTGCCAGGTCTCCCAGGTCCTCCAGGATCTCTTGGTTTCCC	collagen type IV alpha 5 chain
COL5A1	NM_000093.4	collagen type V alpha 1;alpha 1 type V collagen	ACCCCACAGTCGCAGGACCCCAATCCAGATGAATATTACACGGAAGGAGACGGCGAGGGTGAGACCTATTACTACGAATACCCCTACTACGAAGACCCCG	collagen type V alpha 1 chain
COL6A3	NM_004369.3	collagen, type VI, alpha 3	AGATCTTCATCCACAGATAGTGAATCTCTTAAAATCAGTGCACAACGGAGCACCAGCACCAGTTTCAGGTGAAAAGGACGTGGTGTTTCTGCTTGATGGC	collagen type VI alpha 3 chain
COMP	NM_000095.2	PSACH,EDM1,EPD1;cartilage oligomeric matrix protein (pseudoachondroplasia, epiphyseal dysplasia 1, multiple);MED,THBS5;thrombospondin-5	GAAGAGCAACCCGGATCAGGCGGATGTGGACCACGACTTTGTGGGAGATGCTTGTGACAGCGATCAAGACCAGGATGGAGACGGACATCAGGACTCTCGG	cartilage oligomeric matrix protein
CP	NM_0	carboxypeptidase A3 (mast cell);mast cell carboxypeptidase A,tissue carboxypeptidase A	GAACCAAAACTCCAAATGCATCGGCACTGACCTCAA	carboxypeptidase A3

A3	01870.2		CAGGAATTTTAATGCTTCATGGAACTCCATTCCTAACACCAATGACCCATGTGCAGATAACTAT	
CRABP2	NM_001199723.1	cellular retinoic acid-binding protein 2;CRABP-II	TCCCCTACACCAACAAAGAGGAATGGCTGCAAGAGCCCAGATCACCCATTCCGGGTTCACTCCCCGCCTCCCCAAGTCAGCAGTCCTAGCCCCAAACCAG	cellular retinoic acid binding protein 2
CSF1	NM_000757.5	colony stimulating factor 1 (macrophage);M-CSF,MCSF,MGC31930;macrophage colony stimulating factor 1	GGGAAAGTGAAAGTTTGCCTGGGTCCTCTCGGCGCCAGAGCCGCTCTCCGCATCCCAGGACAGCGGTGCGGCCCTCGGCCGGGGCGCCCACTCCGCAGCA	colony stimulating factor 1
CSF1R	NM_005211.3	FMS;McDonough feline sarcoma viral (v-fms) oncogene homolog;C-FMS,CSFR,CD115	GTGTGCTCAGCCAGCAGCGTTGATGTTAACTTTGATGTCTTCCTCCAACACAACAACACCAAGCTCGCAATCCCTCAACAATCTGACTTTCATAATAACC	colony stimulating factor 1 receptor
CSF2	NM_000758.3	colony stimulating factor 2 (granulocyte-macrophage);GM-CSF,GMCSF;sargramostim,molgramostim,granulocyte-macrophage colony stimulating factor	GCACGCAGCCCTGGGAGCATGTGAATGCCATCCAGGAGGCCCGGCGTCTCCTGAACCTGAGTAGAGACACTGCTGCTGAGATGAATGAAACAGTAGAAGT	colony stimulating factor 2
CSF2RB	NM_000395.2	IL3RB;colony stimulating factor 2 receptor, beta, low-affinity (granulocyte-macrophage),colony stimulating factor 2 receptor beta common subunit;IL5RB,CD131,betaGMR;beta common cytokine receptor,beta-GM-CSF receptor	TGCTGGCCCTCATCGTGATCTTCCTCACCATCGCTGTGCTCCTGGCCCTCCGCTTCTGTGGCATCTACGGGTACAGGCTGCGCAGAAAGTGGGAGGAGAA	colony stimulating factor 2 receptor subunit beta
CSF3	NM_000759.3	GCSF,G-CSF,C17orf33;chromosome 17 open reading frame 33,colony stimulating factor 3 (granulocyte);MGC45931;granulocyte colony stimulating factor,pluripoietin,filgrastim,lenograstim	CCTGCATTTCTGAGTTTCATTCTCCTGCCTGTAGCAGTGAGAAAAAGCTCCTGTCCTCCCATCCCCTGGACTGGGAGGTAGATAGGTAAATACCAAGTAT	colony stimulating factor 3

CSF3R	NM_000760.3	CD114;colony stimulating factor 3 receptor (granulocyte);GCSFR	AGGCCCTTTCAGCTCTATGAGATCATCGTGACTCCCTTGTACCAGGACACCATGGGACCCTCCCAGCATGTCTATGCCTACTCTCAAGAAATGGCTCCCT	colony stimulating factor 3 receptor	
CST2	NM_001322.2	cystatin 2	TACTACAGACGCCTGCTGCGGGTGCTACGAGCCAGGGAGCAGATCGTGGGCGGGGTGAATTACTTCTTCGACATAGAGGTGGGCCGAACCATATGTACCA	cystatin SA	
CTAG1B	NM_001327.2	CTAG,CTAG1;cancer/testis antigen 1;NY-ESO-1,LAGE2B,LAGE2A,ESO1,CT6.1	GCGGGGCCAGGGGGCCGGAGAGCCGCCTGCTTGAGTTCTACCTCGCCATGCCTTTCGCGACACCCATGGAAGCAGAGCTGGCCCGCAGGAGCCTGGCCCA	cancer/testis antigen 1B	CTAG1A (NM_139250)
CTLA4	NM_001037631.2	CELIAC3,IDDM12;celiac disease 3,insulin-dependent diabetes mellitus 12;CD152,CD,GSE,CTLA-4	CAGGTGACTGAAGTCTGTGCGGCAACCTACATGATGGGGAATGAGTTGACCTTCCTAGATGATTCCATCTGCACGGGCACCTCCAGTGGAAATCAAGTGA	cytotoxic T-lymphocyte associated protein 4	
CTNNB1	NM_001098209.1	CTNNB;catenin (cadherin-associated protein), beta 1 (88kD),catenin (cadherin-associated protein), beta 1, 88kDa,catenin (cadherin-associated protein), beta 1;beta-catenin,armadillo	GCAGCAATTTGTGGAGGGGGTCCGCATGGAAGAAATAGTTGAAGGTTGTACCGGAGCCCTTCACATCCTAGCTCGGGATGTTCACAACCGAATTGTTATC	catenin beta 1	
CTSS	NM_004079.3		ATGACAACGGCTTTCCAGTACATCATTGATAACAAGGGCATCGACTCAGACGCTTCCTATCCCTACAAAGCCATGGATCAGAAATGTCAATATGACTCAA	cathepsin S	
CTSW	NM_0	cathepsin W (lymphopain)	TGCACCGAGGGAGCAATACCTGTGGCATCACCAAGTTCCCGCTCACTG	cathepsin W	

	1335.3		CCCGTGTGCAGAAACCGGATATGAAGCCCCGAGTCTCCTGCCCTCCCTGAAC	
CX3CL1	NM_002996.3	SCYD1;small inducible cytokine subfamily D (Cys-X3-Cys), member 1 (fractalkine, neurotactin),chemokine (C-X3-C motif) ligand 1;NTN,C3Xkine,ABCD-3,CXC3C,CXC3;fractalkine,neurotactin	ATTGTGGGAAGGGGAGATAAGGGTATCTGGTGACTTTCCTCTTTGGTCTACACTGTGCTGAGTCTGAAGGCTGGGTTCTGATCCTAGTTCCACCATCAAG	C-X3-C motif chemokine ligand 1
CX3CR1	NM_001171171.1	GPR13,CMKBRL1;chemokine (C-X3-C) receptor 1,chemokine (C-X3-C motif) receptor 1;CMKDR1,V28,CCRL1	GATAGCCTTCCGTAACCCAACTCTCCTGGACTGCCTTGAATATCCCCTCCCAGTCACCTTGTGGCAAGCCCCTGCCCATCTGGGAAAATACCCCATCATT	C-X3-C motif chemokine receptor 1
CXCL1	NM_001511.3	MGSA,GRO1,FSP;GRO1 oncogene (melanoma growth stimulating activity, alpha),fibroblast secretory protein,chemokine (C-X-C motif) ligand 1 (melanoma growth stimulating activity, alpha);SCYB1,GROa,MGSA-a,NAP-3;melanoma growth stimulating activity, alpha	ATGTTAATATTTCTGAGGAGCCTGCAACATGCCAGCCACTGTGATAGAGGCTGGCGGATCCAAGCAAATGGCCAATGAGATCATTGTGAAGGCAGGGGAA	C-X-C motif chemokine ligand 1
CXCL10	NM_001565.3	INP10,SCYB10;small inducible cytokine subfamily B (Cys-X-Cys), member 10,chemokine (C-X-C motif) ligand 10;IFI10,IP-10,crg-2,mob-1,C7,gIP-10	TCTGATTTGCTGCCTTATCTTTCTGACTCTAAGTGGCATTCAAGGAGTACCTCTCTCTAGAACTGTACGCTGTACCTGCATCAGCATTAGTAATCAACCT	C-X-C motif chemokine ligand 10
CXCL11	NM_005409.4	SCYB9B,SCYB11;small inducible cytokine subfamily B (Cys-X-Cys), member 11,chemokine (C-X-C motif) ligand 11;H174,b-R1,I-TAC,IP-9	GTGGCAGATATTGAGAAAGCCTCCATAATGTACCCAAGTAACAACTGTGACAAAATAGAAGTGATTATTACCCTGAAAGAAAATAAAGGACAACGATGCC	C-X-C motif chemokine ligand 11
CXCL12	NM_199168.3	SDF1A,SDF1B,SDF1;stromal cell-derived factor 1,chemokine (C-X-C motif) ligand 12;SCYB12,SDF-1a,SDF-1b,PBSF,TLSF-a,TLSF-b,TPAR1	CCCGCCCGCCCGCCCGCGCCATGAACGCCAAGGTCGTGGTCGTGCTGGTCCTCGTGCTGACCGCGCTCTGCCTCAGCGACGGGAAGCCCGTCAGCCTGAG	C-X-C motif chemokine ligand 12
CX	NM	SCYB13;small inducible cytokine B subfamily (Cys-X-Cys motif), member 13 (B-cell	TAGACGCTTCATTGATCGAATTCA	C-X-C motif chemokine ligand 13

CL13	_006419.2	chemoattractant),chemokine (C-X-C motif) ligand 13;BLC,BCA-1,BLR1L,ANGIE,ANGIE2;B-cell chemoattractant	AATCTTGCCCCGTGGGAATGGTTGTCCAAGAAAAGAAATCATAGTCTGGAAGAAGAACAAGTCAATTGTGTGTGTG	
CXCL14	NM_004887.4	SCYB14;small inducible cytokine subfamily B (Cys-X-Cys), member 14 (BRAK),chemokine (C-X-C motif) ligand 14;BRAK,NJAC,bolekine,Kec,MIP-2g,BMAC,KS1;breast and kidney	TTATAGCTGCGTGCGAAAGGCTTCCAGATGGGAGACCCATCTCTCTTGTGCTCCAGACTTCATCACAGGCTGCTTTTTATCAAAAAGGGGAAAACTCATG	C-X-C motif chemokine ligand 14
CXCL16	NM_001100812.1	chemokine (C-X-C motif) ligand 16;SR-PSOX,CXCLG16,SRPSOX;CXC chemokine ligand 16	TTCAGTTCATGAATCGTCTCCGGAAACACCTGAGAGCTTACCATCGGTGTCTATACTACACGAGGTTCCAGCTCCTTTCCTGGAGCGTGTGTGGGGGCAA	C-X-C motif chemokine ligand 16
CXCL2	NM_002089.1	GRO2;GRO2 oncogene,chemokine (C-X-C motif) ligand 2;SCYB2,GROb,MIP-2a,MGSA-b,CINC-2a	GAAGGAGGCCCTGCCTTACAGGAACAGAAGAGGAAAGAGAGACACAGCTGCAGAGGCCACCTGGCTTGCGCCTAATGTGTTTGAGCATACTTAGGAGAAG	C-X-C motif chemokine ligand 2
CXCL3	NM_002090.2	GRO3;GRO3 oncogene,chemokine (C-X-C motif) ligand 3;SCYB3,GROg,MIP-2b,CINC-2b	CAAGGGGAGCACCAACTGACAGGAGAGAAGTAAGAAGCTTATCAGCGTATCATTGACACTTCCTGCAGGGTGGTCCCTGCCCTTACCAGAGCTGAAAATG	C-X-C motif chemokine ligand 3
CXCL5	NM_002994.4	SCYB5;small inducible cytokine subfamily B (Cys-X-Cys), member 5 (epithelial-derived neutrophil-activating peptide 78),chemokine (C-X-C motif) ligand 5;ENA-78	GGAAACAAGGAAAACTGATTAAGAGAAATGAGCACGCATGGAAAAGTTTCCCAGTCTTCAGCAGAGAAGTTTTCTGGAGGTCTCTGAACCCAGGGAAGAC	C-X-C motif chemokine ligand 5
CXCL6	NM_002993.3	SCYB6;small inducible cytokine subfamily B (Cys-X-Cys), member 6 (granulocyte chemotactic protein 2),chemokine (C-X-C motif) ligand 6 (granulocyte chemotactic protein 2),chemokine (C-X-C motif) ligand 6;GCP-2,CKA-3;granulocyte chemotactic protein 2	TCCTGTCTCTGCTGTGCTGACAGAGCTGCGTTGCACTTGTTTACGCGTTACGCTGAGAGTAAACCCCAAAACGATTGGTAAACTGCAGGTGTTCCCCGCA	C-X-C motif chemokine ligand 6

CXCL8	NM_000584.3	IL8;interleukin 8,chemokine (C-X-C motif) ligand 8;SCYB8,LUCT,LECT,MDNCF,TSG-1,IL-8,NAP-1,3-10C,MONAP,AMCF-I,LYNAP,NAF,b-ENAP,GCP-1,K60,GCP1,NAP1;neutrophil-activating peptide 1,granulocyte chemotactic protein 1,monocyte-derived neutrophil chemotactic factor,lung giant cell carcinoma-derived chemotactic protein,tumor necrosis factor-induced gene 1,monocyte-derived neutrophil-activating peptide,lymphocyte derived neutrophil activating peptide,beta endothelial cell-derived neutrophil activating peptide,alveolar macrophage chemotactic factor I	CGTGGCTCTCTTGGCAGCCTTCCTGATTTCTGCAGCTCTGTGTGAAGGTGCAGTTTTGCCAAGGAGTGCTAAAGAACTTAGATGTCAGTGCATAAAGACA	C-X-C motif chemokine ligand 8
CXCL9	NM_002416.2	CMK,MIG;monokine induced by gamma interferon,chemokine (C-X-C motif) ligand 9;SCYB9,Humig,crg-10	CCATCTCCCATGAAGAAAGGGAACGGTGAAGTACTAAGCGCTAGAGGAAGCAGCCAAGTCGGTTAGTGGAAGCATGATTGGTGCCCAGTTAGCCTCTGCA	C-X-C motif chemokine ligand 9
CXCR2	NM_001557.3	IL8RB;interleukin 8 receptor, beta,chemokine (C-X-C motif) receptor 2;CMKAR2,CD182	ACCTCAAAAATGGAAGATTTTAACATGGAGAGTGACAGCTTTGAAGATTTCTGGAAAGGTGAAGATCTTAGTAATTACAGTTACAGCTCTACCCTGCCCC	C-X-C motif chemokine receptor 2
CXCR3	NM_001142797.1	GPR9;G protein-coupled receptor 9,chemokine (C-X-C motif) receptor 3;CKR-L2,CMKAR3,IP10-R,MigR,CD183	GTCCAGTGGGTCTTTGGCTCTGGCCTCTGCAAAGTGGCAGGTGCCCTCTTCAACATCAACTTCTACGCAGGAGCCCTCCTGCTGGCCTGCATCAGCTTTG	C-X-C motif chemokine receptor 3
CXCR4	NM_001008540.1	chemokine (C-X-C motif), receptor 4 (fusin),chemokine (C-X-C motif) receptor 4;LESTR,NPY3R,HM89,NPYY3R,D2S201E,fusin,HSY3RR,NPYR,CD184	TTCGCCTGTTGGCTGCCTTACTACATTGGGATCAGCATCGACTCCTTCATCCTCCTGGAAATCATCAAGCAAGGGTGTGAGTTTGAGAACACTGTGCACA	C-X-C motif chemokine receptor 4
CXCR6	NM_006564.1	chemokine (C-X-C motif) receptor 6;TYMSTR,STRL33,BONZO,CD186	ACCATGAAGACTATGGGTTCAGCAGTTTCAATGACAGCAGCCAGGAGGAGCATCAAGACTTCCTGCAGTTCAGCAAGGTCTTTCTGCCCTGCATGTACCT	C-X-C motif chemokine receptor 6

CXorf36	NM_024689.2	CXorf36;chromosome X open reading frame 36;FLJ14103,DIA1R	GCTGCTGTGGGTCTCAGCCCTGAGCTGTTCTTTCTCCTTGCCAGCTTCTTCCCTTTCTTCTCTGGTGCCCCAAGTCAGAACCAGCTACAATTTTGGAAGG	divergent protein kinase domain 2B
CYBB	NM_000397.3	CGD;chronic granulomatous disease,cytochrome b-245, beta polypeptide;GP91-PHOX,NOX2;NADPH oxidase 2	TTTTTGTCAAGTGCCCAAAGGTGTCCAAGCTGGAGTGGCACCCTTTTACACTGACATCCGCCCCTGAGGAAGACTTCTTTAGTATCCATATCCGCATCGT	cytochrome b-245 beta chain
DAB2	NM_001244871.1	disabled (Drosophila) homolog 2 (mitogen-responsive phosphoprotein),disabled homolog 2, mitogen-responsive phosphoprotein (Drosophila),Dab, mitogen-responsive phosphoprotein, homolog 2 (Drosophila),DAB2, clathrin adaptor protein;DOC-2	AAGGGAGAGCAGACTTCTTCTGGGACTTTGAGTGCCTTTGCCAGTTATTTCAACAGCAAGGTTGGCATTCCTCAGGAGAATGCAGACCATGATGACTTTG	DAB adaptor protein 2
DDB2	NM_000107.2	damage-specific DNA binding protein 2 (48kD);DDBB,UV-DDB2,FLJ34321,XPE;xeroderma pigmentosum group E protein,UV-damaged DNA-binding protein 2,DDB p48 subunit	GATTTGTTAAAGGGCCAAAAGTATCCAAGGTTAGGGTTGGAGCAGGGGTGCTGGGACCTGGGGCACTGTGGGACTGGGACACTTTTATGTTAATGCTCTG	damage specific DNA binding protein 2
DEFB134	NM_001302695.1	defensin, beta 134	AAAAATGGCATCTGCAGACTTGAATGCTATGAGAGTGAAATGTTAGTTGCCTACTGTATGTTTCAGCTGGAGTGCTGTGTCAAAGGAAATCCTGCACCCT	defensin beta 134
DEPTOR	NM_001283012.1	DEPDC6;DEP domain containing 6;DEP.6,FLJ12428	CAGTGGCCTGTGGGTGAGGGAAGCCAGAATGACACAAAGCAATGCAAAGACAAGATTGCCATGCAAATGGATGGTTTTGGACATACGAGTCTTCTCCGCA	DEP domain containing MTOR interacting protein

DKK1	NM_012242.2	dickkopf (Xenopus laevis) homolog 1,dickkopf 1 homolog (Xenopus laevis);SK,DKK-1	CGCTATGTGCTGCCCCGGGAATTACTGCAAAAATGGAATATGTGTGTCTTCTGATCAAAATCATTTCCGAGGAGAAATTGAGGAAACCATCACTGAAAGC	dickkopf WNT signaling pathway inhibitor 1
DLL1	NM_005618.3	delta (Drosophila)-like 1,delta-like 1 (Drosophila)	TCCTGTACCTGCCCACCCGGCTTCTACGGCAAAATCTGTGAATTGAGTGCCATGACCTGTGCGGACGGCCCTTGCTTTAACGGGGGTCGGTGCTCAGACA	delta like canonical Notch ligand 1
DLL4	NM_019074.2	delta-like 4 homolog (Drosophila),delta-like 4 (Drosophila)	AATGACCACTTCGGCCACTATGTGTGCCAGCCAGATGGCAACTTGTCCTGCCTGCCCGGTTGGACTGGGGAATATTGCCAACAGCCTATCTGTCTTTCGG	delta like canonical Notch ligand 4
DNMT1	NM_001130823.2	DNMT;DNA (cytosine-5-)-methyltransferase 1;MCMT,CXXC9	GACTGATGGGAAGAAGAGTTACTATAAGAAGGTGTGCATTGATGCGGAAACCCTGGAAGTGGGGGACTGTGTCTCTGTTATTCCAGATGATTCCTCAAAA	DNA methyltransferase 1
DPP4	NM_001935.3	CD26,ADCP2;dipeptidylpeptidase IV (CD26, adenosine deaminase complexing protein 2),adenosine deaminase complexing protein 2,dipeptidyl-peptidase 4;DPPIV	TCCAACTTAGTGACTATACAAAAGTGACATGCCTCAGTTGTGAGCTGAATCCGGAAAGGTGTCAGTACTATTCTGTGTCATTCAGTAAAGAGGCGAAGTA	dipeptidyl peptidase 4
DTX3L	NM_138287.3	deltex 3-like (Drosophila);BBAP,RNF143;rhysin 2	AAATCAAGAGTCAATGACTTTGACTGGTTTGCCAAATCACCTTGCAAAGGCGAAGCAGTATGTTCTAAAAGGAGGAGGAATGTCTTCATTGGCTGGAAAG	deltex E3 ubiquitin ligase 3L
DTX4	NM_01517	deltex homolog 4 (Drosophila);KIAA0937,RNF155	GCATCTACTACCTTGACACAGAGTGTTTTCCCACTAGAAGCTCTGCTCTGCTCTCCTGGCCCAAGTAGGGGATTCCATGCCTTCCCT	deltex E3 ubiquitin ligase 4

	7.1		TTCATGGTCTTAG	
DUSP1	NM_004417.3	PTPN10;HVH1,CL100,MKP-1	GGAGGGGCTCGAGAGGGCTGGTCCTTATTTATTTAACTTCACCCGAGTTCCTCTGGGTTTCTAAGCAGTTATGGTGATGACTTAGCGTCAAGACATTTGC	dual specificity phosphatase 1
DUSP2	NM_004418.3	PAC-1	CTGGCCCTCATTCGGGGTCGGGAACCAAGGGTGTGTCTGCTCTTTCCCTCCCCATCCTCTGGCAGAAATCAGCTAGACGCTATACCGTGGACTCTCCCTG	dual specificity phosphatase 2
DUSP5	NM_004419.3	HVH3	GTGGATGTAAAACCCATTTCACAAGAGAAGATTGAGAGTGAGAGAGCCCTCATCAGCCAGTGTGGAAAACCAGTGGTAAATGTCAGCTACAGGCCAGCTT	dual specificity phosphatase 5
E2F3	NM_001243076.2		TTGGATGCTAAGGACTGCGGGAATGAGGGAGTCAGATAAAGAACAAACCTCGAAACGAACAGTTAAATTGAAATGCTATGTGCCTGACCCAATGGTAGGC	E2F transcription factor 3
EDN1	NM_001955.2	ET1	TTTCATGATCCCAAGCTGAAAGGCAATCCCTCCAGAGAGCGTTATGTGACCCACAACCGAGCACATTGGTGACAGACCTTCGGGGCCTGTCTGAAGCCAT	endothelin 1
EGF	NM_001178130.2	epidermal growth factor (beta-urogastrone);Pro-epidermal growth factor	AGACAGAGGGAAATCTCTGATTGGAAGGAGTGATTTAAATGGGAAACGTTCCAAAATAATCACTAAGGAGAACATCTCTCAACCACGAGGAATTGCTGTT	epidermal growth factor
EG	NM	ERBB;epidermal growth factor receptor (avian erythroblastic leukemia viral (v-erb-b) oncogene	GCAGCCAGGAACGTACTGGTGAAA	epidermal growth factor receptor

FR	_005228.3	homolog);ERBB1;erythroblastic leukemia viral (v-erb-b) oncogene homolog (avian),erb-b2 receptor tyrosine kinase 1	ACACCGCAGCATGTCAAGATCACAGATTTTGGGCTGGCCAAACTGCTGGGTGCGGAAGAGAAAGAATACCATGCAG	
EGR1	NM_001964.2	TIS8,G0S30,NGFI-A,KROX-24,ZIF-268,AT225,ZNF225;nerve growth factor-induced protein A,transcription factor ETR103,zinc finger protein 225,early growth response protein 1	TCTCTACTGGAGTGGAAGGTCTATTGGCCAACAATCCTTTCTGCCCACTTCCCCTTCCCCAATTACTATTCCCTTTGACTTCAGCTGCCTGAAACAGCCA	early growth response 1
EIF2AK2	NM_001135652.2	PRKR;protein kinase, interferon-inducible double stranded RNA dependent;PKR,EIF2AK1,PPP1R83;protein phosphatase 1, regulatory subunit 83	AGAAGAAACCTCAGTGAAATCTGACTACCTGTCCTCTGGTTCTTTTGCTACTACGTGTGAGTCCCAAAGCAACTCTTTAGTGACCAGCACACTCGCTTCT	eukaryotic translation initiation factor 2 alpha kinase 2
EIF2B4	NM_001034116.1	eukaryotic translation initiation factor 2B, subunit 4 (delta, 67kD),eukaryotic translation initiation factor 2B, subunit 4 delta, 67kDa;EIF2Bdelta,EIF-2B,DKFZP586J0119,EIF2B	ACTACGTTCTCTAGTCCATGCTGGTGTCCCAGCCTCCTACCTGCTGATTCCTGCAGCCTCCTATGTGCTCCCAGAGGTTTCCAAGGTGCTATTGGGAGCT	eukaryotic translation initiation factor 2B subunit delta
EIF4EBP1	NM_004095.3	PHAS-I,4E-BP1;phosphorylated heat- and acid-stable protein regulated by insulin 1	ACACCCTGCAGCCAAGGGCCAGGAAGTGGACAAGAACGAACCCTTCCTTCCGAATGATCAGCAGTTCCAGCCCCTCGCTGCTGGGGGCGCAACCACCCCT	eukaryotic translation initiation factor 4E binding protein 1
EIF5AL1	NM_001099692.1	EIF5AP1;eukaryotic translation initiation factor 5A pseudogene 1;bA342M3.3	AAAGGAAACACGAAGATTAATCAAGCAGGAAGGACAAGCTCAGTTTTGCACCCACTGAATTTGCCACAAATATTGTGGAAAATATTCTCGGGGACATTGC	eukaryotic translation initiation factor 5A like 1
EL	NM_0	TCEB2;transcription elongation factor B (SIII), polypeptide 2 (18kDa, elongin B),transcription elongation factor B subunit 2;SIII	CTGCATGTCCACTCCCAGACGATGGCCAAGAGCAG	elongin B

OB	07108.2		AAACACAAGCTGGAGCCAGTGTCCTGGTTTGACAGCATGTTCAACGAGGGAACCCCAAGACGGAC	
ENO1	NM_001428.3	ENO1L1,MPB1;enolase 1, (alpha);PPH,MBP-1;alpha-enolase	AGAGAAGATTGACAAACTGATGATCGAGATGGATGGAACAGAAAATAAATCTAAGTTTGGTGCGAACGCCATTCTGGGGGTGTCCCTTGCCGTCTGCAAA	enolase 1
ENTPD1	NM_001098175.1	CD39;NTPDase-1,ATPDase,SPG64	AGTACCTGAGTGAATACTGCTTTTCTGGTACCTACATTCTCTCCCTCCTTCTGCAAGGCTATCATTTCACAGCTGATTCCTGGGAGCACATCCATTTCAT	ectonucleoside triphosphate diphosphohydrolase 1
EOMES	NM_001278182.1	eomesodermin (Xenopus laevis) homolog;TBR2;T-box brain2	TCATTCGGGAAATTAAAACTCACCAATAACAAAGGCGCAAATAACAACAACACCCAGATGATAGTCTTACAATCCTTACACAAATACCAACCCCGACTGC	eomesodermin
EPCAM	NM_002354.2	M4S1,MIC18,TACSTD1;antigen identified by monoclonal antibody AUA1,tumor-associated calcium signal transducer 1;Ly74,TROP1,GA733-2,EGP34,EGP40,EGP-2,KSA,CD326,Ep-CAM,HEA125,KS1/4,MK-1,MH99,MOC31,323/A3,17-1A,TACST-1,CO-17A,ESA;trophoblast cell surface antigen 1	TGTACTTCAGTTGGTGCACAAAATACTGTCATTTGCTCAAAGCTGGCTGCCAAATGTTTGGTGATGAAGGCAGAAATGAATGGCTCAAAACTTGGGAGAA	epithelial cell adhesion molecule
EPM2AIP1	NM_014805.3	EPM2A (laforin) interacting protein 1;KIAA0766,FLJ11207;laforin interacting protein 1	GGGGCAACAACAGTCCACTTCTCAGACAAACAATGGCTTTGTGACTTTGGCTTCTTGGTGGACATTATGGAACACCTTCGAGAACTCAGTGAAGAATTAC	EPM2A interacting protein 1
ERBB2	NM_00444	NGL;v-erb-b2 avian erythroblastic leukemia viral oncogene homolog 2 (neuro/glioblastoma derived oncogene homolog),v-erb-b2 avian erythroblastic leukemia viral oncogene homolog 2;NEU,HER-2,CD340,HER2;neuro/glioblastoma derived oncogene homolog,human epidermal growth factor receptor 2	TGAAGGTGCTTGGATCTGGCGCTTTTGGCACAGTCTACAAGGGCATCTGGATCCCTGATGGGGAGAATGTGAAAATTCCAGTG	erb-b2 receptor tyrosine kinase 2

	8.2		GCCATCAAAGTGTTGAG	
ERO1A	NM_014584.2	ERO1L;ERO1 (S. cerevisiae)-like,ERO1-like (S. cerevisiae);ERO1-alpha,Ero1alpha	CAGTCCCCTGAAGCTGAATATGTAGATTTGCTTCTTAATCCTGAGCGCTACACTGGTTACAAGGGACCAGATGCTTGGAAAATATGGAATGTCATCTACG	endoplasmic reticulum oxidoreductase 1 alpha
ESR1	NM_000125.2	ESR;NR3A1,Era,ER-alpha;nuclear receptor subfamily 3 group A member 1,estrogen receptor alpha,oestrogen receptor alpha,E2 receptor alpha	AGGAACCAGGGAAAATGTGTAGAGGGCATGGTGGAGATCTTCGACATGCTGCTGGCTACATCATCTCGGTTCCGCATGATGAATCTGCAGGGAGAGGAGT	estrogen receptor 1
EXO1	NM_006027.3	HEX1,hExoI;rad2 nuclease family member, homolog of S. cerevisiae exonuclease 1	TGGCCCACAAAGTAATTAAAGCTGCCCGGTCTCAGGGGGTAGATTGCCTCGTGGCTCCCTATGAAGCTGATGCGCAGTTGGCCTATCTTAACAAAGCGGG	exonuclease 1
EZH2	NM_004456.4	enhancer of zeste (Drosophila) homolog 2,enhancer of zeste homolog 2 (Drosophila);EZH1,ENX-1,KMT6,KMT6A	TAGACAGGTGTATGAGTTTAGAGTCAAAGAATCTAGCATCATAGCTCCAGCTCCCGCTGAGGATGTGGATACTCCTCCAAGGAAAAAGAAGAGGAAACAC	enhancer of zeste 2 polycomb repressive complex 2 subunit
F2RL1	NM_005242.4	GPR11;coagulation factor II (thrombin) receptor-like 1;PAR2;proteinase-activated receptor-2	GGGAATTGCACAGTAGGATGTGGAACCTGTTTAATGTTATGAGGACGTGTCTGTTATTTCCTAATCAAAAAGGTCTCACCACATACCATGTGGATGCAGC	F2R like trypsin receptor 1
FADD	NM_003824.3	Fas (TNFRSF6)-associated via death domain;MORT1,GIG3;Fas-associating protein with death domain,Fas-associating death domain-containing protein,mediator of receptor-induced toxicity,growth-inhibiting gene 3 protein	CTGGTGGCTGACCTGGTACAAGAGGTTCAGCAGGCCCGTGACCTCCAGAACAGGAGTGGGGCCATGTCCCCGATGTCATGGAACTCAGACGCATCTACCT	Fas associated via death domain
FAM124B	NM_0011222	family with sequence similarity 124B;FLJ22746	CACAAGATCAGATAAAATGTTCTAGAAATGGAGTACTGACCCAGAATCAGCCTGTTAGTTGTTCATCTGAGGGGGGCTGAATG	family with sequence similarity 124 member B

	779.1		CTCCCCATGCACAATCT	
FAM30A	NR_026800.2	C14orf110,KIAA0125;chromosome 14 open reading frame 110,KIAA0125;HSPC053	ACAGTTCTGAAGTCAAAGGCTGATGTCCTGTTTCTCTTTCCCTCTGTGACCGACTCCCTTCCCAGTGGTAACAAGTACCCACAGCTTGGTTTGAATTTCT	family with sequence similarity 30 member A
FANCA	NM_001018112.1	FACA,FANCH;Fanconi anemia complementation group A;FAA,FA-H,FAH	AAGCGGTGTGGCATCTTCACGTACAAGGCATTGTGAGCCTGCAAGAGCTGCTGGAAAGCCATCCCGACATGCATGCTGTGGGATCGTGGCTCTTCAGGAA	FA complementation group A
FAP	NM_004460.2	fibroblast activation protein, alpha;DPPIV;seprase	GAGTTGCCACCTCTGCTGTGCTTGCCTTATTGGTGATGTGCATTGTCTTACGCCCTTCAAGAGTTCATAACTCTGAAGAAAATACAATGAGAGCACTCAC	fibroblast activation protein alpha
FAS	NM_000043.3	FAS1,APT1,TNFRSF6;tumor necrosis factor receptor superfamily, member 6,Fas (TNF receptor superfamily, member 6);CD95,APO-1;TNF receptor superfamily member 6	CACCGGGGCTTTTCGTGAGCTCGTCTCTGATCTCGCGCAAGAGTGACACACAGGTGTTCAAAGACGCTTCTGGGGAGTGAGGGAAGCGGTTTACGAGTGA	Fas cell surface death receptor
FASLG	NM_000639.2	APT1LG1,TNFSF6;tumor necrosis factor (ligand) superfamily, member 6,Fas ligand (TNF superfamily, member 6);FasL,CD178	CCTGAAAAAAAGGAGCTGAGGAAAGTGGCCCATTTAACAGGCAAGTCCAACTCAAGGTCCATGCCTCTGGAATGGGAAGACACCTATGGAATTGTCCTGC	Fas ligand
FBP1	NM_001127628.1	FBP;fructose-1,6-bisphosphatase 1	AAGTCATCCTTTGCCACGTGTGTTCTCGTGTCAGAAGAAGATAAACACGCCATCATAGTGGAACCGGAGAAAAGGGGTAAATATGTGGTCTGTTTTGATC	fructose-bisphosphatase 1

FCAR	NM_133278.2	Fc fragment of IgA, receptor for;CD89	TCCACCAAGATTACACGACGCAGAACTTGATCCGCATGGCCGTGGCAGGACTGGTCCTCGTGGCTCTCTTGGCCATACTGGTTGAAAATTGGCACAGCCA	Fc fragment of IgA receptor
FCGR1A	NM_000566.3	Fc fragment of IgG, high affinity Ia, receptor for (CD64),Fc fragment of IgG, high affinity Ia, receptor (CD64);CD64,CD64A;Fc gamma receptor Ia	TGGGAAAGCATCGCTACACATCAGCAGGAATATCTGTCACTGTGAAAGAGCTATTTCCAGCTCCAGTGCTGAATGCATCTGTGACATCCCCACTCCTGGA	Fc fragment of IgG receptor Ia
FCGR2A	NM_021642.3	FCG2,FCGR2A1,FCGR2;Fc fragment of IgG, low affinity IIa, receptor for (CD32),Fc fragment of IgG, low affinity IIa, receptor (CD32);CD32,CD32A,IGFR2,CDw32;Immunoglobulin G Fc receptor II,Fc gamma receptor IIa	TGGAGACCCAAATGTCTCAGAATGTATGTCCCAGAAACCTGTGGCTGCTTCAACCATTGACAGTTTTGCTGCTGCTGGCTTCTGCAGACAGTCAAGCTGC	Fc fragment of IgG receptor IIa
FCGR2B	NM_001002273.1	FCG2,FCGR2;Fc fragment of IgG, low affinity IIb, receptor for (CD32),Fc fragment of IgG, low affinity IIb, receptor (CD32);CD32,CD32B;Fc gamma receptor IIb	AGGCTGACAAAGTTGGGGCTGAGAACACAATCACCTATTCACTTCTCATGCACCCGGATGCTCTGGAAGAGCCTGATGACCAGAACCGTATTTAGTCTCC	Fc fragment of IgG receptor IIb
FCGR3A/B	NM_000570.4	FCGR3,FCG3;Fc fragment of IgG, low affinity IIIb, receptor for (CD16),Fc fragment of IgG, low affinity IIIb, receptor (CD16b);CD16,CD16b;Fc gamma receptor IIIb	TGAAGTGCCAGGGAGCCTACTCCCCTGAGGACAATTCCACACAGTGGTTTCACAATGAGAACCTCATCTCAAGCCAGGCCTCGAGCTACTTCATTGACGC	Fc fragment of IgG receptor IIIb
FCGRT	NM_004107.4	Fc fragment of IgG, receptor, transporter, alpha;FCRN,alpha-chain;heavy chain of the major histocompatibility complex class I-like Fc receptor,transmembrane alpha chain of the neonatal receptor	CCAGGGGAGGCCCAGGATGCTGATTTGAAGGATGTAAATGTGATTCCAGCCACCGCCTGACCATCCGCCATTCCGACTGCTAAAAGCGAATGTAGTCAGG	Fc fragment of IgG receptor and transporter
FCN1	NM_002000	ficolin (collagen/fibrinogen domain-containing) 1,ficolin (collagen/fibrinogen domain containing) 1;FCNM	TACGCCGACTGTCATGCTTCAAACCTCAATGGTCTCTACCTCATGGGACCCCATGAGAGCTATGCCAATGGTATCAACTGGAGT	ficolin 1

	3.4		GCGGCGAAGGGGTACA	
FCRL2	NM_001159488.1	SPAP1;SH2 domain-containing phosphatase anchor protein 1,Fc receptor-like 2;FCRH2,IRTA4,CD307b	CCTCTTCTGTCTTCGAAGGAGACAGCATCGTTCTGAAATGCCAGGGAGAACAGAACTGGAAAAATTCAGAAGATGGCTTACCATAAGGATAACAAAGAGTT	Fc receptor like 2
FGF13	NM_001139498.1	LINC00889;long intergenic non-protein coding RNA 889;FHF2,FGF2,FLJ30672;fibroblast growth factor homologous factor 2	CAAACAAACAGGCAGAGTTCACTATTCTATCTGCCATTAGACCTTCTTATCATCCATACTAAAGCCCCATTATTTAGATTGAGCTTGTGCATAAGAATGC	fibroblast growth factor 13
FGF18	NM_003862.2	FGF-18,ZFGF5	CCCGGGAGAACCAGCAGGACGTGCATTTCATGAAGCGCTACCCCAAGGGGCAGCCGGAGCTTCAGAAGCCCTTCAAGTACACGACGGTGACCAAGAGGTC	fibroblast growth factor 18
FGF9	NM_002010.2	fibroblast growth factor 9 (glia-activating factor);glia-activating factor	GTGGAATGGGGGTTCTGGTTTCACAAACAGATGCTTAGATAGCCAAACCACTGTCTTGTTGGTGCCAACACTTGCACTGTGGTCAAAGACTTACCGAGCA	fibroblast growth factor 9
FGFR1	NM_001174064.1	FLT2,KAL2;fms-related tyrosine kinase 2;H2,H3,H4,H5,CEK,FLG,BFGFR,N-SAM,CD331;Pfeiffer syndrome	GCGGTCCCCTCACCGGCCCATCCTGCAAGCAGGGTTGCCCGCCAACAAAACAGTGGCCCTGGGTAGCAACGTGGAGTTCATGTGTAAGGTGTACAGTGAC	fibroblast growth factor receptor 1
FLNB	NM_001164	FLN1L,LRS1;filamin B, beta (actin binding protein 278),Larsen syndrome 1 (autosomal dominant),filamin B, beta;TAP,TABP,ABP-278,FH1;actin binding protein 278,beta filamin	TTATTGGTGGTCTTTTCACCATTGGCAGAAACAGTGAGAGCTGTGTGGTGCAGAAATCCAGAAATGAGGTGTAGGGAATTTTGCC	filamin B

	319.1		TGCCTTCCTGCAGAC	
FLT1	NM_002019.4	FLT;fms-related tyrosine kinase 1 (vascular endothelial growth factor/vascular permeability factor receptor),fms-related tyrosine kinase 1,fms related tyrosine kinase 1;VEGFR1;vascular endothelial growth factor receptor 1,vascular permeability factor receptor	AAGAAATGGCAAACAATTCTGCAGTACTTTAACCTTGAACACAGCTCAAGCAAACCACACTGGCTTCTACAGCTGCAAATATCTAGCTGTACCTACTTCA	fms related receptor tyrosine kinase 1
FOSL1	NM_005438.3	FOS like antigen 1;fra-1	CCCTTCCAGATCACTGCCACACTCTCCATCACCCTCTTCCTGTGATCCACCCAACCCTATCTCCTGACAGAAGGTGCCACTTTACCCACCTAGAACACTA	FOS like 1, AP-1 transcription factor subunit
FOXP3	NM_014009.3	IPEX;immune dysregulation, polyendocrinopathy, enteropathy, X-linked;JM2,XPID,AIID,PIDX,DIETER,SCURFIN	GGGCCATCCTGGAGGCTCCAGAGAAGCAGCGGACACTCAATGAGATCTACCACTGGTTCACACGCATGTTTGCCTTCTTCAGAAACCATCCTGCCACCTG	forkhead box P3
FPR1	NM_002029.3	FPR,FMLP	TTTTAACTTTTCGCCCTGGACCAACGACCCTAAAGAGAGGATAAATGTGGCCGTTGCCATGTTGACGGTGAGAGGCATCATCCGGTTCATCATTGGCTTC	formyl peptide receptor 1
FPR3	NM_002030.4	FPRL2;formyl peptide receptor-like 2;FPRH1,FMLPY,RMLP-R-I	TTTGCATTCTGGGGTGACACTGCTGTAGAGAGGTTGAACGTGTTCATTACCATGGCCAAGGTCTTTCTGATCCTCCACTTCATTATTGGCTTCAGCGTGC	formyl peptide receptor 3
FSTL3	NM_005860.2	follistatin-like 3 (secreted glycoprotein);FLRG,FSRP;follistatin-related protein	TCACCCACCCGGGGAACAAGATCAACCTCCTCGGCTTCTTGGGCCTTGTCCACTGCCTTCCCTGCAAAGATTCGTGCGACGGCGTGGAGTGCGGCCCGGG	follistatin like 3
FUT4	NM_0020	CD15,FCT3A,ELFT;ELAM ligand fucosyltransferase;FUC-TIV;galactoside 3-L-fucosyltransferase,alpha (1,3) fucosyltransferase, myeloid-specific	AGCGCTGGGTTTGGATGAACTTCGAGTCGCCCTCGCACTCCCCGGGGCTGCGAAGCCTGGCAAGTAACCTCT	fucosyltransferase 4

	33.3		TCAACTGGACGCTCTCCTACCGGGCGGA	
FYN	NM_002037.5	FYN oncogene related to SRC, FGR, YES;SYN,SLK,MGC45350	CCTGAAGAACGCCCCACTTTTGAGTACTTGCAGAGCTTCCTGGAAGACTACTTTACCGCGACAGAGCCCCAGTACCAACCTGGTGAAAACCTGTAAGGCC	FYN proto-oncogene, Src family tyrosine kinase
FZD8	NM_031866.1	frizzled (Drosophila) homolog 8,frizzled homolog 8 (Drosophila),frizzled 8, seven transmembrane spanning receptor,frizzled family receptor 8	GTCTCCACCTTCCTTATCGACATGGAGCGCTTCAAGTACCCGGAGCGGCCCATTATCTTCCTCTCGGCCTGCTACCTCTTCGTGTCGGTGGGCTACCTAG	frizzled class receptor 8
FZD9	NM_003508.2	frizzled (Drosophila) homolog 9,frizzled homolog 9 (Drosophila),frizzled 9, seven transmembrane spanning receptor,frizzled family receptor 9;FZD3,CD349	CTCTGGCTACCTGGTGCTGGGCAGTAGTTTCCTCCTGACCGGCTTCGTGGCCCTCTTCCACATCCGCAAGATCATGAAGACGGGCGGCACCAACACAGAG	frizzled class receptor 9
GAS1	NM_002048.2	Growth arrest-specific gene-1	ACCTGGCGCTGAGCCGCTACCTGACCTACTGCGGCAAAGTCTTCAACGGGCTGCGCTGCACGGACGAATGCCGCACCGTCATTGAGGACATGCTGGCTAT	growth arrest specific 1
GBP1	NM_002053.2	guanylate binding protein 1, interferon-inducible, 67kDa,guanylate binding protein 1, interferon-inducible	TCCAGTTGCTGAAAGAGCAAGAGAGGACCCTCGCTCTTAAACTTCAGGAACAGGAGCAACTACTAAAAGAGGGATTTCAAAAGAAAGCAGAATAATGAA	guanylate binding protein 1
GBP2	NM_004120.4	guanylate binding protein 2, interferon-inducible	TCAGTCACTCTCAGAAAAGGAAAAAGCGATTGAAGTGGAACGTATAAAGGCTGAATCTGCAGAAGCTGCAAAGAAAATGTTGGAGGAAATACAAAAGAAG	guanylate binding protein 2
GBP4	NM_05294	Mpa2	TGGAGGAGGAAAGGGAAAACCTTCTCAGAGAGCATGAAAGGCTGCTAAAACACAAGCTGAAGGTACAAGAAGAAATGCTTAAG	guanylate binding protein 4

	1.4		GAAGAATTTCAAAAGAA	
GHR	NM_000163.2	GHBP;growth hormone binding protein	TTCATATAGTACAGTCCCCACAGGGCCTCATACTCAATGCGACTGCCTTGCCCTTGCCTGACAAAGAGTTTCTCTCATCATGTGGCTATGTGAGCACAGA	growth hormone receptor
GIMAP4	NM_018326.2	HIMAP4,FLJ11110,IMAP4,IAN1;immune-associated nucleotide-binding protein 1	AACAGAACTTGTCGTAGTTGACACACCAGGCATTTTCGACACAGAGGTGCCCAATGCTGAAACGTCCAAGGAGATTATTCGCTGCATTCTTCTGACCTCC	GTPase, IMAP family member 4
GIMAP6	NR_024115.1	FLJ22690,IAN6;immune-associated nucleotide-binding protein 6	CTGCCTGGTTTATGGGAGAACTCTGAAGCCCATGATGTTTTCCTGGAAGGCTGTTCCTACAGACGGGGTTTATAAGTACTGATACTGGACAGGATGAGAA	GTPase, IMAP family member 6
GLI1	NM_005269.2	GLI;glioma-associated oncogene homolog 1 (zinc finger protein),glioma-associated oncogene family zinc finger 1	GGACCTGGCTTTGGACCCAACTTGCCCAATCACAAGTCAGGTTCCTATCCCACCCCTTCACCATGCCATGAAAATTTTGTAGTGGGGGCAAATAGGGCTT	GLI family zinc finger 1
GLS	NM_014905.4	KIAA0838,GLS1	ATCTCACCGCCCCACCACAGACCGCGTTCCCCGAGGAAACCGGCCGCCCACGCCCGGAGCATCCTCCCCTGTTGAGCGGGCGCTGACGGACCCGGCGGCA	glutaminase
GLUD1	NM_005271.2	GLUD;GDH	GTGTCCCAGAAAAAAACCACTTGGGCTCCCTGTTTGGAGTCTGGCTGGCTCTGAGCATTGCCAATGGCCCCTACTCACCTGACTTTGTATCCTCTCCTTT	glutamate dehydrogenase 1
GLUL	NM_002006	GLNS;glutamate-ammonia ligase (glutamine synthase);glutamine synthetase	GTCATGCCTGCCCAGTGGGAATTTCAGATTGGACCTTGTGAAGGAATCAGCATGGGAGATCATCTCTGGGTGGCCCGTTTCATCT	glutamate-ammonia ligase

	5.<br>6		TGCATCGTGTGT<br>GTG	
G<br>M<br>I<br>P	N<br>M<br>_0<br>0<br>1<br>2<br>8<br>8<br>9<br>9<br>8.<br>1	ARHGAP46	CTTCCTGCAGCT<br>ACCCAGGGACTT<br>CCCGGAGGAGG<br>TACCCTTTGTGG<br>TCACGAAGTGCA<br>CGGCTGAGATAG<br>AACACCGTGCCC<br>TGGATGTGCAGG<br>GCATT	GEM<br>interactin<br>g protein
G<br>N<br>G<br>4	N<br>M<br>_0<br>0<br>1<br>0<br>9<br>8<br>7<br>2<br>1.<br>1	guanine nucleotide binding protein (G protein),<br>gamma 4	CAAACACCTTTG<br>TTGCAGTAACCC<br>GTGGCACACTTC<br>ACTTCCCACATTC<br>TCCAGGAGGAA<br>GCCGTATTCTGG<br>TAGTTTAAGGAA<br>GGTGATTCATGG<br>ATGC	G protein subunit gamma 4
G<br>N<br>L<br>Y	N<br>M<br>_0<br>1<br>2<br>4<br>8<br>3.<br>3	LAG2;NKG5,LAG-2,D2S69E,TLA519;T-lymphocyte<br>activation gene 519	ACCAAAACACAG<br>GAGCTGGGCCGT<br>GACTACAGGACC<br>TGTCTGACGATA<br>GTCCAAAAACTG<br>AAGAAGATGGT<br>GGATAAGCCCAC<br>CCAGAGAAGTGT<br>TTCCA	granulysi<br>n
G<br>O<br>T<br>1	N<br>M<br>_0<br>0<br>2<br>0<br>7<br>9.<br>2	glutamic-oxaloacetic transaminase 1, soluble<br>(aspartate aminotransferase 1),glutamic-<br>oxaloacetic transaminase 1, soluble;AST1;aspartate<br>aminotransferase 1,aspartate transaminase<br>1,glutamate oxaloacetate transaminase 1	GCGCGTTGGTAC<br>AATGGAACAAAC<br>AACAAGAACACA<br>CCTGTCTATGTG<br>TCCTCACCAACCT<br>GGGAGAATCACA<br>ATGCTGTGTTTTC<br>CGCTGCTGGTTT<br>TA	glutamic-oxaloacetic<br>transaminase 1
G<br>O<br>T<br>2	N<br>M<br>_0<br>0<br>2<br>0<br>8<br>0.<br>3	glutamic-oxaloacetic transaminase 2, mitochondrial<br>(aspartate aminotransferase 2),glutamic-<br>oxaloacetic transaminase 2,<br>mitochondrial;mitAAT,KATIV,KAT4,KYAT4;kynureni<br>ne aminotransferase IV,aspartate aminotransferase<br>2,aspartate transaminase 2	TGACCCCAAGAC<br>TTGCGGTTTTGA<br>CTTCACAGGCGC<br>TGTGGAGGATAT<br>TTCAAAAATACC<br>AGAGCAGAGTGT<br>TCTTCTTCTGCAT<br>GCCTGCGCCCAC<br>AAT	glutamic-oxaloacetic<br>transaminase 2
G<br>P<br>C<br>4	N<br>M<br>_0<br>0<br>1<br>4<br>4<br>8.<br>2	K-glypican;glypican proteoglycan 4	CCCTCGCAAATT<br>GAAGCTCCAGGT<br>TACTCGTGCTTTT<br>GTAGCAGCCCGT<br>ACTTTCGCTCAA<br>GGCTTAGCGGTT<br>GCGGGAGATGT<br>CGTGAGCAAGGT<br>CTCC	glypican 4
G<br>P	N<br>M	GPCR150,GPCR1	GGATTTCAGTCC<br>TTGCTTATGTTTT	G protein-coupled receptor 160

R160	_014373.1		GGGAGACCCAGCCATCTACCAAAGCCTGAAGGCACAGAATGCTTATTCTCGTCACTGTCCTTTCTATGTCAGCAT	
GPSM3	NM_001276501.1	C6orf9;chromosome 6 open reading frame 9,G-protein signalling modulator 3 (AGS3-like, C. elegans),G-protein signaling modulator 3;NG1,G18,G18.1a,G18.1b,G18.2,AGS4;activator of G-protein signaling 4	ACTGGTCTCAAAGCTGGGCAGCCCATTGCATGCCCTCAACTCTTGCTTGGCAGGGGTACCAGAGACTGAAAGACACGGCACAAATCTCAATATTCATCTC	G protein signaling modulator 3
GZMA	NM_006144.3	HFSP,CTLA3;granzyme A (granzyme 1, cytotoxic T-lymphocyte-associated serine esterase 3);CTL tryptase,Cytotoxic T-lymphocyte-associated serine esterase-3,Hanukah factor serine protease),granzyme 1	CAAAAGACTGGGTGTTGACTGCAGCTCACTGTAACTTGAACAAAAGGTCCCAGGTCATTCTTGGGGCTCACTCAATAACCAGGGAAGAGCCAACAAAACA	granzyme A
GZMB	NM_004131.4	CTLA1,CSPB;granzyme B (granzyme 2, cytotoxic T-lymphocyte-associated serine esterase 1);CCPI,CGL-1,CSP-B,CGL1,CTSGL1,HLP,SECT;fragmentin 2,cytotoxic serine protease B,cathepsin G-like 1,T-cell serine protease 1-3E,granzyme 2,cytotoxic T-lymphocyte-associated serine esterase 1	GAGTTGTGCGTGGGGGACCCAGAGATTAAAAAGACTTCCTTTAAGGGGGACTCTGGAGGCCCTCTTGTGTGTAACAAGGTGGCCCAGGGCATTGTCTCCT	granzyme B
GZMH	NM_033423.3	CTSGL2;granzyme H (cathepsin G-like 2, protein h-CCPX);CGL-2,CCP-X,CTLA1,CSP-C;cathepsin G-like 2, protein h-CCPX	AAAAAAGGGACACCTCCAGGAGTCTACATCAAGGTCTCACACTTCCTGCCCTGGATAAAGAGAACAATGAAGCGCCTCTAACAGCAGGCATGAGACTAAC	granzyme H
GZMK	NM_002104.2	granzyme K (serine protease, granzyme 3; tryptase II),granzyme K (granzyme 3; tryptase II);TRYP2,PRSS;tryptase II	GGTCTGTGCAGGAGATGCCAAAGGCCAGAAGGATTCCTGTAAGGGTGACTCAGGGGGCCCCTTGATCTGTAAAGGTGTCTTCCACGCTATAGTCTCTGGA	granzyme K
GZMM	NM_005317.2	granzyme M (lymphocyte met-ase 1);MET1,LMET1;lymphocyte met-ase 1	GGGCCCCTGGTGTGTGGCAAAGGCCGGGTGTTGGCCGGAGTCCTGTCCTTCAGCTCCAGGGTCTGCACTGACATCTTCAAGCCTCCCGTGGCCACCGCTG	granzyme M

H2AFX	NM_002105.2	H2AFX;H2A histone family, member X,H2A histone family member X	GCCGGCCGCCAGGCCTCCCCATGCCACCACAAAGGCCCTTTTAAGGGCCACCACCGCCCTCATGGAAAGAGCTGAGCCGCTTCAGACTGCGGGGCAAGCG	H2A.X variant histone
HAVCR2	NM_032782.3	Tim-3,TIM3,FLJ14428,TIMD3,CD366;T-cell immunoglobulin mucin family member 3	TATATGAAGTGGAGGAGCCCAATGAGTATTATTGCTATGTCAGCAGCAGGCAGCAACCCTCACAACCTTTGGGTTGTCGCTTTGCAATGCCATAGATCCA	hepatitis A virus cellular receptor 2
HCK	NM_001172129.1	hemopoietic cell kinase;JTK9	TCCAGGTCGGAGGCAATACATTCTCAAAAACTGAAACCAGCGCCAGCCCACACTGTCCTGTGTACGTGCCGGATCCCACATCCACCATCAAGCCGGGGCC	HCK proto-oncogene, Src family tyrosine kinase
HDAC11	NM_001330636.1		GCACAGCCCGCATCATTGCTGACTCCATACTTAATCTGTTTGGCCTGGGGCTCATTGGGCCTGAGTCACCCAGCGTCTCCGCACAGAACTCAGACACACC	histone deacetylase 11
HDAC3	NM_003883.3	RPD3,HD3,RPD3-2,KDAC3	CTCTGGGAAAGAGTCTGGAGACCACATTTGGTTCTCGAACCATCTACCTGCTTTTCCTCTCTCTCCCAAGGCCTGACAATGGTACCTATTAGGGATGAGA	histone deacetylase 3
HDAC4	NM_006037.3	BDMR;brachydactyly-mental retardation syndrome;KIAA0288,HDAC-A,HDACA,HD4,HA6116,HDAC-4	CCTCGGAAGCATGTGTTTCTGCCTTGCTGGGAAACGAGCTTGATCCTCTCCCAGAAAAGGTTTTACAGCAAAGACCCAATGCAAACGCTGTCCGTTCCAT	histone deacetylase 4
HDA	NM_00	KIAA0600,NY-CO-9,FLJ90614	GTGCTGAGGAGGGGCCCGACTTGGAGGAGCCTGGTGCTGGATACAA	histone deacetylase 5

C5	1015053.1		AAAACTGTTCTCAGATGCCCAGCCGCTGCAGCCTTTGCAGGTGTACCAGGCGCC	
HDC	NM_002112.3		CATCCCGGCCACTATCCAGGACAAGTTAATCATCCGTTTCACTGTGACATCCCAGTTTACCACTAGGGATGACATCCTGAGAGACTGGAATCTCATTCGA	histidine decarboxylase
HELLS	NM_001289067.1	PASG,SMARCA6,LSH,Nbla10143;SWI/SNF2-related, matrix-associated, actin-dependent regulator of chromatin, subfamily A, member 6,proliferation-associated SNF2-like protein	GAGGCTCCAGCAATGGTTGAACAACTGGACACTGCTGTGATTACCCCGGCCATGCTAGAAGAGGAAGAACAGCTTGAAGCTGCTGGACTAGAGAGAGAGC	helicase, lymphoid specific
HERC6	NM_001165136.1	hect domain and RLD 6;FLJ20637	AGAATCTAGGTGTGGTTTATATCAGCTGTGGTGATGCACACACTGCGGTGCTTACCCAGGACGGGAAAGTGTTCACATTTGGAGACAATCGCTCTGGACA	HECT and RLD domain containing E3 ubiquitin protein ligase family member 6
HES1	NM_005524.2	HRY;hairy homolog (Drosophila),hairy and enhancer of split 1, (Drosophila);FLJ20408,HES-1,bHLHb39	GCTGGAGAGGCGGCTAAGGTGTTTGGAGGCTTCCAGGTGGTACCGGCTCCCGATGGCCAGTTTGCTTTCCTCATTCCCAACGGGGCCTTCGCGCACAGCG	hes family bHLH transcription factor 1
HEY1	NM_001040708.1	hairy/enhancer-of-split related with YRPW motif 1,hes-related family bHLH transcription factor with YRPW motif 1;HESR-1,CHF2,HESR1,HRT-1,CHF-2,HERP2,bHLHb31	GTTGGTGGAAAGGAAACTGAAGGTAATTGAATAGAATACGCCTGCATTTACCAGCCCCAGCAACACAAAGAATTTTTAATCACACGGATCTCAAATTCAC	hes related family bHLH transcription factor with YRPW motif 1

HIF1A	NM_181054.2	hypoxia inducible factor 1, alpha subunit (basic helix-loop-helix transcription factor);MOP1,HIF-1alpha,PASD8,HIF1,bHLHe78	CTAGCCGAGGAAGAACTATGAACATAAAGTCTGCAACATGGAAGGTATTGCACTGCACAGGCCACATTCACGTATATGATACCAACAGTAACCAACCTCA	hypoxia inducible factor 1 subunit alpha
HK1	NM_000188.2		CTGTGGCCTGGCATCGCATCGTGGTGTGTCAATGCCACAAAATCGTGTGTCCGTGGAACCAGTCCTAGCCGCGTGTGACAGTCTTGCATTCTGTTTGTCT	hexokinase 1
HK2	NM_000189.4		TTTGGGGGGAAGCTCAGCCCAGAGCTTCTCAACACCGGTCGCTTTGAGACCAAAGACATCTCAGACATTGAAGGGGAGAAGGATGGCATCCGGAAGGCCC	hexokinase 2
HLA-A	NM_002116.7		GGGCGCCGTGGATAGAGCAGGAGGGGCCGGAGTATTGGGACCAGGAGACACGGAATGTGAAGGCCCAGTCACAGACTGACCGAGTGGACCTGGGGACCCT	major histocompatibility complex, class I, A
HLA-B	NM_005514.6	AS;ankylosing spondylitis	TGAATGTGTCTGCGTCCCTGTTAGCATAATGTGAGGAGGTGGAGAGACAGCCCACCCTTGTGTCCACTGTGACCCCTGTTCCCATGCTGACCTGTGTTTC	major histocompatibility complex, class I, B
HLA-C	NM_002117.4	HLA-JY3,D6S204,PSORS1;psoriasis susceptibility 1	TGGGAGCCATCTTCCCAGCCCACCATCCCCATCATGGGCATCGTTGCTGGCCTGGCTGTCCTGGTTGTCCTAGCTGTCCTTGGAGCTGTGGTCACCGCTA	major histocompatibility complex, class I, C
HLA-DMA	NM_006120.3	D6S222E,RING6	TCTGCGAGTGGATGATCCAGCAAATAGGGCCAAAACTTGATGGGAAAATCCCGGTGTCCAGAGGGTTTCCTATCGCTGAAGTGTTCACGCTGAAGCCCCT	major histocompatibility complex, class II, DM alpha

HLA-DMB	NM_002118.3	D6S221E,RING7	CCCGTGAGCTGGAAGGAACAGATTTAATATCTAGGGGCTGGGTATCCCCACATCACTCATTTGGGGGGTCAAGGGACCCGGGCAATATAGTATTCTGCTC	major histocompatibility complex, class II, DM beta
HLA-DOA	NM_002119.3	HLA-DZA,HLA-DNA;HLA-D0-alpha	CAGTGAACATGTCCACCCGACAGCTCCTGAGTTTATATCATCTCAACCCTCACAACCCACAGAGGCTGTGTCTCCTAGTCACAGCTTTAAATTACTGGAA	major histocompatibility complex, class II, DO alpha
HLA-DOB	NM_002120.3		GTGGCTCTGCTAGTGAATCTGACCCGACTGGATTCCTCCATGACTCAAGGCACAGACTCTCCAGAAGATTTTGTGATTCAGGCAAAGGCTGACTGTTACT	major histocompatibility complex, class II, DO beta
HLA-DPA1	NM_033554.2	HLA-DP1A	GGAGAGATCTGAACTCCAGCTGCCCTACAAACTCCATCTCAGCTTTTCTTCTCACTTCATGTGAAAACTACTCCAGTGGCTGACTGAATTGCTGACCCTT	major histocompatibility complex, class II, DP alpha 1
HLA-DPB1	NM_002121.5	HLA-DP1B	TTCCAAATTGGATACTGCTGCCAAGAAGTTGCTCTGAAGTCAGTTTCTATCATTCTGCTCTTTGATTCAAAGCACTGTTTCTCTCACTGGGCCTCCAACC	major histocompatibility complex, class II, DP beta 1
HLA-DQA1	NM_002122.3	HLA-DQA;CELIAC1	GGCGGTGGCCTGAGTTCAGCAAATTTGGAGGTTTTGACCCGCAGGGTGCACTGAGAAACATGGCTGTGGCAAAACACAACTTGAACATCATGATTAAACG	major histocompatibility complex, class II, DQ alpha 1
HLA-DQA2	NM_020056.4	HLA-DXA	CCTCGCCCTGACTGCCGTGATGAGCCCCTGTGGAGGTGAAGACATTGTGGCTGACCATGTTGCCTCCTATGGTGTGAACTTCTACCAGTCTCACGGTCCC	major histocompatibility complex, class II, DQ alpha 2

HLA-DQB1	NM_002123.3	HLA-DQB;IDDM1,CELIAC1	GGGGACCCGGGCGGAGTTGGACACGGTGTGCAGACACAACTACGAGGTGGCGTTCCGCGGGATCTTGCAGAGGAGAGTGGAGCCCACAGTGACCATCTCC	major histocompatibility complex, class II, DQ beta 1	
HLA-DRA	NM_019111.3	HLA-DRA1	GGCCAACATAGCTGTGGACAAAGCCAACCTGGAAATCATGACAAAGCGCTCCAACTATACTCCGATCACCAATGTACCTCCAGAGGTAACTGTGCTCACG	major histocompatibility complex, class II, DR alpha	
HLA-DRB1	NM_002124.3	HLA-DR1B	TTCGGCAACTGCAGAAAATGTCCTCCCTTGTGGCTTCCTCAGCTCCTGCCCTTGGCCTGAAGTCCCAGCATTGATGGCAGCGCCTCATCTTCAACTTTTG	major histocompatibility complex, class II, DR beta 1	HLA-DRB3 (NM_022555)
HLA-DRB5	NM_002125.3		GTCATTTCTTCAACGGGACGGAGCGGGTGCGGTTCCTGCACAGAGACATCTATAACCAAGAGGAGGACTTGCGCTTCGACAGCGACGTGGGGGAGTACCG	major histocompatibility complex, class II, DR beta 5	
HLA-E	NM_005516.5		CTATGTGTCTTAGGGGACTCTGGCTTCTCTTTTTGCAAGGGCCTCTGAATCTGTCTGTGTCCCTGTTAGCACAATGTGAGGAGGTAGAGAAACAGTCCAC	major histocompatibility complex, class I, E	
HLA-F	NM_001098479.1		CCTGCGGTTCGACAGCGACGCCGCGATTCCGAGGATGGAGCCGCGGGAGCCGTGGGTGGAGCAAGAGGGGCCGCAGTATTGGGAGTGGACCACAGGGTAC	major histocompatibility complex, class I, F	
HMGA1	NM_145900	HMGIY;high-mobility group (nonhistone chromosomal) protein isoforms I and Y	CCCATCTTCCACCTGTGCCCTCACCACCACACTACACAGCACACCAGCCGCTGCAGGGCTCCCATGGGCTGAGTGGGGAGCAGTT	high mobility group AT-hook 1	

	2.2		TTCCCCTGGCCTCAG	
HMGB1	NM_002128.5	HMG1;high-mobility group (nonhistone chromosomal) protein 1,high-mobility group box 1;HMG3,SBP-1,DKFZp686A04236;Sulfoglucuronyl carbohydrate binding protein,Amphoterin,high mobility group protein 1	GTGTCCTGTGTACCCGAGGGGTAATGATTAAATGATAAAGATAAGAAAAGCGCCCATGTAACACAAACTGCCATTCAACAGGTATTTCCCTTACTACCTA	high mobility group box 1
HNF1A	NM_000545.6	MODY3,TCF1;transcription factor 1, hepatic; LF-B1, hepatic nuclear factor (HNF1), albumin proximal factor;HNF1,LFB1	GTGCGCTATGGACAGCCTGCGACCAGTGAGACTGCAGAAGTACCCTCAAGCAGCGGCGGTCCCTTAGTGACAGTGTCTACACCCCTCCACCAAGTGTCCC	HNF1 homeobox A
HRAS	NM_005343.3	HRAS1;v-Ha-ras Harvey rat sarcoma viral oncogene homolog,Harvey rat sarcoma viral oncogene homolog	GGAAGGACGGAAGCAAGGAAGGAAGGAAGGGCTGCTGGAGCCCAGTCACCCCGGGACCGTGGGCCGAGGTGACTGCAGACCCTCCCAGGGAGGCTGTGCA	HRas proto-oncogene, GTPase
HSD11B1	NM_181755.2	HSD11B,HSD11;hydroxysteroid (11-beta) dehydrogenase 1;SDR26C1;short chain dehydrogenase/reductase family 26C, member 1	TGCCTTGCCCATGCTGAAGCAGAGCAATGGAAGCATTGTTGTCGTCTCCTCTCTGGCTGGGAAAGTGGCTTATCCAATGGTTGCTGCCTATTCTGCAAGC	hydroxysteroid 11-beta dehydrogenase 1
ICAM1	NM_000201.2	BB2,CD54;human rhinovirus receptor	AAATACTGAAACTTGCTGCCTATTGGGTATGCTGAGGCCCCACAGACTTACAGAAGAAGTGGCCCTCCATAGACATGTGTAGCATCAAAACACAAAGGCC	intercellular adhesion molecule 1
ICAM2	NM_000873.3	CD102	ACCTCTCTAGATAAGATTCTGCTGGACGAACAGGCTCAGTGGAAACATTACTTGGTCTCAAACATCTCCCATGACACGGTCCTCCAATGCCACTTCACCT	intercellular adhesion molecule 2
ICAM3	NM_00216	CDW50,ICAM-R,CD50	AGCGTCCAGCTGCGAGTCCTGTATGGTCCCAAAATTGACCGAGCCACATGCCCCCAGCACTTGAAATGGAAAGATAAAACGAGA	intercellular adhesion molecule 3

	2.3		CACGTCCTGCAGTGCC		
ICAM5	NM_003259.3	TLCN;TLN;telencephalin	CAGAGAGCTCCGAACCTTCTCCCTGTCTCCGGATGCCCCGCGCCTCGCTGCTCCCCGGCTCTTGGAAGTTGGCTCGGAAAGGCCCGTGAGCTGCACTCTG	intercellular adhesion molecule 5	
ICOS	NM_012092.3	inducible T-cell costimulator;AILIM,CD278;activation-inducible lymphocyte immunomediatory molecule	CCCTAACGGTGAATACATGTTCATGAGAGCAGTGAACACAGCCAAAAAATCTAGACTCACAGATGTGACCCTATAATATGGAACTCTGGCACCCAGGCAT	inducible T cell costimulator	
ICOSLG	NM_015259.4	ICOSL;inducible T-cell costimulator ligand;KIAA0653,GL50,B7-H2,B7RP-1,B7H2,B7RP1,ICOS-L,CD275,B7h;B7-related protein 1,B7 homologue 2,B7 homolog 2	CTGCTGGCGTTGGCTGTGATCCTGGAATGAGGCCCTTTCAAAAGCGTCATCCACACCAAAGGCAAATGTCCCCAAGTGAGTGGGCTCCCCGCTGTCACTG	inducible T cell costimulator ligand	LOC102723996 (NM_001363770)
ID4	NM_001546.3	inhibitor of DNA binding 4, dominant negative helix-loop-helix protein;bHLHb27;inhibitor of differentiation 4	GAAAAAAATGTTCTCTGCTTGCTACCAAAGGACAAACTCTTGGAAATGAACACTTTCTGCTTTCCTTCCTCCAAAGAATTAATAGGCAACAGTGGGAGAA	inhibitor of DNA binding 4, HLH protein	
IDO1	NM_002164.5	IDO,INDO;indoleamine-pyrrole 2,3 dioxygenase	ATTATAAGATGCTCTGAAAACTCTTCAGACACTGAGGGGCACCAGAGGAGCAGACTACAAGAATGGCACACGCTATGGAAAACTCCTGGACAATCAGTAA	indoleamine 2,3-dioxygenase 1	
IER3	NM_003897.3	IEX-1,DIF-2,PRG1,IEX-1L	ACCGAAAGCGCAGCCGCAGGGTTCTCTACCCTCGAGTGGTCCGGCGCCAGCTGCCAGTCGAGGAACCGAACCCAGCCAAAAGGCTTCTCTTTCTGCTGCT	immediate early response 3	
IFI16	NM_001206	IFNGIP1,PYHIN2	AGTGGGAATACCGGGGAGTTGAGATCTGTAATTCATAGTCACATCAAGGTCATCAAGACCAGGAAAAAACAAGAAAGACATACT	interferon gamma inducible protein 16	

	567.1		CAATCCTGATTCAAGTA	
IFI27	NM_005532.3	P27,FAM14D,ISG12	TCACTGGGAGCAACTGGACTCTCCGGATTGACCAAGTTCATCCTGGGCTCCATTGGGTCTGCCATTGCGGCTGTCATTGCGAGGTTCTACTAGCTCCCTG	interferon alpha inducible protein 27
IFI35	NM_005533.4	interferon-induced protein 35;IFP35	CGATGTGGACGTTCGGGAGCTACTGCCAGGGAGTGTCATGCTGGGGTTTGCTAGGGATGGAGTGGCTCAGCGTCTGTGCCAAATCGGCCAGTTCACAGTG	interferon induced protein 35
IFI6	NM_002038.3	G1P3;interferon, alpha-inducible protein (clone IFI-6-16);IFI616,FAM14C,6-16,IFI-6-16	GCAGAGCCTCGGGGCTGGTGGCAGCAGCGTCGTCATAGGTAATATTGGTGCCCTGATGGGCTACGCCACCCACAAGTATCTCGATAGTGAGGAGGATGAG	interferon alpha inducible protein 6
IFIH1	NM_022168.3	MDA-5,Hlcd,MDA5,IDDM19;helicard,melanoma differentiation-associated gene 5	GTGTGCCGACTATCAAATAAATGGTGAAATCATCTGCAAATGTGGCCAGGCTTGGGGAACAATGATGGTGCACAAAGGCTTAGATTTGCCTTGTCTCAAA	interferon induced with helicase C domain 1
IFIT1	NM_001270930.1	G10P1,IFI56,IFNAI1;interferon-induced protein with tetratricopeptide repeats 1;GARG-16	TGGTTTTAAGGAAACTTCGGAGAAAGGCATTAGATCTGGAAAGCTTGAGCCTCCTTGGGTTCGTCTACAAATTGGAAGGAAATATGAATGAAGCCCTGGA	interferon induced protein with tetratricopeptide repeats 1
IFIT2	NM_001547.4	IFI54,G10P2;interferon-induced protein with tetratricopeptide repeats 2;IFI-54,ISG-54K,cig42,GARG-39	TGCATCCCATAGAGGTTAGTCCTGCATAGCCAGTAATGTGCTAAGTTCATCCAAAAGCTGGCGGACCAAAGTCTAAATAGGGCTCAGTATCCCCCATCGC	interferon induced protein with tetratricopeptide repeats 2
IFI	NM_0	IFIT4;interferon-induced protein with tetratricopeptide repeats 4,interferon-induced	AGTTCTCTACTGATGTTTTGAAGCAGGCCATTGAGC	interferon induced protein with tetratricopeptide repeats 3

T3	01031683.3	protein with tetratricopeptide repeats 3;ISG60,RIG-G,CIG-49,IFI60,GARG-49,IRG2	TGAGTCCTGATAACCAATACGTCAAGGTTCTCTTGGGCCTGAAACTGCAGAAGATGAATAAAGA	
IFITM1	NM_003641.3	IFI17;interferon induced transmembrane protein 1 (9-27);9-27,CD225,DSPA2a;interferon-induced transmembrane protein 1,dispanin subfamily A member 2a	ACAGGAAAAACGGGGTTACTAGTAGCCGCCCATAGCCTGCAACCTTTGCACTCCACTGTGCAATGCTGGCCCTGCACGCTGGGGCTGTTGCCCCTGCCCC	interferon induced transmembrane protein 1
IFITM2	NM_006435.2	interferon induced transmembrane protein 2 (1-8D);1-8D,DSPA2c;dispanin subfamily A member 2c	CCAAGTGCCTGAACATCTGGGCCCTGATTTTGGGCATCTTCATGACCATTCTGCTCATCATCATCCCAGTGTTGGTCGTCCAGGCCCAGCGATAGATCAG	interferon induced transmembrane protein 2
IFNA1	NM_024013.1	IFNA@,IFL,IFN,IFN-ALPHA,IFNA13,IFN-alphaD;IFN-alpha 1b,interferon alpha 1b	TGACTCATACACCAGGTCACGCTTTCATGAATTCTGTCATTTCAAAGACTCTCACCCCTGCTATAACTATGACCATGCTGATAAACTGATTTATCTATTT	interferon alpha 1
IFNAR1	NM_000629.2	IFNAR;interferon (alpha, beta and omega) receptor 1;IFRC	AGCAGAAAAAGAAAACACTTCTTCATGGTATGAGGTTGACTCATTTACACCATTTCGCAAAGCTCAGATTGGTCCTCCAGAAGTACATTTAGAAGCTGAA	interferon alpha and beta receptor subunit 1
IFNG	NM_000619.2		AAAAAGAAACGAGATGACTTCGAAAAGCTGACTAATTTATTCGGTAACTGACTTGAATGTCCAACGCAAAGCAATACATGAACTCATCCAAGTGATGGCTG	interferon gamma
IFNGR1	NM_000416.2	IFNGR;CD119	TCACGTCATACCAGCCATTTTCCTTAGAAAAGGAGGTGGTCTGTGAAGAGCCGTTGTCTCCAGCAACAGTTCCAGGCATGCATACCGAAGACAATCCAGG	interferon gamma receptor 1

IFNGR2	NM_005534.3	IFNGT1;interferon gamma receptor 2 (interferon gamma transducer 1);AF-1	TCTGATACTTTTTTCATTATTGGTTGGGCTGAGCAGTCAGAAGACCTGGTCGTCGTCTTGACTTTGGCAAATGAGCCGGAGCCCCTTGGGCAGGTCACAC	interferon gamma receptor 2
IGF2R	NM_000876.2	insulin-like growth factor 2 receptor;CD222,MPRI,MPR1,CIMPR,M6P-R,CI-M6PR,CI-MPR,MPR300;cation-independent mannose-6 phosphate receptor	AGGCATGGCTCAGATCGGCCACAGGGCGGTACCTTGTGCCCAGGGTTTTGCCCCAAGTCCTCATTTAAAAGCATAAGGCCGGACGCATCTCAAAACAGAG	insulin like growth factor 2 receptor
IHH	NM_002181.2	Indian hedgehog (Drosophila) homolog;HHG2,BDA1	GGCAGCCATCCCGGCCATTCTGAGGTATGACATTCCTCCCCGGCCACACTCCTCAAGACACATCCAGAGACTGTTGCTGTCTGTGGGCAGAGTTCTGTGT	Indian hedgehog signaling molecule
IKBKB	NM_001190720.2	inhibitor of kappa light polypeptide gene enhancer in B-cells, kinase beta;IKK2,NFKBIKB,IKK-beta,IKKB	CGCAGCTCAGTAAAACTGTGGTTTGCAAGCAGAAGGCGCTGGAACTGTTGCCCAAGGTGGAAGAGGTGGTGAGCTTAATGAATGAGGATGAGAAGACTGT	inhibitor of nuclear factor kappa B kinase subunit beta
IKBKG	NM_001099856.4	IP2,IP1;incontinentia pigmenti,inhibitor of kappa light polypeptide gene enhancer in B-cells, kinase gamma,inhibitor of nuclear factor kappa B kinase subunit gamma;IKK-gamma,NEMO,Fip3p,FIP-3,FIP3,ZC2HC9	TTTTGTTCCCTTCTGTCTGCTCGAACCACTTGCCTCGGGCTAATCCCTCCCTCTTCCTCCACCCGGCACTGGGGAAGTCAAGAATGGGGCCTGGGGCTCT	inhibitor of nuclear factor kappa B kinase regulatory subunit gamma
IL10	NM_000572.2	CSIF,TGIF,IL10A,IL-10;cytokine synthesis inhibitory factor,T-cell growth inhibitory factor	TGGACAACTTGTTGTTAAAGGAGTCCTTGCTGGAGGACTTTAAGGGTTACCTGGGTTGCCAAGCCTTGTCTGAGATGATCCAGTTTTACCTGGAGGAGGT	interleukin 10
IL10	NM_00	IL10R;interleukin 10 receptor, alpha;HIL-10R,CDW210A,CD210a,CD210	TGCCCAGCCCTCCGTCTGTGTGGTTTGAAGCAGAATTTTTCCACCACAT	interleukin 10 receptor subunit alpha

RA	1558.2		CCTCCACTGGACACCCATCCCAAATCAGTCTGAAAGTACCTGCTATGAAGT	
IL11	NM_000641.3	IL-11,AGIF;adipogenesis inhibitory factor,oprelvekin	TAATGGAAGGTTCCACAAGTCACCCTGTGATCAACAGTACCCGTATGGGACAAAGCTGCAAGGTCAAGATGGTTCATTATGGCTGTGTTCACCATAGCAA	interleukin 11
IL11RA	NM_147162.1	interleukin 11 receptor, alpha	TCCAGCCCGCCCTGTTGTCTCCTGCCAAGCAGCCGACTATGAGAACTTCTCTTGCACTTGGAGTCCCAGCCAGATCAGCGGTTTACCCACCCGCTACCTC	interleukin 11 receptor subunit alpha
IL12RB2	NM_001319233.1	interleukin 12 receptor, beta 2	TTGAAGCCCAGACAAGGCTGCTTTCACTATTCCAGACGTAACAAGTTAATCCTGTACAAGTTTGACAGAAGAATCAATTTTCACCATGGCCACTCCCTCA	interleukin 12 receptor subunit beta 2
IL15	NM_000585.4	IL-15,MGC9721	GATCATCCTAGCAAACAACAGTTTGTCTTCTAATGGGAATGTAACAGAATCTGGATGCAAAGAATGTGAGGAACTGGAGGAAAAAAATATTAAAGAATTT	interleukin 15
IL16	NM_004513.4	interleukin 16 (lymphocyte chemoattractant factor);LCF,IL-16,prIL-16,HsT19289,FLJ42735,FLJ16806;prointerleukin 16,lymphocyte chemoattractant factor	GGCATCTCCAACATCATCATCCAACGAAGACTCAGCTGCAAATGGTTCTGCTGAAACATCTGCCTTGGACACAGGGTTCTCGCTCAACCTTTCAGAGCTG	interleukin 16
IL17A	NM_002190.2	CTLA8,IL17;interleukin 17 (cytotoxic T-lymphocyte-associated serine esterase 8);IL-17A,IL-17;cytotoxic T-lymphocyte-associated protein 8	GTCAACCTGAACATCCATAACCGGAATACCAATACCAATCCCAAAAGGTCCTCAGATTACTACAACCGATCCACCTCACCTTGGAATCTCCACCGCAATG	interleukin 17A

IL18	NM_001243211.1	interleukin 18 (interferon-gamma-inducing factor);IGIF,IL1F4,IL-1g,IL-18;interferon-gamma-inducing factor	ATCGGCCTCTATTTGAAGATATGACTGATTCTGACTGTAGAGATAATGCACCCCGGACCATATTTATTATAAGTATGTATAAAGATAGCCAGCCTAGAGG	interleukin 18
IL18R1	NM_001282399.1	IL1RRP,IL-1Rrp,CD218a	ATGAAGAGGATGTAATTTATTGGATGTTCGGGGAAGAAAATGGATCGGATCCTAATATACATGAAGAGAAAGAAATGAGAATTATGACTCCAGAAGGCAA	interleukin 18 receptor 1
IL1A	NM_000575.3	IL1;IL1F1,IL-1A,IL1-ALPHA;preinterleukin 1 alpha,hematopoietin-1,pro-interleukin-1-alpha	TTATTCTTCATAACAATTTTAGGAGGACCAGAGCTACTGACTATGGCTACCAAAAAGACTCTACCCATATTACAGATGGGCAAATTAAGGCATAAGAAAA	interleukin 1 alpha
IL1B	NM_000576.2	IL1F2,IL-1B,IL1-BETA	CCAGGCCTCTCTCACCTCTCCTACTCACTTAAAGCCCGCCTGACAGAAACCACGGCCACATTTGGTTCTAAGAAACCCTCTGTCATTCGCTCCCACATTC	interleukin 1 beta
IL1R2	NR_048564.1	IL1RB;interleukin 1 receptor, type II;CD121b	CCATATCAGCTTCTCTGGGGTCAAGACTGACAATCCCGTGTAAGGTGTTTCTGGGAACCGGCACACCCTTAACCACCATGCTGTGGTGGACGGCCAATGA	interleukin 1 receptor type 2
IL1RN	NM_000577.3	IL1RA,ICIL-1RA,IL1F3,IRAP,IL-1RN,MGC10430;interleukin-1 receptor antagonist protein,intracellular interleukin-1 receptor antagonist	GAGTCTGCCGCCTGCCCCGGTTGGTTCCTCTGCACAGCGATGGAAGCTGACCAGCCCGTCAGCCTCACCAATATGCCTGACGAAGGCGTCATGGTCACCA	interleukin 1 receptor antagonist
IL2	NM_00	IL-2,TCGF;T cell growth factor	AGGATGCAACTCCTGTCTTGCATTGCACTAAGTCTTGCACTTGTCACA	interleukin 2

	0586.2		AACAGTGCACCTACTTCAAGTTCTACAAAGAAAACACAGCTACAACTGGAGC	
IL21R	NM_021798.3	CD360	TCCTACCAGGGGACCTGGAGTGAATGGAGTGACCCGGTCATCTTTCAGACCCAGTCAGAGGAGTTAAAGGAAGGCTGGAACCCTCACCTGCTGCTTCTCC	interleukin 21 receptor
IL22RA1	NM_021258.2	IL22R;interleukin 22 receptor,interleukin 22 receptor, alpha 1;CRF2-9	GACGGGTACAATAACACACTGTACTGATGTCACAACTTTGCAAGCTCTGCCTTGGGTTCAGCCCATCTGGGCTCAAATTCCAGCCTCACCACTCACAAGC	interleukin 22 receptor subunit alpha 1
IL24	NM_001185158.1	ST16;mda-7,IL10B,Mob-5,C49A,FISP,IL-24;melanoma differentiation association protein 7,suppression of tumorigenicity 16 (melanoma differentiation),IL-4-induced secreted protein	CGCAAGAAAATGAGATGTTTTCCATCAGAGACAGTGCACACAGGCGGTTTCTGCTATTCCGGAGAGCATTCAAACAGTTGGACGTAGAAGCAGCTCTGAC	interleukin 24
IL2RA	NM_000417.2	IL2R,IDDM10;insulin-dependent diabetes mellitus 10,interleukin 2 receptor, alpha;CD25	AGCTCAGTCCCATCAGAGAGCGAGCGCTACCCACTTCTAAATAGCAATTTCGCCGTTGAAGAGGAAGGGCAAAACCACTAGAACTCTCCATCTTATTTTC	interleukin 2 receptor subunit alpha
IL2RB	NM_000878.2	IL15RB;interleukin 15 receptor, beta,interleukin 2 receptor, beta;CD122	GTCCTGCTGCCCGAGCCAGGAACTGTGTGTGTTGCAGGGGGGCAGTAACTCCCCAACTCCCTCGTTAATCACAGGATCCCACGAATTTAGGCTCAGAAGC	interleukin 2 receptor subunit beta
IL2RG	NM_000206.1	SCIDX1,IMD4,CIDX;severe combined immunodeficiency,combined immunodeficiency, X-linked,interleukin 2 receptor, gamma;CD132	CCACAGCTGGACTGAACAATCAGTGGATTATAGACATAAGTTCTCCTTGCCTAGTGTGGATGGGCAGAAACGCTACACGTTTCGTGTTCGGAGCCGCTTT	interleukin 2 receptor subunit gamma
IL	NM	NK4,TAIF,TAIFb,TAIFd;natural killer cell transcript 4	TTCAAAGAGGGCTACCTGGAGACA	interleukin 32

32	_0042221.4		GTGGCGGCTTATTATGAGGAGCAGCACCCAGAGCTCACTCCTCTACTTGAAAAAGAAAGAGATGGATTACGGTGCC	
IL33	NM_033439.3	C9orf26;chromosome 9 open reading frame 26 (NF-HEV);DVS27,DKFZp586H0523,NF-HEV,IL1F11;DVS27-related protein,nuclear factor for high endothelial venules,interleukin-1 family, member 11	TTTCAGTAACTGACTGTCCCTCATGTCCATGGCCTACCATCCCTTCTGACCCTGGCTTCCAGGGACCTATGTCTTTTAATACTCACTGTCACATTGGGCA	interleukin 33
IL34	NM_001172771.1	C16orf77;chromosome 16 open reading frame 77;MGC34647,IL-34	GTTTTTCCTTTGAGGGGGATTCTGTGCCACAGCAGGGCTCAGCTTCCTGCCTTCCATAGCTGTCATGGCCTCACCTGGAGCGGAGGGGACCTGGGGACCT	interleukin 34
IL4	NM_172348.1	BSF1,IL-4,BCGF1,BCGF-1,MGC79402;B_cell stimulatory factor 1,lymphocyte stimulatory factor 1,B cell growth factor 1	GCTGCGACTGTGCTCCGGCAGTTCTACAGCCACCATGAGAAGGACACTCGCTGCCTGGGTGCGACTGCACAGCAGTTCCACAGGCACAAGCAGCTGATCC	interleukin 4
IL6	NM_000600.3	IFNB2;interleukin 6 (interferon, beta 2);IL-6,BSF2,HGF,HSF;interferon, beta 2	GGTATACCTAGAGTACCTCCAGAACAGATTTGAGAGTAGTGAGGAACAAGCCAGAGCTGTGCAGATGAGTACAAAAGTCCTGATCCAGTTCCTGCAGAAA	interleukin 6
IL6R	NM_000565.3	CD126	GGAGCACCCCATCCCTGACGACAAAGGCTGTGCTCTTGGTGAGGAAGTTTCAGAACAGTCCGGCCGAAGACTTCCAGGAGCCGTGCCAGTATTCCCAGGA	interleukin 6 receptor
IL7R	NM_002185.3	CD127,IL7RA	CATTTTCTCTCCAATCTGGAATCCTGACATTGAACCCAGTTGCTCAGGGTCAGCCCATTCTTACTTCCCTGGGATCAAATCAAGAAGAAGCATATGTCAC	interleukin 7 receptor

INHBA	NM_002192.2	inhibin, beta A (activin A, activin AB alpha polypeptide),inhibin, beta A	TAGAACAACCCAAATAGCATCTAGAAAGCCATGAGTTTGAAAGGGCCCATCACAGGCACTTTCCTACCCAATTACCCAGGTCATAAGGTATGTCTGTGTG	inhibin subunit beta A
IRF1	NM_002198.2	MAR;interferon regulatory factor-1	GTACCGGATGCTTCCACCTCTCACCAAGAACCAGAGAAAAGAAAGAAAGTCGAAGTCCAGCCGAGATGCTAAGAGCAAGGCCAAGAGGAAGTCATGTGGG	interferon regulatory factor 1
IRF2	NM_002199.3		AGGCAGCACTAGCGACATTGCAGTCTGCTTCTGCACCTTATCTTAAAGCACTTACAGATAGGCCTTCTTGTGATCTTGCTCTATCTCACAGCACACTCAG	interferon regulatory factor 2
IRF3	NM_001197122.1		CAGCCAGACACCTCTCCGGACACCAATGGTGGAGGCAGTACTTCTGATACCCAGGAAGACATTCTGGATGAGTTACTGGGTAACATGGTGTTGGCCCCAC	interferon regulatory factor 3
IRF4	NM_002460.1	MUM1;LSIRF	GGGCACTGTTTAAAGGAAAGTTCCGAGAAGGCATCGACAAGCCGGACCCTCCCACCTGGAAGACGCGCCTGCGGTGCGCTTTGAACAAGAGCAATGACTT	interferon regulatory factor 4
IRF5	NM_002200.3		GCCTGGCTCTCGGGAAATTCAGCCATGAGCAGGGAAAGAACTCTCCCAACCCTGGGGCCTAGCTGTATAGGAGGAATTGCCTAAGGGTGGCCCACTCTTG	interferon regulatory factor 5
IRF7	NM_004029.2		AGCAGCAGCCTCAGCCTCTGCCTGTCCAGCGCCAACAGCCTCTATGACGACATCGAGTGCTTCCTTATGGAGCTGGAGCAGCCC	interferon regulatory factor 7

	9.2		GCCTAGAACCCAGTCT	
IRF8	NM_002163.2	ICSBP1;interferon consensus sequence binding protein 1;IRF-8,ICSBP	CCGCCGCCAGACCAGGTCTTCCGGATGTTTCCAGATATTTGTGCCTCACACCAGAGATCATTTTTCAGAGAAAACCAACAGATCACCGTCTAAGTGCGTC	interferon regulatory factor 8
IRF9	NM_006084.4	ISGF3G;interferon-stimulated transcription factor 3, gamma (48kD),interferon-stimulated transcription factor 3, gamma 48kDa	CCCCTTTCAAGGGGATCAGAGGTCCCTGGAGTTTCTGCTTCCTCCAGAGCCAGACTACTCACTGCTGCTCACCTTCATCTACAACGGGCGCGTGGTGGGC	interferon regulatory factor 9
ISG15	NM_005101.3	G1P2;interferon, alpha-inducible protein (clone IFI-15K);IFI15,UCRP	CCCGGCAGCACGGTCCTGCTGGTGGTGGACAAATGCGACGAACCTCTGAGCATCCTGGTGAGGAATAACAAGGGCCGCAGCAGCACCTACGAGGTACGGC	ISG15 ubiquitin like modifier
ITGA1	NM_181501.1	integrin, alpha 1;VLA1,CD49a	GTCAGCCCCACATTTCAAGTCGTGAATTCCATTGCCCCTGTACAAGAATGCAGCACTCAACTGGACATAGTCATAGTGCTGGATGGTTCCAACAGTATTT	integrin subunit alpha 1
ITGA2	NM_002203.3	CD49B;integrin, alpha 2 (CD49B, alpha 2 subunit of VLA-2 receptor);CD49b;alpha 2 subunit of VLA-2 receptor	GAGCAATTCAATATGCAAGAAAATATGCTTATTCAGCAGCTTCTGGTGGGCGACGAAGTGCTACGAAAGTAATGGTAGTTGTAACTGACGGTGAATCACA	integrin subunit alpha 2
ITGA4	NM_000885.5	CD49D;integrin, alpha 4 (antigen CD49D, alpha 4 subunit of VLA-4 receptor);CD49d;antigen CD49D,alpha 4 subunit of VLA-4 receptor	GGGCGATTTACAGATGCAGGATCGGAAAGAATCCCGGCCAGACGTGCGAACAGCTCCAGCTGGGTAGCCCTAATGGAGAACCTTGTGGAAAGACTTGTTT	integrin subunit alpha 4
ITGA6	NM_001316	integrin, alpha 6;CD49f	CCACAAAAGATGGCGATGACGCCCATGAGGCTAAACTGATTGCAACGTTTCCAGACACTTTAACCTATTCTGCATATAGAGAACT	integrin subunit alpha 6

	306.1		GAGGGCTTTCCCTGA	
ITGAE	NM_002208.4	integrin, alpha E (antigen CD103, human mucosal lymphocyte antigen 1; alpha polypeptide);CD103,HUMINAE;antigen CD103,human mucosal lymphocyte antigen 1, alpha polypeptide	ATCCACTGGGAGAGGCTATCAGCCAGTCCTGGGACTTGGAGACCCAGCATCCTTTGCATTACTTTTTCCTTCAGGATGATCTAGAGCAGCATGGAGCTGT	integrin subunit alpha E
ITGAL	NM_002209.2	CD11A;integrin, alpha L (antigen CD11A (p180), lymphocyte function-associated antigen 1; alpha polypeptide);LFA-1;antigen CD11A (p180),lymphocyte function-associated antigen 1, alpha polypeptide	GTGAGGGCTTGTCATTACCAGACGGTTCACCAGCCTCTCTTGGTTTCCTTCCTTGGAAGAGAATGTCTGATCTAAATGTGGAGAAACTGTAGTCTCAGGA	integrin subunit alpha L
ITGAM	NM_000632.3	CR3A,CD11B;integrin, alpha M (complement component receptor 3, alpha; also known as CD11b (p170), macrophage antigen alpha polypeptide),integrin, alpha M (complement component 3 receptor 3 subunit);MAC-1,CD11b;complement component 3 receptor 3 subunit	TTCAGGCAGAACACTGGCATGTGGGAGTCCAACGCTAATGTCAAGGGCACCCAGATCGGCGCCTACTTCGGGGCCTCCCTCTGCTCCGTGGACGTGGACA	integrin subunit alpha M
ITGAV	NM_001144999.2	VNRA,MSK8,VTNR;antigen identified by monoclonal antibody L230,vitronectin receptor,integrin, alpha V (vitronectin receptor, alpha polypeptide, antigen CD51),integrin, alpha V;CD51	TGTGGAGTTGCTCAGTGCTTGAAGATTGTCTGCCAAGTTGGGAGATTAGACAGAGGAAAGAGTGCAATCTTGTACGTAAAGTCATTACTGTGGACTGAGA	integrin subunit alpha V
ITGAX	NM_000887.4	CD11C;integrin, alpha X (antigen CD11C (p150), alpha polypeptide),integrin, alpha X (complement component 3 receptor 4 subunit);CD11c;complement component 3 receptor 4 subunit	ACAGGAGCAGGACATTGTGTTCCTGATCGATGGCTCAGGCAGCATCTCCTCCCGCAACTTTGCCACGATGATGAACTTCGTGAGAGCTGTGATAAGCCAG	integrin subunit alpha X
ITGB2	NM_000211.3	CD18,MFI7;integrin, beta 2 (antigen CD18 (p95), lymphocyte function-associated antigen 1; macrophage antigen 1 (mac-1) beta subunit),integrin, beta 2 (complement component 3 receptor 3 and 4 subunit);LFA-1,MAC-1;complement component 3 receptor 3 and 4 subunit	AACCAGCCCAGAGGTGACTGTGATGGCGTGCAGATCAATGTCCCGATCACCTTCCAGGTGAAGGTCACGGCCACAGAGTGCATCCAGGAGCAGTCGTTTG	integrin subunit beta 2
ITG	NM_0	GP3A;integrin, beta 3 (platelet glycoprotein IIIa, antigen CD61);CD61,GPIIIa;platelet glycoprotein IIIa,antigen CD61	GAATAAGCCTTGGAATTAGATATGGGGCAATGACTG	integrin subunit beta 3

B<br>3	0<br>0<br>2<br>1<br>2.<br>2		AGCCCTGTCTCA<br>CCCATGGATTAC<br>TCCTTACTGTAG<br>GGAATGGCAGTA<br>TGGTAGAGGGAT<br>AAAT	
I<br>T<br>G<br>B<br>8	N<br>M<br>_0<br>0<br>2<br>2<br>1<br>4.<br>2	integrin, beta 8	GGAAAACTGGA<br>ATTGTATGCAAT<br>GCCTTCACCCTCA<br>CAATTTGTCTCA<br>GGCTATACTTGA<br>TCAGTGCAAAAC<br>CTCATGTGCTCTC<br>ATGGAACAACAG<br>CAT	integrin<br>subunit<br>beta 8
I<br>T<br>P<br>K<br>1	N<br>M<br>_0<br>0<br>1<br>1<br>4<br>2<br>5<br>9<br>3.<br>2	inositol 1,3,4-triphosphate 5/6 kinase	GAGCTCTCCCGG<br>GCCCTGCGGCAG<br>GCACTGGGCGTG<br>TCACTCTTCGGC<br>ATCGACATCATC<br>ATCAACAACCAG<br>ACAGGGCAGCAC<br>GCCGTCATTGAC<br>ATCA	inositol-tetrakisphosphate 1-<br>kinase
J<br>A<br>G<br>1	N<br>M<br>_0<br>0<br>0<br>2<br>1<br>4.<br>2	AGS,JAGL1;Alagille syndrome,jagged<br>1;AHD,AWS,HJ1,CD339	CTACATCGCTTG<br>CGAGCCTTCCCC<br>TTCAGCGAACAA<br>TGAAATACATGT<br>GGCCATTTCTGC<br>TGAAGATATACG<br>GGATGATGGGA<br>ACCCGATCAAGG<br>AAATC	jagged canonical Notch ligand 1
J<br>A<br>G<br>2	N<br>M<br>_0<br>0<br>2<br>2<br>2<br>6.<br>4	jagged 2	ATTCAGAGGCAA<br>GGTCAGCATTTG<br>ATGGAGGCAGG<br>AGAGAAGCAGC<br>CCTTCCACCTGG<br>CCCTGGAGCTGG<br>TTTTGCCCCTCAC<br>TGCTTGAGCCAA<br>CTGGA	jagged canonical Notch ligand 2
J<br>A<br>K<br>1	N<br>M<br>_0<br>0<br>2<br>2<br>2<br>7.<br>1	JAK1B;JAK1A,JTK3	GAGAACACCAAG<br>CTCTGGTATGCT<br>CCAAATCGCACC<br>ATCACCGTTGAT<br>GACAAGATGTCC<br>CTCCGGCTCCAC<br>TACCGGATGAGG<br>TTCTATTTCACCA<br>ATT	Janus<br>kinase 1
J<br>A<br>K<br>2	N<br>M<br>_0<br>0<br>4<br>9<br>7<br>2.<br>2	JTK10	CTCCTCCCGCGA<br>CGGCAAATGTTC<br>TGAAAAAGACTC<br>TGCATGGGAATG<br>GCCTGCCTTACG<br>ATGACAGAAATG<br>GAGGGAACATCC<br>ACCTCTTCTATAT<br>ATC	Janus<br>kinase 2

JAK3	NM_000215.2	L-JAK,JAKL,LJAK,JAK3_HUMAN,JAK-3;tyrosine-protein kinase JAK3,leukocyte Janus kinase	GTGCTGCTGAAGGTCATGGATGCCAAGCACAAGAACTGCATGGAGTCATTCCTGGAAGCAGCGAGCTTGATGAGCCAAGTGTCGTACCGGCATCTCGTGC	Janus kinase 3	
KAT2B	NM_003884.4	PCAF;p300/CBP-associated factor,K(lysine) acetyltransferase 2B;P/CAF,GCN5,GCN5L	TGAGTTGGCAAAAATGTTCCTAAACCGCATCAACTATTGGCATCTGGAGGCACCATCTCAACGAAGACTGCGATCTCCCAATGATGATATTTCTGGATAC	lysine acetyltransferase 2B	
KDR	NM_002253.2	kinase insert domain receptor (a type III receptor tyrosine kinase);FLK1,VEGFR,VEGFR2,CD309;vascular endothelial growth factor receptor 2,fetal liver kinase 1	GGCTCTGCGTGGAGACCCGGGCCGCCTCTGTGGGTTTGCCTAGTGTTTCTCTTGATCTGCCCAGGCTCAGCATACAAAAAGACATACTTACAATTAAGGC	kinase insert domain receptor	
KIF2C	NM_006845.2	KNSL6;kinesin-like 6 (mitotic centromere-associated kinesin);MCAK,CT139	GTTGTCTACAGGTTCACAGCAAGGCCACTGGTACAGACAATCTTTGAAGGTGGAAAAGCAACTTGTTTTGCATATGGCCAGACAGGAAGTGGCAAGACAC	kinesin family member 2C	
KIR2DL3	NM_015868.2	killer cell immunoglobulin-like receptor, two domains, long cytoplasmic tail, 3;cl-6,nkat2,nkat2a,nkat2b,p58,CD158B2	CCCCTGGTGAAATCAGAAGAGACAGTCATCCTGCAATGTTGGTCAGATGTCAGGTTTCAGCACTTCCTTCTGCACAGAGAAGGGAAGTTTAAGGACACTT	killer cell immunoglobulin like receptor, two Ig domains and long cytoplasmic tail 3	KIR2DS2 (NM_001291701;NM_012312;NM_001291695), KIR2DL2 (NM_014219)
KIR3DL1	NM_001322168.1	KIR;killer cell immunoglobulin-like receptor, three domains, long cytoplasmic tail, 1;cl-2,NKB1,cl-11,nkat3,NKB1B,AMB11,CD158e1/2,CD158E1,CD158e2	AATCAGGAGAGAGAGTCATCCTGCAATGTTGGTCAGATATCATGTTTGAGCACTTCTTTCTGCACAAAGAGGGGATCTCTAAGGACCCCTCACGCCTCGT	killer cell immunoglobulin like receptor, three Ig domains and long cytoplasmic tail 1	KIR3DS1 (NM_001282171;NM_001282170;NM_001083539), KIR3DL2 (NM_001242867;NM_006737), LOC112268355 (NM_001368254)
KIR3D	NM_0067	killer cell immunoglobulin-like receptor, three domains, long cytoplasmic tail, 2;cl-5,nkat4,nkat4a,nkat4b,CD158K	TGCCACCCACGGAGGGACCTACAGATGCTTCGGCTCTTTCCGTGCCCTGCCCTGCGTGTGGTCAAACTCAAG	killer cell immunoglobulin like receptor, three Ig domains and long cytoplasmic tail 2	

L2	37.2		TGACCCACTGCTTGTTTCTGTCACAGGA	
KIT	NM_000222.2	PBT;piebald trait,v-kit Hardy-Zuckerman 4 feline sarcoma viral oncogene homolog;CD117,SCFR,C-Kit;mast/stem cell growth factor receptor Kit	CTAGTTCTGTGTACTCAACGTGGAAAAGAGAAAACAGTCAGACTAAACTACAGGAGAAATATAATAGCTGGCATCACGGTGACTTCAATTATGAACGTCA	KIT proto-oncogene, receptor tyrosine kinase
KLRB1	NM_002258.2	NKR;killer cell lectin-like receptor subfamily B, member 1;CD161,NKR-P1,NKR-P1A,hNKR-P1A,CLEC5B;natural killer cell surface protein P1A	TGAGTTAAACTTACCCACAGACTCAGGCCCAGAAAGTTCTTCACCTTCATCTCTTCCTCGGGATGTCTGTCAGGGTTCACCTTGGCATCAATTTGCCCTG	killer cell lectin like receptor B1
KLRD1	NM_002262.3	CD94;killer cell lectin-like receptor subfamily D, member 1	CTCCAGGACCCAACATAGAACTCCAGAAAGACTCTGACTGCTGTTCTTGCCAAGAAAAATGGGTTGGGTACCGGTGCAACTGTTACTTCATTTCCAGTGA	killer cell lectin like receptor D1
KLRK1	NM_007360.3	D12S2489E;DNA segment on chromosome 12 (unique) 2489 expressed sequence,killer cell lectin-like receptor subfamily K, member 1;NKG2D,KLR,NKG2-D,CD314	GAGAGTAAAAACTGGTATGAGAGCCAGGCTTCTTGTATGTCTCAAAATGCCAGCCTTCTGAAAGTATACAGCAAAGAGGACCAGGATTTACTTAAACTGG	killer cell lectin like receptor K1
KRAS	NM_004985.3	KRAS2;v-Ki-ras2 Kirsten rat sarcoma 2 viral oncogene homolog,v-Ki-ras2 Kirsten rat sarcoma viral oncogene homolog,Kirsten rat sarcoma viral oncogene homolog;KRAS1	GCATGGACTGTGTCCCCACGGTCATCCAGTGTTGTCATGCATTGGTTAGTCAAAATGGGGAGGGACTAGGGCAGTTTGGATAGCTCAACAAGATACAATC	KRAS proto-oncogene, GTPase
LAG3	NM_002286.5	lymphocyte-activation gene 3;CD223	TTTTGGTGACTGGAGCCTTTGGCTTTCACCTTTGGAGAAGACAGTGGCGACCAAGACGATTTTCTGCCTTAGAGCAAGGGATTCACCCTCCGCAGGCTCA	lymphocyte activating 3
LAIR1	NM_00128	leukocyte-associated Ig-like receptor 1,leukocyte-associated immunoglobulin-like receptor 1;CD305	GCAGAAGCCACAGCAGAGGCCTGACCTGGCTGTTGATGTTCTAGAGAGGACAGCAGACAAGGCCACAGTCAATGGACTTCCTGA	leukocyte associated immunoglobulin like receptor 1

	9023.2		GAAGGACAGAGAGACG	
LAMA1	NM_005559.3	LAMA;laminin, alpha 1	CTGTGCTCTGTGATGGAAAATGGCACACTCTTCAAGCTAACAAAAGCAAACACCGTATCACTCTGATTGTTGACGGGAACGCAGTTGGCGCTGAAAGTCC	laminin subunit alpha 1
LAMB3	NM_000228.2	LAMNB1;laminin, beta 3 (nicein (125kD), kalinin (140kD), BM600 (125kD)),laminin, beta 3;nicein-125kDa,kalinin-140kDa,BM600-125kDa	GCAGGCATTGAGTGCCCAAGAGGGATTTGAGAGAATAAAACAAAAGTATGCTGAGTTGAAGGACCGGTTGGGTCAGAGTTCCATGCTGGGTGAGCAGGGT	laminin subunit beta 3
LAMC2	NM_005562.2	EBR2,LAMB2T,LAMNB2,EBR2A;laminin, gamma 2 (nicein (100kD), kalinin (105kD), BM600 (100kD), Herlitz junctional epidermolysis bullosa)),laminin, gamma 2;nicein-100kDa,kalinin-105kDa,BM600-100kDa	TGTGTTTAGCTCAGCCCAACGACTAGACCCTGTCTATTTTGTGGCTCCTGCCAAATTTCTTGGGAATCAACAGGTGAGCTATGGTCAAAGCCTGTCCTTT	laminin subunit gamma 2
LCK	NM_005356.4	lymphocyte-specific protein tyrosine kinase	GATGGAGTTCTTGTGCCATAGTCACATGGCCTATGCACATATGGACTCTGCACATGAATCCCACCCACATGTGACACATATGCACCTTGTGTCTGTACAC	LCK proto-oncogene, Src family tyrosine kinase
LDHA	NM_001135239.1		GTGCAGATACACTTTGGGGGATCCAAAAGGAGCTGCAATTTTAAAGTCTTCTGATGTCATATCATTTCACTGTCTAGGCTACAACAGGATTCTAGGTGGA	lactate dehydrogenase A
LDHB	NM_0011740 9		CTGAGCCTTCCATGTATCCTCAATGCCCGGGGATTAACCAGCGTTATCAACCAGAAGCTAAAGGATGATGAGGTTGCTCAGCTCAAGAAAAGTGCAGATA	lactate dehydrogenase B

	7.2				
LGALS9	NM_002308.3	lectin, galactoside-binding, soluble, 9;LGALS9A	AGAGGCCATGTCCTTGTCTGGTCCTGCTTCTGGCTACAGCCACCCTGGAACGGAGAAGGCAGCTGACGGGGATTGCCTTCCTCAGCCGCAGCAGCACCTG	galectin 9	LGALS9C (NM_001040078), LGALS9B (NM_001367292;NM_001042685)
LIF	NM_002309.4	leukemia inhibitory factor,LIF, interleukin 6 family cytokine;CDF,DIA,HILDA;differentiation inhibitory activity,differentiation-inducing factor,hepatocyte-stimulating factor III,cholinergic differentiation factor,human interleukin in DA cells	CCCACCGTGCGGCCTTAGAGGTTTCCCTCCTTCCTTTCCACTGAAAAGCACATGGCCTTGGGTGACAAATTCCTCTTTGATGAATGTACCCTGTGGGGAT	LIF interleukin 6 family cytokine	
LILRA1	NM_001278318.1	leukocyte immunoglobulin-like receptor, subfamily A (with TM domain), member 1;LIR-6,CD85i,LIR6;leucocyte Ig-like receptor A1	TGAAGAAGGGCCAGTTCCCCATCCCATCCATCACCTGGGAACACACAGGGCGGTATCGCTGTTTCTACGGTAGCCACACTGCAGGCTGGTCAGAGCCCAG	leukocyte immunoglobulin like receptor A1	
LILRA3	NM_001172654.2	leukocyte immunoglobulin-like receptor, subfamily A (without TM domain), member 3;LIR-4,HM43,ILT6,HM31,LIR4,CD85e;leucocyte Ig-like receptor A3	GTGTCAGTCACAGGGAGGGATGCACACTTTCCTTTTGACCAAGGAGGGGGCAGCTGATTCCCCGCTGCGTCTAAAATCAAAGCGCCAATCTCATAAGTAC	leukocyte immunoglobulin like receptor A3	
LILRA5	NM_181879.2	LILRB7;leukocyte immunoglobulin-like receptor, subfamily A (with TM domain), member 5;ILT11,LIR9,CD85,CD85f;leucocyte Ig-like receptor A5	CACCCTCTCAGCCCTGCCCAGTCCTGTGGTGACCTCAGGAGAGAACGTGACCCTCCAGTGTGGCTCACGGCTGAGATTCGACAGGTTCATTCTGACTGAG	leukocyte immunoglobulin like receptor A5	
LILRB2	NM_001278840	leukocyte immunoglobulin-like receptor, subfamily B (with TM and ITIM domains), member 2;LIR-2,ILT4,MIR-10,LIR2,CD85d,MIR10;myeloid inhibitory receptor 10,leucocyte Ig-like receptor B2	GATACGACCAGAGCTTGTGAAGAACGGCCAGTTCCACATCCCATCCATCACCTGGGAACACACAGGGCGATATGGCTGTCAGTATTACAGCCGCGCTCGG	leukocyte immunoglobulin like receptor B2	

	5.1			
LILRB4	NM_001278426.3	leukocyte immunoglobulin-like receptor, subfamily B (with TM and ITIM domains), member 4;LIR-5,ILT3,HM18,LIR5,CD85k;leucocyte Ig-like receptor B4	TTTCAGCCCTGCCGAGTCCTCTTGTGACCTCAGGAAAGAGCGTGACCCTGCTGTGTCAGTCACGGAGCCCAATGGACACTTTTCTTCTGATCAAGGAGCG	leukocyte immunoglobulin like receptor B4
LOXL2	NM_002318.2	lysyl oxidase-like 2;WS9-14,LOR	GGTTTGCCATCCTCCTCTAGTTAAAAGTAAGGGGGAAAAGAGTAAACGCGCGACTCCAGCGCGCGGCTACCTACGCTTGGTGCTTGCTTTCTCCAGCCAT	lysyl oxidase like 2
LRRC32	NM_005512.2	D11S833E,GARP;glycoprotein A repetitions predominant	GCTGTCTGGGAAGAATGGACGTGCTGGGACCAACTCAAGACCTTGTTTTGCTGTCTTCATCATCTTACCTGTGCTTGGCCCACAGTCTGGCTCATGATGT	leucine rich repeat containing 32
LTB	NM_002341.1	TNFC;p33,TNFSF3;TNF superfamily member 3	AGGAACAGGCGTTTCTGACGAGCGGGACGCAGTTCTCGGACGCCGAGGGGCTGGCGCTCCCGCAGGACGGCCTCTATTACCTCTACTGTCTCGTCGGCTA	lymphotoxin beta
LTBP1	NM_000627.3	TGF-beta1-BP-1	AAGTTTCTTGTGCATTTGCCCAGCAGGATTTATGGCCAGTGAGGAGGGTACTAACTGCATAGATGTTGACGAATGCCTGAGGCCGGACGTCTGTGGGGAG	latent transforming growth factor beta binding protein 1
LY9	NM_001033667.2	CD229,mLY9,SLAMF3,hly9	GAGAACGTCATCTGGATTGGTCCCAAAAATGCTCTTGCTTTCGCACGTCCCAAAGAAAATGTAACCATTATGGTCAAAAGCTACCTGGGCCGACTAGACA	lymphocyte antigen 9
LY	NM	MD-2	TCCACCCTGTTTTCTTCCATATTTAC	lymphocyte

96	_001195797.1		TGAAGCTCAGAAGCAGTATTGGGTCTGCAACTCATCCGATGCAAGTATTTCATACACCTACTGTGGGAGAGATT	antigen 96
LYZ	NM_000239.2	lysozyme (renal amyloidosis);renal amyloidosis	TGCTGTAGCTTGTGCAAAGAGGGTTGTCCGTGATCCACAAGGCATTAGAGCATGGGTGGCATGGAGAAATCGTTGTCAAAACAGAGATGTCCGTCAGTAT	lysozyme
MAGEA1	NM_004988.4	MAGE1;melanoma antigen family A, 1 (directs expression of antigen MZ2-E),melanoma antigen family A1;MGC9326,CT1.1;melanoma-associated antigen 1,melanoma-associated antigen MZ2-E,melanoma antigen MAGE-1,melanoma antigen family A 1,cancer/testis antigen family 1, member 1	CCCTGGAGGAGGTGCCCACTGCTGGGTCAACAGATCCTCCCCAGAGTCCTCAGGGAGCCTCCGCCTTTCCCACTACCATCAACTTCACTCGACAGAGGCA	MAGE family member A1
MAGEA12	NM_001166386.1	MAGE12;melanoma antigen family A12;CT1.12;cancer/testis antigen family 1, member 12	CCCACTACCATCAACTATACTCTCTGGAGTCAATCCGATGAGGGCTCCAGCAACGAAGAACAGGAAGGGCCAAGCACCTTTCCTGACCTGGAGACGAGCT	MAGE family member A12
MAGEA3/A6	NM_005362.3	MAGE3;melanoma antigen family A3;HYPD,HIP8,MGC14613,CT1.3;melanoma-associated antigen 3,antigen MZ2-D,MAGE-3 antigen,cancer/testis antigen family 1, member 3	AAAATCTGGGAGGAGCTGAGTGTGTTAGAGGTGTTTGAGGGGAGGGAAGACAGTATCTTGGGGGATCCCAAGAAGCTGCTCACCCAACATTTCGTGCAGG	MAGE family member A3/A6
MAGEA4	NM_001011548.1	MAGE4;melanoma antigen family A4;MAGE4A,MAGE4B,MAGE-41,MAGE-X2,MGC21336,CT1.4;melanoma-associated antigen 4,cancer/testis antigen family 1, member 4	GGCCTGCTGGGTAATAATCAGATCTTTCCCAAGACAGGCCTTCTGATAATCGTCCTGGGCACAATTGCAATGGAGGGCGACAGCGCCTCTGAGGAGGAAA	MAGE family member A4
MAG	NM_0	melanoma antigen family B2;DAM6,MAGE-XP-2,MGC26438,CT3.2;DSS/AHC critical interval MAGE	AGAAGCTTTGAAAGATGAAGAGAAAGCCGGAGTCT	MAGE family

EB2	023664.4	superfamily 6,melanoma-associated antigen B2,cancer/testis antigen family 3, member 2	GAGCCAGAGTTGTAGCCAGGCCTTGCACTACTGCCATAGCCAATCAATCTCCCAAAGCCAAGTTT	member B2
MAGEC1	NM_005462.4	melanoma antigen family C1;MAGE-C1,CT7,MGC39366,CT7.1;cancer/testis antigen family 7, member 1	TATGCCTACTGCTGGGATGCCGAGTCTTCTCCAGAGTTCCTCTGAGAGTCCTCAGAGTTGTCCTGAGGGGGAGGACTCCCAGTCTCCTCTCCAGATTCCC	MAGE family member C1
MAGEC2	NM_016249.3	MAGEE1;melanoma antigen, family E, 1, cancer/testis specific,melanoma antigen family C2;CT10,MAGE-C2,HCA587;cancer/testis antigen 10	GCTGCTCCTGAAGAAGTCGTCATGCCTCCCGTTCCAGGCGTTCCATTCCGCAACGTTGACAACGACTCCCCGACCTCAGTTGAGTTAGAAGACTGGGTAG	MAGE family member C2
MAML2	NM_032427.1	mastermind (Drosophila)-like 2,mastermind-like 2 (Drosophila);KIAA1819,MAM3	GCCACCACAGAGAACATCAAACGTAATGATCACATCCAACACAACTGCACCAAACTGGGCCTCTCAAGAAGGAACAAGCAAACAGCAAGAAGCCCTGACG	mastermind like transcriptional coactivator 2
MAP3K12	NM_006301.3	ZPK;MUK,DLK,ZPKP1,MEKK12;dual leucine zipper kinase DLK	CCTTCAGAGGTCATCCCTGGCCCTGAACCCAGCTCCCTGCCCATTCCACACCAGGAACTTCTCAGAGAGCGGGGCCCTCCCAATTCTGAGGACTCAGACT	mitogen-activated protein kinase kinase kinase 12
MAP3K5	NM_005923.3	MEKK5;MAPKKK5,ASK1;apoptosis signal regulating kinase 1	CCTGCGGAGAAAGAGATGTCAAGGGAATTCGGACACTCTTTTTGGGCATTCCAGATGAGAATTTTGAAGATCACAGTGCTCCTCCTTCCCCTGAAGAAAA	mitogen-activated protein kinase kinase kinase 5
MAP3K7	NM_003188.3	TAK1;MEKK7;TGF-beta activated kinase 1	ATTTACCTAAGCCCATTGAGAGCCTGATGACTCGTTGTTGGTCTAAAGATCCTTCCCAGCGCCCTTCAATGGAGGAAATTGTGAAAATAATGACTCACTT	mitogen-activated protein kinase kinase kinase 7
MAP3	NM_00	COT,ESTF;Tpl-2,EST,c-COT,MEKK8	AGCCAGCAGTTTATGAACCCAGTCTAATGACCATGTGTCAAGACAGTA	mitogen-activated protein kinase kinase kinase 8

K8	124413 4.1		ATCAAAACGATGAGCGTTCTAAGTCTCTGCTGCTTAGTGGCCAAGAGGTACC	
MAPK10	NM_0013180 67.1	PRKM10;JNK3,p493F12,p54bSAPK	ATATGTGATACTTATGTAAAGTGTGGGGCGAGTGGGTAACAGTTTTCAGGCACAAAATGGTTTGGCCTTCTGAAGGCAGGTGTGAATAAAAGCTGAGAGT	mitogen-activated protein kinase 10
MARCO	NM_00677 0.3	SCARA2,SR-A6;scavenger receptor class A, member 2	CTGAAGTTTACTACAGTGGTACCTGGGGGACAATTTGCGATGACGAGTGGCAAAATTCTGATGCCATTGTCTTCTGCCGCATGCTGGGTTACTCCAAAGG	macrophage receptor with collagenous structure
MB21D1	NM_13844 1.2	C6orf150,MB21D1;chromosome 6 open reading frame 150,Mab-21 domain containing 1	CTAAAGCCATTTTACCTTGTACCCAAGCATGCAAAGGAAGGAAATGGTTTCCAAGAAGAAACATGGCGGCTATCCTTCTCTCACATCGAAAAGGAAATTT	cyclic GMP-AMP synthase
MELK	NM_01479 1.2	KIAA0175	AGAGACAGCCAACAAAATATTCATGGTTCTTGAGTACTGCCCTGGAGGAGAGCTGTTTGACTATATAATTTCCCAGGATCGCCTGTCAGAAGAGGAGACC	maternal embryonic leucine zipper kinase
MET	NM_00024 5.2	met proto-oncogene;HGFR,RCCP2,DFNB97;hepatocyte growth factor receptor	CAGCGAAGTCCTCTTAACATCTATATCCACCTTCATTAAAGGAGACCTCACCATAGCTAATCTTGGGACATCAGAGGGTCGCTTCATGCAGGTTGTGGTT	MET proto-oncogene, receptor tyrosine kinase
MFGE8	NM_00111 4	SPAG10;sperm associated antigen 10,milk fat globule-EGF factor 8 protein;SED1,EDIL1,BA46,OAcGD3S,HsT19888,MFG-E8,hP47;sperm surface protein hP47,lactadherin	GCCACTGGGCCTGGAGAATGGGAACATTGCCAACTCACAGATCGCCGCCTCGTCTGTGCGTGTGACCTTCTTGGGTTTGCAGC	milk fat globule EGF and factor V/VIII domain containing

	614.1		ATTGGGTCCCGGAGCTG	
MFNG	NM_002405.3	manic fringe (Drosophila) homolog,manic fringe homolog (Drosophila)	CCCTTGCGAACAGGACCAGATTTTGTTTGGAGCCTCAGCATGCCGGGGCCCAGATGATGGAGCATAACGGGTCCCAGCCAATTGTGATGATCCTTTTTGC	MFNG O-fucosylpeptide 3-beta-N-acetylglucosaminyltransferase
MGMT	NM_002412.3	methylated-DNA--protein-cysteine methyltransferase	TATTTCCACCAGCCCGAGGCTATCGAAGAGTTCCCCGTGCCGGCTCTTCACCATCCCGTTTTCCAGCAAGAGTCGTTCACCAGACAGGTGTTATGGAAGC	O-6-methylguanine-DNA methyltransferase
MICA	NM_000247.1	PERB11.1	AAGATGCCATGAAGACCAAGACACACTATCACGCTATGCATGCAGACTGCCTGCAGGAACTACGGCGATATCTAAAATCCGGCGTAGTCCTGAGGAGAAC	MHC class I polypeptide-related sequence A
MICB	NM_005931.3	PERB11.2	TGCAAAGTGTTAGTAGGTATGAGGTGTTTGCTGCTCTGCCACGTAGAGAGCCAGCAAAGGGATCATGACCAACTCAACATTCCATTGGAGGCTATATGAT	MHC class I polypeptide-related sequence B
MKI67	NM_002417.2	antigen identified by monoclonal antibody Ki-67;MIB-1,PPP1R105;protein phosphatase 1, regulatory subunit 105	GCTTCCAGCAGCAAATCTCAGACAGAGGTTCCTAAGAGAGGAGGAGAAAGAGTGGCAACCTGCCTTCAAAAGAGAGTGTCTATCAGCCGAAGTCAACATG	marker of proliferation Ki-67
MLANA	NM_005511.1	MART1	GCACTCAATGTGCCTTAACAAGAAGATGCCCACAAGAAGGGTTTGATCATCGGGACAGCAAAGTGTCTCTTCAAGAGAAAAACTGTGAACCTGTGGTTCC	melan-A
MLH1	NM_0002	COCA2;mutL (E. coli) homolog 1 (colon cancer, nonpolyposis type 2),mutL homolog 1, colon cancer, nonpolyposis type 2 (E. coli);HNPCC,FCC2,HNPCC2	CAGGGACATGAGGTTCTCCGGGAGATGTTGCATAACCACTCCTTCGTGGGCTGTGTGAATCCTCAGTGGGC	mutL homolog 1

	49.2		CTTGGCACAGCATCAAACCAAGTTATACC	
MMP1	NM_002421.2	CLG;matrix metalloproteinase 1 (interstitial collagenase);interstitial collagenase	CAACTTACATCGTGTTGCGGCTCATGAACTCGGCCATTCTCTTGGACTCTCCCATTCTACTGATATCGGGGCTTTGATGTACCCTAGCTACACCTTCAGT	matrix metallopeptidase 1
MMP7	NM_002423.3	MPSL1;matrix metalloproteinase 7 (matrilysin, uterine);PUMP-1;matrilysin	GTTAAACTCCCGCGTCATAGAAATAATGCAGAAGCCCAGATGTGGAGTGCCAGATGTTGCAGAATACTCACTATTTCCAAATAGCCCAAAATGGACTTCC	matrix metallopeptidase 7
MMP9	NM_004994.2	CLG4B;matrix metalloproteinase 9 (gelatinase B, 92kDa gelatinase, 92kDa type IV collagenase)	TTCCAGTACCGAGAGAAAGCCTATTTCTGCCAGGACCGCTTCTACTGGCGCGTGAGTTCCCGGAGTGAGTTGAACCAGGTGGACCAAGTGGGCTACGTGA	matrix metallopeptidase 9
MMRN2	NM_024756.2	EMILIN3;elastin microfibril interfacer 3;EndoGlyx-1,FLJ13465	GCCAAGGATGGGCTGGAGGTCATTCAGTTGGTCTGTCTCTTCCCTGGAAACCTTCTGCAAAGATGGTGTGGTGTACGTGGCTTCCCTGTAACCACATGGG	multimerin 2
MRC1	NM_002438.2	MRC1L1;mannose receptor, C type 1-like 1,mannose receptor, C type 1;CLEC13D,CD206,bA541I19.1,CLEC13DL;macrophage mannose receptor	CTATGGAACCACAGACAATCTGTGCTCCAGAGGTTATGAAGCCATGTATACGCTACTAGGCAATGCCAATGGAGCAACCTGTGCATTCCCGTTCAAGTTT	mannose receptor C-type 1
MRE11	NM_001330347.1	MRE11A;MRE11 meiotic recombination 11 homolog A (S. cerevisiae),MRE11 homolog A, double strand break repair nuclease;ATLD;AT-like disease	TGTGGAAGAAGACATTTTTCCTACCACTTCAAAGACAGATCAAAGGTGGTCCAGCACATCATCCAGCAAAATCATGTCCCAGAGTCAAGTATCGAAAGGG	MRE11 homolog, double strand break repair nuclease
MS4	NM_02	CD20;membrane-spanning 4-domains, subfamily A, member 1;B1,Bp35,MS4A2	ACCCAGAAATTCAGTAAATGGGACTTTCCCGGCAGAGCCAATGAAAGG	membrane spanning 4-domains A1

A1	1950.3		CCCTATTGCTATGCAATCTGGTCCAAAACCACTCTTCAGGAGGATGTCTTCA	
MS4A2	NM_000139.3	FCER1B,IGER,APY;IgE responsiveness (atopic),membrane-spanning 4-domains, subfamily A, member 2 (Fc fragment of IgE, high affinity I, receptor for; beta polypeptide),membrane-spanning 4-domains, subfamily A, member 2;MS4A1;Fc fragment of IgE, high affinity I, receptor for; beta polypeptide	GCAAATCTTGCTCTCCCACAGGAGCCTTCCAGTGTGCCTGCATTTGAAGTCTTGGAAATATCTCCCCAGGAAGTATCTTCAGGCAGACTATTGAAGTCGG	membrane spanning 4-domains A2
MS4A4A	NM_001243266.1	MS4A4;membrane-spanning 4-domains, subfamily A, member 4,membrane-spanning 4-domains, subfamily A, member 4A;CD20L1,MS4A7	TTGAGGCCACCAAAGATCAACAGACAAATGCTCCAGAAATCTATGCTGACTGTGACACAAGAGCCTCACATGAGAAATTACCAGTATCCAACTTCGATA	membrane spanning 4-domains A4A
MS4A6A	NM_152852.2	MS4A6;membrane-spanning 4-domains, subfamily A, member 6A;CD20L3	GAACAGACTTGCCTAACAACAGGAAACTTGTATGTCTCGAAGTGGCAATTCACACATAAGGCTCCATGACTCCTGAACTCTCACAAATATTAGTTGGCTC	membrane spanning 4-domains A6A
MSH2	NM_000251.1	COCA1;mutS (E. coli) homolog 2 (colon cancer, nonpolyposis type 1),mutS homolog 2, colon cancer, nonpolyposis type 1 (E. coli);HNPCC,HNPCC1;DNA mismatch repair protein Msh2	AGGTGAAGAAAGGTGTCTGTGATCAAAGTTTTGGGATTCATGTTGCAGAGCTTGCTAATTTCCCTAAGCATGTAATAGAGTGTGCTAAACAGAAAGCCCT	mutS homolog 2
MSH6	NM_000179.2	GTBP;mutS (E. coli) homolog 6,mutS homolog 6 (E. coli)	AGGCCTGAACAGCCCTGTCAAAGTTGCTCGAAAGCGGAAGAGAATGGTGACTGGAAATGGCTCTCTTAAAAGGAAAAGCTCTAGGAAGGAAACGCCCTCA	mutS homolog 6
MTOR	NM_004958.3	FRAP,FRAP2,FRAP1;FK506 binding protein 12-rapamycin associated protein 1,mechanistic target of rapamycin (serine/threonine kinase),mechanistic target of rapamycin;RAFT1,RAPT1,FLJ44809;FK506 binding protein 12-rapamycin associated protein 2,rapamycin target protein,FKBP12-rapamycin complex-associated protein 1,FKBP-rapamycin associated protein,rapamycin associated protein FRAP2,dJ576K7.1 (FK506 binding protein 12-rapamycin associated protein 1),rapamycin and FKBP12 target 1,mammalian target of rapamycin	TGACTACCTGCATTTACTGCTGCCTCCTATTGTTAAGTTGTTTGATGCCCCTGAAGCTCCACTGCCATCTCGAAAGGCAGCGCTAGAGACTGTGGACCGC	mechanistic target of rapamycin kinase

MX1	NM_001144925.2	myxovirus (influenza) resistance 1, homolog of murine (interferon-inducible protein p78),myxovirus (influenza virus) resistance 1, interferon-inducible protein p78 (mouse);IFI-78K,MxA,lncMX1-215;interferon-inducible protein p78	GAGATTGAGATTTCGGATGCTTCAGAGGTAGAAAAGGAAATTAATAAAGCCCAGAATGCCATCGCCGGGGAAGGAATGGGAATCAGTCATGAGCTAATCA	MX dynamin like GTPase 1
MXI1	NM_001008541.1	MAX interacting protein 1,MAX interactor 1;MXD2,MAD2,MXI,bHLHc11	ATGTGTTCCCTTTGTCTTTCAAACTCCAAGGTTCCCTTGTGGCCCTCTCCCTTACCCTGGGAAGGCCTCTTGGAGACCTTACCCCTGGCTGTTTGGACTT	MAX interactor 1, dimerization protein
MYC	NM_002467.3	v-myc avian myelocytomatosis viral oncogene homolog;c-Myc,bHLHe39,MYCC	CACCGAGGAGAATGTCAAGAGGCGAACACACAACGTCTTGGAGCGCCAGAGGAGGAACGAGCTAAAACGGAGCTTTTTTGCCCTGCGTGACCAGATCCCG	MYC proto-oncogene, bHLH transcription factor
MYCT1	NM_025107.2	MTLC,FLJ21269	ATAACACAACAAGTTTAGGGAGTCCATGGCCAGAAAACTTTTGGGAGGACCTTATCATGTCCTTCACTGTATCCATGGCAATCGGGCTGGTACTTGGAGG	MYC target 1
MYD88	NM_002468.3	myeloid differentiation primary response gene (88),myeloid differentiation primary response 88	ACGTTTTTCTAGGTACAGCTCCCAGGAACAGCTAGGTGGGAAAGTCCCATCACTGAGGGAGCCTAACCATGTCCCTGAACAAAAATTGGGCACTCATCTA	MYD88 innate immune signal transduction adaptor
NBN	NM_002485.4	NBS,NBS1;Nijmegen breakage syndrome 1 (nibrin);ATV,AT-V2,AT-V1	GCGGTGATTTTCATGACTACAAAGAATTACTGTGATCCTCAGGGCCATCCCAGTACAGGATTAAAGACAACAACTCCAGGACCAAGCCTTTCACAAGGCG	nibrin
NCA	NM_0	NCAM,CD56	GAGGAAGACGATGAGAAGTACATCTTCAGCGACGATAGTTCCCAGCT	neural cell adhesion molecule 1

M1	0615.6		GACCATCAAAAAGGTGGATAAGAACGACGAGGCTGAGTACATCTGCATTGCTG	
NCR1	NM_001145457.2	LY94;lymphocyte antigen 94 (mouse) homolog (activating NK-receptor; NK-p46);NK-p46,NKP46,CD335	CACCCTCTCGGTTCATCCTGGACCCGAAGTGATCTCGGGAGAGAAGGTGACCTTCTACTGCCGTCTAGACACTGCAACAAGCATGTTCTTACTGCTCAAG	natural cytotoxicity triggering receptor 1
NDUFA4L2	NM_020142.3	NADH dehydrogenase (ubiquinone) 1 alpha subcomplex, 4-like 2,NDUFA4, mitochondrial complex associated like 2;NUOMS,FLJ26118,COXFA4L2;NADH-ubiquinone oxidoreductase MLRQ subunit homolog,cytochrome c oxidase subunit FA4 like 2	CCAGCCAGCCCATCCACTTCTTCCACTCCTCCCCGCAGGCCCCAAGGCATCACTCCGGCCACCCTGTCCCGCTACTGCTTACACAGGCCGGGTTCCCACG	NDUFA4 mitochondrial complex associated like 2
NECTIN1	NM_002855.4	HVEC,ED4,PVRL1;poliovirus receptor-related 1 (herpesvirus entry mediator C);PRR,PRR1,PVRR1,SK-12,HIgR,CLPED1,CD111,OFC7;nectin	CACCCAGGTCACATGGCAGAAGTCCACCAATGGCTCCAAGCAGAACGTGGCCATCTACAACCCATCCATGGGCGTGTCCGTGCTGGCTCCCTACCGCGAG	nectin cell adhesion molecule 1
NECTIN2	NM_001042724.1	HVEB,PVRL2;poliovirus receptor-related 2 (herpesvirus entry mediator B);PVRR2,PRR2,CD112	GCTTCGAGGAACCAGCCCTGATACCTGTGACCCTCTCTGTACGCTACCCTCCTGAAGTGTCCATCTCCGGCTATGATGACAACTGGTACCTCGGCCGTAC	nectin cell adhesion molecule 2
NEIL1	NM_024608.2	nei endonuclease VIII-like 1 (E. coli);FLJ22402,hFPG1,NEI1,FPG1;DNA endonuclease eight-like glycosylase 1	TTAGCAGGAGGCTCTCCTTGCTTGCACTCACCCTTTCTTATTGTCTTGCCCTGCATCTGGGGGTCTGAATTTTTGGGAGCAGGCAATATCTGAAGGTGCA	nei like DNA glycosylase 1
NF1	NM_00026	neurofibromatosis,von Recklinghausen disease,Watson disease	ACATGAATAAGAAGTTATTTCTGGACAGTCTACGAAAAGCTCTTGCTGGCCATGGAGGAAGTAGGCAGCTGACAGAAAGTGCT	neurofibromin 1

	7.3		GCAATTGCCTGTGTCAA	
NFAM1	NM_145912.5	CNAIP	ATGTAGTTCAATAGTTTTCTTGGCAGACAGCTTATGCAGGAGAGGTGGGCCGTTGGCTCAGTCCAGCCAGGCTGAGTGGCGTCACTCCATTCCTTGCAAT	NFAT activating protein with ITAM motif 1
NFATC2	NM_173091.2	nuclear factor of activated T-cells, cytoplasmic, calcineurin-dependent 2,nuclear factor of activated T-cells 2;NF-ATP,NFATp,NFAT1	CGGGTGAGACTGGTTTTCCGAGTTCACATCCCAGAGTCCAGTGGCAGAATCGTCTCTTTACAGACTGCATCTAACCCCATCGAGTGCTCCCAGCGATCTG	nuclear factor of activated T cells 2
NFIL3	NM_001289999.1	IL3BP1;interleukin-3 binding protein 1;E4BP4,NFIL3A,NF-IL3A;E4 promoter-binding protein,adenovirus E4 promoter region binding protein	CAACTGTGGTTAAAGTTCCAGAAGTGAATTCCTCTGCCTTGCCACACAAGCTCCGGATCAAAGCCAAAGCCATGCAGATCAAAGTAGAAGCCTTTGATAA	nuclear factor, interleukin 3 regulated
NFKB1	NM_003998.3	nuclear factor of kappa light polypeptide gene enhancer in B-cells 1;KBF1,p105,NFKB-p50,p50,NF-kappaB,NFkappaB,NF-kB1;Nuclear factor NF-kappa-B p105 subunit	GCAGCTGTATAAGTTACTAGAAATTCCTGATCCAGACAAAAACTGGGCTACTCTGGCGCAGAAATTAGGTCTGGGGATACTTAATAATGCCTTCCGGCTG	nuclear factor kappa B subunit 1
NFKB2	NM_001077494.3	nuclear factor of kappa light polypeptide gene enhancer in B-cells 2 (p49/p100);LYT-10,p52,p105,NF-kB2,p49/p100	GTGCTCCTGAGCTGCTGCGTGCACTGCTTCAGAGTGGAGCTCCTGCTGTGCCCCAGCTGTTGCATATGCCTGACTTTGAGGGACTGTATCCAGTACACCT	nuclear factor kappa B subunit 2
NFKBIA	NM_020529.2	NFKBI;nuclear factor of kappa light polypeptide gene enhancer in B-cells inhibitor, alpha;IKBA,MAD-3,IkappaBalpha;NF-kappa-B inhibitor alpha	ATTTATTGTGCTTCGAGTGACTGACCCCAGTGGTATCCTGTGACATGTAACAGCCAGGAGTGTTAAGCGTTCAGTGATGTGGGGTGAAAAGTTACTACCT	NFKB inhibitor alpha

NFKBIE	NM_004556.2	nuclear factor of kappa light polypeptide gene enhancer in B-cells inhibitor, epsilon;IKBE;NF-kappa-B inhibitor epsilon	GGAGGTCCTGGACATTCAAAATAACCTTTACCAGACAGCACTCCATCTGGCTGTACATCTGGACCAACCGGGCGCAGTTCGGGCACTGGTGCTGAAGGGG	NFKB inhibitor epsilon
NGFR	NM_002507.3	nerve growth factor receptor (TNFR superfamily, member 16);TNFRSF16,CD271,p75NTR;low affinity nerve growth factor receptor,TNFR superfamily, member 16	GCGGGCTCGGGCCTCGTGTTCTCCTGCCAGGACAAGCAGAACACCGTGTGCGAGGAGTGCCCCGACGGCACGTATTCCGACGAGGCCAACCACGTGGACC	nerve growth factor receptor
NID2	NM_007361.3	osteonidogen	GGCCTGTTTGGCTGGCTCTTTGCTTTAGAAAAACCTGGCTCTGAGAACGGCTTCAGCCTCGCAGGTGCTGCCTTTACCCATGACATGGAAGTTACATTCT	nidogen 2
NKG7	NM_005601.3	natural killer cell group 7 sequence;GIG1,GMP-17;granule membrane protein 17	TGTGGCGGTCCCCGTCCTGGCTATGAAACCTTGTGAGCAGAAGGCAAGAGCGGCAAGATGAGTTTTGAGCGTTGTATTCCAAAGGCCTCATCTGGAGCCT	natural killer cell granule protein 7
NLRC5	NM_032206.4	NOD27,CLR16.1,FLJ21709;nucleotide-binding oligomerization domain, leucine rich repeat and CARD domain containing 5,NOD-like receptor C5	TCCTGCTGGCCAACACCTTAAGCCTGTGTCCACGGGTTAAAAAGGTGGATCTCAGGTCCCTGCACCATGCAACTTTGCACTTCAGATCCAACGAGGAGGA	NLR family CARD domain containing 5
NLRP3	NM_001079821.2	C1orf7,CIAS1,DFNA34;cold autoinflammatory syndrome 1,deafness, autosomal dominant 34;AGTAVPRL,AII,AVP,FCAS,FCU,NALP3,PYPAF1,MWS,CLR1.1;Cryopyrin,nucleotide-binding oligomerization domain, leucine rich repeat and pyrin domain containing 3	GCCAAGACCACAGCTCTGTGATCCTTCCGGTGGAGTGTCGGAGAAGAGAGCTTGCCGACGATGCCTTCCTGTGCAGAGCTTGGGCATCTCCTTTACGCCA	NLR family pyrin domain containing 3
NOD2	NM_02216	IBD1,CARD15;caspase recruitment domain family, member 15;BLAU,CD,PSORAS1,CLR16.3,NLRC2;nucleotide-binding oligomerization domain, leucine rich repeat and CARD domain containing 2,NOD-like receptor C2,NLR family, CARD domain containing 2	GGGCCAGAATTTCAAACGGCCTCACTAGGCTTCTGGTTGATGCCTGTGAACTGAACTCTGACAACAGACTTCTGAAATAGACCC	nucleotide binding oligomerization domain containing 2

	2.1		ACAAGAGGCAGTTCCA	
NOS2	NM_000625.4	NOS2A;nitric oxide synthase 2A (inducible, hepatocytes),nitric oxide synthase 2, inducible;iNOS,NOS,HEP-NOS	CCCCAGCGGAGTGATGGCAAGCACGACTTCCGGGTGTGGAATGCTCAGCTCATCCGCTATGCTGGCTACCAGATGCCAGATGGCAGCATCAGAGGGGACC	nitric oxide synthase 2
NOTCH1	NM_017617.3	TAN1;Notch (Drosophila) homolog 1 (translocation-associated),Notch homolog 1, translocation-associated (Drosophila),notch 1	CGGCATCAACTCGTTCACCTGCCTGTGTCCACCCGGCTTCACGGGCAGCTACTGCCAGCACGATGTCAATGAGTGCGACTCACAGCCCTGCCTGCATGGC	notch receptor 1
NOTCH2	NM_024408.3	Notch (Drosophila) homolog 2,Notch homolog 2 (Drosophila),notch 2	AAGAGTCACCAAATTTTGAGAGTTATACTTGCTTGTGTGCTCCTGGCTGGCAAGGTCAGCGGTGTACCATTGACATTGACGAGTGTATCTCCAAGCCCTG	notch receptor 2
NRAS	NM_002524.4	neuroblastoma RAS viral (v-ras) oncogene homolog,neuroblastoma RAS viral oncogene homolog;N-ras	CACCAGTAACTCCAGCTAGTAATTTTGCTAGGTAGCTGCAGTTAGCCCTGCAAGGAAAGAAGAGGTCAGTTAGCACAAACCCTTTACCATGACTGGAAAA	NRAS proto-oncogene, GTPase
NT5E	NM_002526.3	NT5;5' nucleotidase (CD73),5'-nucleotidase, ecto (CD73);CD73,eN,eNT,CALJA	CTCTGAGTGGGTGGTCAGAAAATACCCATGCTGATGAAATGACCTATGCCCAAAGAACAAATACTTAACGTGGGAGTGGAACCACATGAGCCTGCTCAGC	5'-nucleotidase ecto
OAS1	NM_016816.3	OIAS;2',5'-oligoadenylate synthetase 1 (40-46 kD),2'-5'-oligoadenylate synthetase 1, 40/46kDa;OIASI,IFI-4;2'-5' oligoadenylate synthase 1	CTCCTGACGGTCTATGCTTGGGAGCGAGGGAGCATGAAAACACATTTCAACACAGCCCAGGGATTTCGGACGGTCTTGGAATTAGTCATAAACTACCAGC	2'-5'-oligoadenylate synthetase 1
OAS2	NM_016	2'-5'-oligoadenylate synthetase 2 (69-71 kD),2'-5'-oligoadenylate synthetase 2, 69/71kDa	TTCAGTGACTTAAAACAATTCCAGGATCAGAAGAGAAGCCAACGTGACATCCTCGATAA	2'-5'-oligoadenylate synthetase 2

	817.2		AACTGGGGATAAGCTGAAGTTCTGTCTGTTCACGAAGTGGT	
OAS3	NM_006187.3	2'-5'-oligoadenylate synthetase 3 (100 kD),2'-5'-oligoadenylate synthetase 3, 100kDa	GGGGACCTTGACAAGTTCATCAGTGAATTTCTCCAGCCCAACCGCCAGTTCCTGGCCCAGGTGAACAAGGCCGTTGATACCATCTGTTCATTTTTGAAGG	2'-5'-oligoadenylate synthetase 3
OASL	NM_003733.2	2'-5'-oligoadenylate synthetase-like;TRIP14,p59OASL,OASL1	CTGTACATTTCTGCCATGTACTCCAGAACTCATCCTGTCAATCACTCTGTCCCATTGTCTACTGGGAAGGTCCCAGGTCTTCACCAGTTTTACAATGAGT	2'-5'-oligoadenylate synthetase like
OLFML2B	NM_015441.1	olfactomedin-like 2B;DKFZP586L151	TTGACCAGTAACCACCTTCCTTCAAGCCTTCAGCCCCTCCAGCTCCAAGTCTCAGATCTCGACCATTGAAAAGGTTTCTTCATCTGGGTCTTGCAGGAGG	olfactomedin like 2B
OLR1	NM_002543.3	oxidised low density lipoprotein (lectin-like) receptor 1;LOX-1,SCARE1,CLEC8A	GGGATTAGTAGTGACCATTATGGTGCTGGGCATGCAATTATCCCAGGTGTCTGACCTCCTAACACAAGAGCAAGCAAACCTAACTCACCAGAAAAAGAAA	oxidized low density lipoprotein receptor 1
OTOA	NM_001161683.1	DFNB22;deafness, autosomal recessive 22;CT108;cancer/testis antigen 108	GTTTCCAGATTGTCTATGCCACCTTTCCTCTTGGCTGCACTCCCGGCCCGCTACCTGGCTTCTGTCCCAGCCTCCCAGTGTGTGCCCTTTCTGATCAGCC	otoancorin
P2RY13	NM_023914.2	GPR94,GPR86;G protein-coupled receptor 86,purinergic receptor P2Y, G-protein coupled, 13;FKSG77,P2Y13	GAAAAAATACATAAAGGAGTAGTTAAGCTCTGTAAATGTGCCACGAGCTCCAACACGACCATCGTAGGGTGAAGCCCACGTTTTCTTCCATGGCCTCAAA	purinergic receptor P2Y13
P4H	NM_0	P4HA;procollagen-proline, 2-oxoglutarate 4-dioxygenase (proline 4-hydroxylase), alpha polypeptide I,prolyl 4-hydroxylase, alpha	CAAGCCCTAAGGCAACTGGATGAAGGCGAGATTTCT	prolyl 4-hydroxylase subunit alpha 1

A1	00917.3	polypeptide I;C-P4Halpha(I);collagen prolyl 4-hydroxylase alpha(I)	ACCATAGATAAAGTCTCTGTTCTAGATTATTTGAGCTATGCGGTATATCAGCAGGGAGACCTGG	
P4HA2	NM_001017973.1	procollagen-proline, 2-oxoglutarate 4-dioxygenase (proline 4-hydroxylase), alpha polypeptide II,prolyl 4-hydroxylase, alpha polypeptide II;C-P4Halpha(II);4-PH alpha 2,collagen prolyl 4-hydroxylase alpha(II)	GGCCCGAGTAAATCGTCGGATGCAGCATATCACAGGGTTAACAGTAAAGACTGCAGAATTGTTACAGGTTGCAAATTATGGAGTGGGAGGACAGTATGAA	prolyl 4-hydroxylase subunit alpha 2
PALMD	NM_017734.4	C1orf11;FLJ20271,PALML	CCCAGCACACCCTCATTACATGTGTCTGTCTGGCCTGATCTGTGCATCTGCTCGGAGACGCTCCTGACAAGTCGGGAATTTCTCTATTTCTCCACTGGTG	palmdelphin
PARP12	NM_022750.3	ZC3HDC1;zinc finger CCCH-type domain containing 1;FLJ22693,PARP-12,ZC3H1,ARTD12	AATCCCAAAATAGAAAGGATCCTGTGCTCTGAGTCAGCCAGTACCTTTCACTCTCATTGTCTGAACTTTAACGCCATGACTTACGGTGCTACCCAGGCTC	poly(ADP-ribose) polymerase family member 12
PARP4	NM_006437.3	ADPRTL1;ADP-ribosyltransferase (NAD+; poly (ADP-ribose) polymerase)-like 1;VAULT3,p193,VPARP,VWA5C,ARTD4;von Willebrand factor A domain containing 5C,vault poly(ADP-ribose) polymerase	AATGTGAGGCCCTTGTTGCATGGTTCTCCTGTACAAAACATCGTGGGAATCTTGTGTCGAGGGTTGCTTTTACCCAAAGTAGTGGAAGATCGTGGTGTGC	poly(ADP-ribose) polymerase family member 4
PARP9	NM_001146103.1	BAL,BAL1,ARTD9	CAGGGCCACATTGAATGGCAGACGGCAGATGTAATTGTTAATTCTGTAAACCCACATGATATTACAGTTGGACCTGTGGCAAAGTCAATTCTACAACAAG	poly(ADP-ribose) polymerase family member 9
PC	NM_00092	PCB	ACGGAGCTGGGCATCCGCACCGTAGCCATCTACTCTGAGCAGGACACGGGCCAGATGCACCGGCAGAAAGCAGATGAAGCCTA	pyruvate carboxylase

	0.3		TCTCATCGGCCGCGGCC	
PCK2	NM_001018073.2	phosphoenolpyruvate carboxykinase 2 (mitochondrial);PEPCK,PEPCK2	AACCCTGAGAACGGCTTCTTTGGGGTTGCCCCTGGTACCTCTGCCACCACCAATCCCAACGCCATGGCTACAATCCAGAGTAACACTATTTTTACCAATG	phosphoenolpyruvate carboxykinase 2, mitochondrial
PDCD1	NM_005018.1	SLEB2;systemic lupus erythematosus susceptibility 2;CD279,PD1,hSLE1,PD-1	CTTCTTCCCAGCCCTGCTCGTGGTGACCGAAGGGGACAACGCCACCTTCACCTGCAGCTTCTCCAACACATCGGAGAGCTTCGTGCTAAACTGGTACCGC	programmed cell death 1
PDCD1LG2	NM_025239.3	PD-L2,Btdc,PDL2,bA574F11.2,CD273,B7-DC;B7 dendritic cell molecule	TGTGGAGCTGTGGCAAGTCCTCATATCAAATACAGAACATGATCTTCCTCCTGCTAATGTTGAGCCTGGAATTGCAGCTTCACCAGATAGCAGCTTTATT	programmed cell death 1 ligand 2
PDGFA	NM_033023.4	platelet-derived growth factor alpha polypeptide;PDGF1,PDGF-A;PDGF A-chain,platelet-derived growth factor alpha chain	CCGTTTGTGGCTGAGTGACAACTTGTTCCCCGCAGTGCACACCTAGAATGCTGTGTTCCCACGCGGCACGTGAGATGCATTGCCGCTTCTGTCTGTGTTG	platelet derived growth factor subunit A
PDGFB	NM_002608.2	SIS;platelet-derived growth factor beta polypeptide (simian sarcoma viral (v-sis) oncogene homolog),platelet-derived growth factor beta polypeptide;SSV;oncogene SIS,becaplermin	AAAAAGAAGGACTGAACTCCATCGCCATCTTCTTCCCTTAACTCCAAGAACTTGGGATAAGAGTGTGAGAGAGACTGATGGGGTCGCTCTTTGGGGGAAA	platelet derived growth factor subunit B
PDGFRB	NM_002609.3	PDGFR;platelet-derived growth factor receptor, beta polypeptide;JTK12,CD140b,PDGFR1	AAGGACACCATGCGGCTTCCGGGTGCGATGCCAGCTCTGGCCCTCAAAGGCGAGCTGCTGTTGCTGTCTCTCCTGTTACTTCTGGAACCACAGATCTCTC	platelet derived growth factor receptor beta
PDK1	NM_002	pyruvate dehydrogenase kinase, isoenzyme 1,pyruvate dehydrogenase kinase, isozyme 1	TTCTCAGGACACCATCCGTTCAATTGGTACAAAGCTGGTATATCCAGAGTCTTCAGGAGCT	pyruvate dehydrogenase kinase 1

	610.3		TCTTGATTTTAAGGACAAAAGTGCTGAGGATGCTAAAGC	
PDZK1IP1	NM_005764.3	DD96,MAP17,SPAP	GCAAGGCCTGGGGAACCTTCAGCCCTGGATGCAGGGCCTTATCGCGGTGGCCGTGTTCCTGGTCCTCGTTGCAATCGCCTTTGCAGTCAACCACTTCTGG	PDZK1 interacting protein 1
PECAM1	NM_000442.4	platelet/endothelial cell adhesion molecule 1;CD31;CD31 antigen	CTGTATTTCAAGACCTCTGTGCACTTATTTATGAACCTGCCCTGCTCCCACAGAACACAGCAATTCCTCAGGCTAAGCTGCCGGTTCTTAAATCCATCCT	platelet and endothelial cell adhesion molecule 1
PF4	NM_002619.3	SCYB4,CXCL4;chemokine (C-X-C motif) ligand 4	AGGACAGCCGGGAATAAAACGTGCCGGCGAGGCTCAGGAGTCATTGGCCACAGAGACCCAGCCCGAGTTTCCCATCGCACTGAGCACTGAGATCCTGCTG	platelet factor 4
PFKFB3	NM_004566.3		TGACTCCTCCAGGAAACACTGAGGCAGACGTGTCGGTTCCATTCCATTTCCATTTCTGCAGCTTAGCTTGTGTCCTGCCCTCCGCCCGAGGCAAAACGTA	6-phosphofructo-2-kinase/fructose-2,6-biphosphatase 3
PFKM	NM_000289.5	PFKX;phosphofructokinase, polypeptide X;PFK-1,PPP1R122;protein phosphatase 1, regulatory subunit 122	GAGCTGGAGGAGCAGGCAGTGGGTGGGAGCTCCTTTTAGGTAGAATTTAACATGACTTCTGCCCCAGCTTTATCTGTCACACAAGGCTGGGCACCTCTAG	phosphofructokinase, muscle
PGPEP1	NM_001300927.1	PGP-I,Pcp,PGP,PGPI;pyroglutamyl aminopeptidase	TTTCTAGGAAAAAACCCTTCCCTGCCAAAGGTGACTGTGTTTTCTGCCGCCGAAGGAGGGCCCGGTCCCTCCAGGCTCAGTGTGGCTTCTCCCTGACCCC	pyroglutamyl-peptidase I
PIA	NM_0	Piasg,PIASY,FLJ12419,ZMIZ6;zinc finger, MIZ-type containing 6	ACCGAATTAGTCCCACAGAACAACGAGAAGCTTCAG	protein inhibitor of activated STAT 4

S4	158997.2		GAGAGCCCGTGCATCTTCGCATTGACGCCAAGACAGGTGGAGTTGATCCGGAACTCCAGGGAAC	
PIK3CA	NM_006218.2	phosphoinositide-3-kinase, catalytic, alpha polypeptide,phosphatidylinositol-4,5-bisphosphate 3-kinase, catalytic subunit alpha;PI3K	ATCATGGTGGCTGGACAACAAAAATGGATTGGATCTTCCACACAATTAAACAGCATGCATTGAACTGAAAAGATAACTGAGAAAATGAAAGCTCACTCTG	phosphatidylinositol-4,5-bisphosphate 3-kinase catalytic subunit alpha
PIK3CD	NM_001350234.1	phosphoinositide-3-kinase, catalytic, delta polypeptide,phosphatidylinositol-4,5-bisphosphate 3-kinase, catalytic subunit delta;p110D;phosphatidylinositol 3-kinase, catalytic, delta polypeptide,phosphoinositide-3-kinase C	CAGCCTCCTCATCGGCAAAGGCCTCCACGAGTTTGACTCCTTGTGCGACCCAGAAGTGAACGACTTTCGCGCCAAGATGTGCCAATTCTGCGAGGAGGCG	phosphatidylinositol-4,5-bisphosphate 3-kinase catalytic subunit delta
PIK3CG	NM_001282426.1	phosphoinositide-3-kinase, catalytic, gamma polypeptide,phosphatidylinositol-4,5-bisphosphate 3-kinase, catalytic subunit gamma	AACCAGCTTCGCAAGCAATTGGAGGCGATCATAGCCACTGATCCACTTAACCCTCTCACAGCAGAGGACAAAGAATTGCTCTGGCATTTTAGATACGAAA	phosphatidylinositol-4,5-bisphosphate 3-kinase catalytic subunit gamma
PIK3R1	NM_001242466.1	phosphoinositide-3-kinase, regulatory subunit 1 (alpha);GRB1,p85-ALPHA,p85;phosphoinositide-3-kinase regulatory subunit alpha	TGGTTGACTCAAAAAGGTGTTCGGCAAAAGAAGTTGAACGAGTGGTTGGGCAATGAAAACACTGAAGACCAATATTCACTGGTGGAAGATGATGAAGATT	phosphoinositide-3-kinase regulatory subunit 1
PIK3R2	NM_005027.3	phosphoinositide-3-kinase, regulatory subunit 2 (beta);P85B,p85;phosphoinositide-3-kinase regulatory subunit beta	TGGCCCTTGTAAGGGTCATGGAATAATTTGAAGCGAGGCATGAGCGGCCCCTGTGGTCGCCTGTGACTGCTGGAGATAGAGGTCCCAGCACCCCAAGCCA	phosphoinositide-3-kinase regulatory subunit 2
PIK3	NM_01	phosphoinositide-3-kinase, regulatory subunit 5;P101-PI3K,p101	AATTTCTCTCAAGTTTCCTGAGTCTCCAGAAAAACAGCACTAACGCTGGA	phosphoinositide-3-kinase regulatory subunit 5

R5	4308.3		CCTGTCTACTCTCAGAACCCGGCACAGATTCTCTCTTGATCTCCTTTTGG	
PKM	NM_001206796.2	PKM2;pyruvate kinase, muscle;THBP1,OIP3,PK3	CATCCAGGATCTGAAGTTTGGGGTCGAGCAGGATGTTGATATGGTGTTTGCGTCATTCATCCGCAAGGCATCTGATGTCCATGAAGTTAGGAAGGTCCTG	pyruvate kinase M1/2
PLA1A	NM_015900.2	ps-PLA1	CAACCGAGTTTGGAAAAAAGACCGGACTACCATTATTGGGAAGTTCTGCACTGCCCTTTTGCCTGTCAATGACAGAGAAAAGATGGTCTGCTTACCTGAA	phospholipase A1 member A
PLA2G2A	NM_000300.2	PLA2B,PLA2L;phospholipase A2, group IIA (platelets, synovial fluid)	TTCCCTGGAAACCTTCCACCCAGTGCTGAATTTCCCTCTCTCATACCCTCCCTCCCTACCCTAACCAAGTTCCTTGGCCATGCAGAAAGCATCCCTCACC	phospholipase A2 group IIA
PLOD2	NM_182943.2	procollagen-lysine, 2-oxoglutarate 5-dioxygenase (lysine hydroxylase) 2,procollagen-lysine, 2-oxoglutarate 5-dioxygenase 2;LH2;lysyl hydroxlase 2,procollagen-lysine 5-dioxygenase	ATAAACATTGCACTTAATAACGTGGGAGAAGACTTTCAGGGAGGTGGTTGCAAATTTCTAAGGTACAATTGCTCTATTGAGTCACCACGAAAAGGCTGGA	procollagen-lysine,2-oxoglutarate 5-dioxygenase 2
PMS2	NM_000535.6	PMSL2;postmeiotic segregation increased (S. cerevisiae) 2,PMS2 postmeiotic segregation increased 2 (S. cerevisiae),PMS1 homolog 2, mismatch repair protein;H_DJ0042M02.9,HNPCC4,MLH4	TCAGGTTTCATTTCACAATGCACGCATGGAGTTGGAAGGAGTTCAACAGACAGACAGTTTTTCTTTATCAACCGGCGGCCTTGTGACCCAGCAAAGGTCT	PMS1 homolog 2, mismatch repair system component
PNOC	NM_001284244	PPNOC,N/OFQ,NOP;nocistatin,orphanin FQ	CCCCAGAGCATGTGGCGGCTGCTCTCTACCAGCCGAGAGCTTCGGAGATGCAGCATCTGCGGCGAATGCCCCGAGTCCGGAGCTTGTTCCAGGAGCAGGA	prepronociceptin

	4.1			
POLD1	NM_001256849.1	POLD;polymerase (DNA directed), delta 1, catalytic subunit (125kD),polymerase (DNA) delta 1, catalytic subunit;CDC2;CDC2 homolog (S. cerevisiae)	TCCAACATCCGGGACTCTTCATTCCAGTCCAAGCAGACGGGCCGGCGGGACACCAAGGTTGTCAGCATGGTGGGCCGCGTGCAGATGGACATGCTGCAGG	DNA polymerase delta 1, catalytic subunit
PPARG	NM_005037.5	peroxisome proliferative activated receptor, gamma,peroxisome proliferator-activated receptor gamma;PPARG1,PPARG2,NR1C3,PPARgamma	TATTCTGAGAAGACTCAGCTCTACAATAAGCCTCATGAAGAGCCTTCCAACTCCCTCATGGCAATTGAATGTCGTGTCTGTGGAGATAAAGCTTCTGGAT	peroxisome proliferator activated receptor gamma
PPARGC1B	NM_001172699.1	peroxisome proliferative activated receptor, gamma, coactivator 1, beta,peroxisome proliferator-activated receptor gamma, coactivator 1 beta;PERC,PGC1B;PPARgamma coactivator 1 beta	TCCTGGGCTGAGTTCTCCATTCTGAGGGAACTTCTGGCTCAAGACGTGCTCTGTGATGTCAGCAAACCCTACCGTCTGGCCACGCCTGTTTATGCCTCCC	PPARG coactivator 1 beta
PRF1	NM_001083116.1	perforin 1 (pore forming protein);PFP,P1,HPLH2;Perforin,perforin 1 (preforming protein)	TGCAGCCCAGAAGACCCACCAGGACCAGTACAGCTTCAGCACTGACACGGTGGAGTGCCGCTTCTACAGTTTCCATGTGGTACACACTCCCCCGCTGCAC	perforin 1
PRKAA2	NM_006252.3	PRKAA;protein kinase, AMP-activated, alpha 2 catalytic subunit;AMPK,AMPKa2	GAAGATTTCAGGAGATGCAGTCCAGCACAATTAGAGCTGGAACATTGTTACAGCAGGCTTTTTGTTGCTCATGGGCAGATAGAGGGAAAGAATCAGTTGT	protein kinase AMP-activated catalytic subunit alpha 2
PRKACB	NM_182894	protein kinase, cAMP-dependent, catalytic, beta,protein kinase, cAMP-dependent, beta catalytic subunit;PKACb	GAGTTAAAGGCAGAACTTGGACATTATGTGGAACTCCAGAGTATTTGGCTCCAGAAATAATTCTCAGCAAGGGCTACAATAAGG	protein kinase cAMP-activated catalytic subunit beta

	8.2		CAGTGGATTGGTGGGC	
PRKCA	NM_002737.2	PKCA;protein kinase C, alpha;PKCÎ±	CTTTCCTTTGGAGTTTCGGAGCTGATGAAGATGCCGGCCAGTGGATGGTACAAGTTGCTTAACCAAGAAGAAGGTGAGTACTACAACGTACCCATTCCGG	protein kinase C alpha
PRKX	NM_005044.4	PKX1	CCCGCGCTCTGCCCCAGCCCTGAGGCGCTGTCGCCGGAGCCGCCTGTGTACAGCCTGCAGGACTTTGACACGCTGGCCACCGTGGGCACTGGGACGTTCG	protein kinase X-linked
PRLR	NM_000949.5		ACCCTACCTGTGGATTAAATGGTCTCCACCTACCCTGATTGACTTAAAAACTGGTTGGTTCACGCTCCTGTATGAAATTCGATTAAAACCCGAGAAAGCA	prolactin receptor
PROM1	NM_001145847.1	PROML1,MCDR2,STGD4;prominin (mouse)-like 1,macular dystrophy, retinal 2,Stargardt disease 4 (autosomal dominant);AC133,CD133,RP41,CORD12	AGATGCTCCTAAGGCTTGGAATTATGAATTGCCTGCAACAAATTATGAGACCCAAGACTCCCATAAAGCTGGACCCATTGGCATTCTCTTTGAACTAGTG	prominin 1
PRR5	NM_015366.3	proline rich 5 (renal);PP610,FLJ20185k,Protor-1;protein observed with Rictor-1	GTCCATGTGGCGTGTGTGTGAGTGAGACTTTTTTACTGCGTCCCGTCCCGCCAGCCCTATCGGCCTCGTCACTGGCCTTGGTCACTTTGTATTTCTGTCT	proline rich 5
PSMB10	NM_002801.3	MECL1;proteasome (prosome, macropain) subunit, beta type, 10,proteasome subunit beta 10;LMP10,MGC1665,beta2i;proteasome subunit Î²2i	CCATCGCGGGCCTGGTGTTCCAAGACGGGGTCATTCTGGGCGCCGATACGCGAGCCACTAACGATTCGGTCGTGGCGGACAAGAGCTGCGAGAAGATCCA	proteasome 20S subunit beta 10
PSMB5	NM_01	proteasome (prosome, macropain) subunit, beta type, 5,proteasome subunit beta 5;MB1;proteasome subunit X	CGTGGACAGTGAAGGGAACCGGATTTCAGGGGCCACCTTCTCTGTAGGTTCTGGCTCTG	proteasome 20S subunit beta 5

	1307 25.1		TGTATGCATATGGGGTCATGGATCGGGGCTATTCCTATGAC	
PSMB8	NM_004159.4	LMP7;proteasome (prosome, macropain) subunit, beta type, 8 (large multifunctional protease 7),large multifunctional peptidase 7,proteasome (prosome, macropain) subunit, beta type, 8,proteasome subunit beta 8;RING10,D6S216E,PSMB5i,beta5i;proteasome subunit Î²5i	ACTCGGCTCTCAGGAAATATGTTCTCCACGGGTAGTGGGAACACTTATGCCTACGGGGTCATGGACAGTGGCTATCGGCCTAATCTTAGCCCTGAAGAGG	proteasome 20S subunit beta 8
PSMB9	NM_002800.4	LMP2;proteasome (prosome, macropain) subunit, beta type, 9 (large multifunctional protease 2),large multifunctional peptidase 2,proteasome (prosome, macropain) subunit, beta type, 9,proteasome subunit beta 9;RING12,beta1i,PSMB6i;proteasome subunit Î²1i	AATATCGAGAGGACTTGTCTGCACATCTCATGGTAGCTGGCTGGGACCAACGTGAAGGAGGTCAGGTATATGGAACCCTGGGAGGAATGCTGACTCGACA	proteasome 20S subunit beta 9
PTCD2	NM_001284403.1	FLJ12598	TGAATAGCCCTGAGTCTTTCAAAATCTGTACTACATTAAGAGAAGAAGCTCTACTCAAAGGAGAAATTCTCTCCAGGAGAGCATCCTGTTTCGCTGTGGC	pentatricopeptide repeat domain 2
PTEN	NM_000314.6	BZS,MHAM;MMAC1,TEP1,PTEN1;mutated in multiple advanced cancers 1	GGAAACAAGAATATTCAGTCCAAACTGTTGTAAGGACAGTACCTGAAAACCAGGAAACAGGATAATGGAAAAAGTCTTTTAAAGATGAAATGTTGGAGCC	phosphatase and tensin homolog
PTGER4	NM_000958.2	prostaglandin E receptor 4 (subtype EP4);EP4	CCACTACGTGGACAAGCGATTGGCGGGCCTCACGCTCTTTGCAGTCTATGCGTCCAACGTGCTCTTTTGCGCGCTGCCCAACATGGGTCTCGGTAGCTCG	prostaglandin E receptor 4
PTGS2	NM_000963.1	prostaglandin-endoperoxide synthase 2 (prostaglandin G/H synthase and cyclooxygenase);COX2;prostaglandin G/H synthase 2,cyclooxygenase 2	GCTACAAAAGCTGGGAAGCCTTCTCTAACCTCTCCTATTATACTAGAGCCCTTCCTCCTGTGCCTGATGATTGCCCGACTCCCTTGGGTGTCAAAGGTAA	prostaglandin-endoperoxide synthase 2

PTPN11	NM_001330437.1	NS1;Noonan syndrome 1,protein tyrosine phosphatase, non-receptor type 11;BPTP3,SH-PTP2,SHP-2,PTP2C,SHP2	ATGGTTTCACCCAAATATCACTGGTGTGGAGGCAGAAAACCTACTGTTGACAAGAGGAGTTGATGGCAGTTTTTTGGCAAGGCCTAGTAAAAGTAACCCT	protein tyrosine phosphatase non-receptor type 11
PTPRC	NM_080921.3	CD45;LCA,T200,GP180	CTAGCAAGTGGTTTGTTCTTAGGGTAACAGAGGAGGAAATTGTTCCTCGTCTGATAAGACAACAGTGGAGAAAGGACGCATGCTGTTTCTTAGGGACACG	protein tyrosine phosphatase receptor type C
PVR	NM_001135768.2	PVS;poliovirus receptor;CD155,HVED,Necl-5,NECL5,Tage4;nectin-like 5	CCTACAGGTGCCCAACATGGAGGTGACGCATGTGTCACAGCTGACTTGGGCGCGGCATGGTGAATCTGGCAGCATGGCCGTCTTCCACCAAACGCAGGGC	PVR cell adhesion molecule
PVRIG	NM_024070.3	poliovirus receptor related immunoglobulin domain containing;MGC2463,C7orf15,CD112R;CD112 receptor,nectin-2 receptor	TACTTTAATTCTTGGGCCTCCAATAAGTGTCCCATAGGTGTCTGGCCAGGCCCACCTGCTGCGGATGTGGTCTCTGTGTGTGCGTGTGTGGGCACAGGTG	PVR related immunoglobulin domain containing
RAD50	NM_005732.3	RAD50 (S. cerevisiae) homolog,RAD50 homolog (S. cerevisiae),RAD50 homolog, double strand break repair protein;hRad50,RAD50-2	AAGGAAATTGGTAGACTGTCATCGTGAACTGGAAAAACTAAATAAAGAATCTAGGCTTCTCAATCAGGAAAAATCAGAACTGCTTGTTGAACAGGGTCGT	RAD50 double strand break repair protein
RAD51	NM_133487.3	RAD51A,RECA;RAD51 (S. cerevisiae) homolog (E coli RecA homolog),RAD51 homolog (RecA homolog, E. coli) (S. cerevisiae),RAD51 homolog (S. cerevisiae);HsRad51,HsT16930,BRCC5,FANCR;BRCA1/BRCA2-containing complex, subunit 5	GGGGTATGAAGTATCTTTGACATGGTGCCTTAGGAATGACTTGGGTTTAACAAGCTGTCTACTGGACAATCTTATGTTTCCAAGAGAACTAAAGCTGGAG	RAD51 recombinase
RAD5	NM_05	RAD51 (S. cerevisiae) homolog C,RAD51 homolog C (S. cerevisiae);RAD51L2,FANCO	GCCCCAGCGAGGGCGTGCGGAGTTTGGCTGCTCCGGGGTTAGCAGGTG	RAD51 paralog C

1C	8216.2		AGCCTGCGATGCGCGGGAAGACGTTCCGCTTTGAAATGCAGCGGGATTTGGT	
RASAL1	NM_001193520.1	RAS protein activator like 1 (GAP1 like);RASAL	GATGTTGTTGCCCCAGATGGGCACTAAATGGCCTCACTCCTTCCTGTTTTCATGTCTGCTAATCCCTATAACCTCACTGATTCTTCTGTACCCTGCCCTT	RAS protein activator like 1
RASGRF1	NM_001145648.1	GRF1;CDC25L,CDC25,GRF55,H-GRF55,GNRP,PP13187	AGAGGAGTCAGATATTGATCAAAACCAGAGTGATGATGGTGATACTGAAACATCACCAACTAAATCTCCAACAACACCCAAATCAGTCAAAAACAAAAAT	Ras protein specific guanine nucleotide releasing factor 1
RB1	NM_000321.2	OSRC;osteosarcoma,retinoblastoma 1;RB,PPP1R130;prepro-retinoblastoma-associated protein,protein phosphatase 1, regulatory subunit 130	TCCTAAACCACTGAAAAAACTACGCTTTGATATTGAAGGATCAGATGAAGCAGATGGAAGTAAACATCTCCCAGGAGAGTCCAAATTTCAGCAGAAACTG	RB transcriptional corepressor 1
RBL2	NM_001323611.1	retinoblastoma-like 2;Rb2,p130	CACACCACTAACTGGTGTTAGGTACATTAAGGAGAATAGCCCTTGTGTGACTCCAGTTTCTACAGCTACGCATAGCTTGAGTCGTCTTCACACCATGCTG	RB transcriptional corepressor like 2
RELA	NM_021975.3	NFKB3;nuclear factor of kappa light polypeptide gene enhancer in B-cells 3,v-rel avian reticuloendotheliosis viral oncogene homolog A;p65	GAAGCATTAACTTCTCTGGAAAGGGGGGAGCTGGGGAAACTCAAACTTTTCCCCTGTCCTGATGGTCAGCTCCCTTCTCTGTAGGGAACTCTGGGGTCCC	RELA proto-oncogene, NF-kB subunit
RELB	NM_006	v-rel avian reticuloendotheliosis viral oncogene homolog B (nuclear factor of kappa light polypeptide gene enhancer in B-cells 3);REL-B	GACCAGCAGGGACAGATGCGCCGGATGGATCCTGTGCTTTCCGAGCCCGTCTATGACAA	RELB proto-oncogene, NF-kB subunit

	5 0 9. 3		GAAATCCACAAA CACATCAGAGCT GCGGATTTGCCG AATTA	
R E L N	N M _0 0 5 0 4 5. 3	RL,PRO1598	TATTCAGACCCC AGCATCATCGTG TTATATGCCAAG AATAACTCTGCG GACTGGATTCAG CTAGAGAAAATT AGAGCCCCTTCC AATGTCAGCACA ATCA	reelin
R E N	N M _0 0 0 5 3 7. 3		ACTGGTTCGTCC AATGTTTGGGTG CCCTCCTCCAAGT GCAGCCGTCTCT ACACTGCCTGTG TGTATCACAAGC TCTTCGATGCTTC GGATTCCTCCAG CT	renin
R I C T O R	N M _0 0 1 2 8 5 4 3 9. 1	RPTOR independent companion of MTOR, complex 2;MGC39830,AVO3,PIA,KIAA1999;rapamycin-insensitive companion of mTOR,pianissimo	TCTGACCCGAGA ACCTTCTGATAA CTTAAGAGAGAT TCTCCAAAATGT GGCCAGATTGCA GGGAGTATCAAA TATGAGAAAGCT AGGCCATCTGAA TAAC	RPTOR independent companion of MTOR complex 2
R I P K 1	N M _0 0 3 8 0 4. 3	receptor (TNFRSF)-interacting serine-threonine kinase 1;RIP;receptor-interacting protein kinase 1	AGGCACTGCTTA TTCCAGTGCAGC CAGTCATGGTAA TGCAGTGCACCA GCCCTCAGGGCT CACCAGCCAACC TCAAGTACTGTA TCAGAACAATGG ATTA	receptor interacting serine/threonine kinase 1
R I P K 2	N M _0 0 3 8 2 1. 5	receptor-interacting serine-threonine kinase 2;RICK,RIP2,CARDIAK,CARD3	ATCTCTGAACAT ACCTGTAAATCA TGGTCCACAAGA GGAATCATGTGG ATCCTCTCAGCTC CATGAAAATAGT GGTTCTCCTGAA ACTTCAAGGTCC CTG	receptor interacting serine/threonine kinase 2
R I P K 3	N M _0 0 6 8 7 1. 3	receptor-interacting serine-threonine kinase 3;RIP3	AGATGGTGGAG AACAATATGAAT GCTGCTGTCTCC ACGGTAAAGGAT TTCCTGTCTCAGC TCAGGAGCAGCA ATAGGAGATTTT CTATCCCAGAGT CAGG	receptor interacting serine/threonine kinase 3
R N	N M _0	C10orf59;chromosome 10 open reading frame 59;FLJ11218,renalase	CTCTTTTATGAA GCTGGTACGAAG ATTGATGTCCCTT	renalase, FAD dependent amine oxidase

LS	01031709.2		GGGCTGGGCAGTACATCACCAGTAATCCCTGCATACGCTTCGTCTCCATTGATAATAAGAAGC	
ROBO4	NM_019055.5	roundabout homolog 4 (Drosophila),roundabout, axon guidance receptor, homolog 4 (Drosophila);FLJ20798,MRB,ECSM4;magic roundabout	CCTGACTCTCAGATCTCTTCCCAGAGAAGTCAGCTCCACTGTCGTATGCCCAAGGCTGGTGCTTCTCCTGTAGATTACTCCTGAACCGTGTCCCTGAGAC	roundabout guidance receptor 4
ROCK1	NM_005406.2	p160ROCK	TGAAATATTAAGAAGAGAGAATGAAGAGCTAACAGAGAAAATGAAGAAGGCAGAGGAAGAATATAAACTGGAGAAGGAGGAGGAGATCAGTAATCTTAAG	Rho associated coiled-coil containing protein kinase 1
ROR2	NM_001318204.1	NTRKR2,BDB,BDB1;receptor tyrosine kinase-like orphan receptor 2	GCCCAAATCATAACTTTCAGGATGATTACCACGAGGATGGGTTCTGCCAGCCTTACCGGGGAATTGCCTGTGCACGCTTCATTGGCAACCGGACCATTTA	receptor tyrosine kinase like orphan receptor 2
RORC	NM_001001523.1	RAR-related orphan receptor C;RZRG,RORG,NR1F3,TOR	AGCTGTGATCTTGCCCAGAACCTCTCTTGGCTTCATAAACAGCTGTGAACCCTCCCCTGAGGGATTAACAGCAATGATGGGCAGTCGTGGAGTTGGGGGG	RAR related orphan receptor C
RPL23	NM_000978.3	rpL17,L23	GAGGACGTGGTGGGTCCTCTGGTGCGAAATTCCGGATTTCCTTGGGTCTTCCGGTAGGAGCTGTAATCAATTGTGCTGACAACACAGGAGCCAAAAACCT	ribosomal protein L23
RPL	NM_0	SURF3,TRUP,L7A;surfeit 3,PLA-X polypeptide,surfeit locus protein 3,60S ribosomal protein L7a,;,thyroid hormone receptor uncoupling protein	GAAGACAAAGGCGCTTTGGCTAAGCTGGTGGAAGCTATCAGGACCA	ribosomal protein L7a

7A	0972.2		ATTACAATGACAGATACGATGAGATCCGCCGTCACTGGGGTGGCAATGTCCTGG	
RPS6KB1	NM_001272042.1	STK14A;ribosomal protein S6 kinase, 70kD, polypeptide 1;S6K1,p70(S6K)-alpha,PS6K,S6K	CTATGTGCTAAGCTTAACTGGAAGCCTTGGAATGGGCATAAGTTGTATGTCCTACATTTCATCATTGTCCCGGGCCTGCATTGCACTGGAAAAAAAAATC	ribosomal protein S6 kinase B1
RPTOR	NM_001163034.1	regulatory associated protein of MTOR, complex 1;KOG1,Mip1,KIAA1303,raptor;regulatory associated protein of mTOR	ACTGCGGACGACGCGGACGATGCTGCTGGACACAAAAGTTTCATCTCCGCCACGGTGCAGACGGGGTTCTGCGACTGGAGCGCCCGCTATTTTGCCCAGC	regulatory associated protein of MTOR complex 1
RRM2	NM_001034.1	C2orf48;ribonucleotide reductase M2 polypeptide,chromosome 2 open reading frame 48;FLJ25102	TTCCTTTTGGACCGCCGAGGAGGTTGACCTCTCCAAGGACATTCAGCACTGGGAATCCCTGAAACCCGAGGAGAGATATTTTATATCCCATGTTCTGGCT	ribonucleotide reductase regulatory subunit M2
RSAD2	NM_080657.4	cig5,vig1,Viperin;virus-inhibitory protein, endoplasmic reticulumâ€“associated, interferon-inducible	ATGCGCTTTCTGAACTGTAGAAAGGGACGGAAGGACCCTTCCAAGTCCATCCTGGATGTTGGTGTAGAAGAAGCTATAAAATTCAGTGGATTTGATGAAA	radical S-adenosyl methionine domain containing 2
RUNX3	NM_001031680.2	CBFA3;runt-related transcription factor 3,runt related transcription factor 3;AML2,PEBP2A3	TTGGGTTCCCAGTGCCCAGCACTTTGTAGTCTCATCCCAGATTACTAACCCTTCCTGATCCTGGAGAGGCAGGGATAGTAAATAAATTGCTCTTCCTACC	RUNX family transcription factor 3
S100A	NM_005	S100 calcium-binding protein A12 (calgranulin C);p6,MRP6,CGRP,CAAF1,CAGC,ENRAGE;extracellular newly identified RAGE-binding protein,calcium-binding protein in amniotic fluid 1,migration	CAAGATGAACAGGTCGACTTTCAAGAATTCATATCCCTGGTAGCCATTGCGCTGAAGGCT	S100 calcium binding protein A12

12	621.1	inhibitory factor-related protein 6,neutrophil S100 protein,calgranulin C	GCCCATTACCACACCCACAAAGAGTAGGTAGCTCTCTGAA	
S100A8	NM_002964.4	CAGA,CFAG;calgranulin A,S100 calcium-binding protein A8 (calgranulin A);P8,MRP8,60B8AG,CGLA	GTTAACTTCCAGGAGTTCCTCATTCTGGTGATAAAGATGGGCGTGGCAGCCCACAAAAAAAGCCATGAAGAAAGCCACAAAGAGTAGCTGAGTTACTGGG	S100 calcium binding protein A8
S100A9	NM_002965.3	CAGB,CFAG;calgranulin B,S100 calcium-binding protein A9 (calgranulin B);P14,MIF,NIF,LIAG,MRP14,MAC387,60B8AG,CGLB	TGAACCAGGGGGAATTCAAAGAGCTGGTGCGAAAAGATCTGCAAAATTTTCTCAAGAAGGAGAATAAGAATGAAAAGGTCATAGAACACATCATGGAGGA	S100 calcium binding protein A9
SAMD9	NM_017654.3	C7orf5;chromosome 7 open reading frame 5;KIAA2004,FLJ20073	TGCGGAAAACAGCCATTGAAGATTCGATTCAGACATCTAAGATGGGAAAGCCCAGTAAAAATGCTCCTAAAGACCAAACTGTGTCTCAAAAGGAACGTAG	sterile alpha motif domain containing 9
SAMSN1	NM_001256370.1	NASH1,SASH2,SH3D6B,HACS1,SLy2;nuclear localization signals, SAM and SH3 domain containing 1,SAM and SH3 domain containing 2,hematopoietic adapter-containing SH3 and sterile Î±-motif (SAM) domains 1,Src homology domain 3 (SH3)-containing adapter protein SH3 lymphocyte protein 2	ACAGCAAAAAATCCAAGACTCTGCAGGAGTTCCTAGAGAGGATTCATCTGCAGGAATACACCTCAACACTTTTGCTCAATGGTTATGAGACTCTAGAAGA	SAM domain, SH3 domain and nuclear localization signals 1
SBNO2	NM_001100122.1	KIAA0963;KIAA0963,strawberry notch homolog 2 (Drosophila);FLJ00173,Stno,Sno	ACTGCTTCGTCTCGGCCGCTGAAGGCGTGTTCCTGTCGCTAATTCAGAAGCACTTTCCGTCCACCAAGAGAAAGCGGGACAGAGGAGCGGGCAGCAAGCG	strawberry notch homolog 2
SELE	NM_000450.2	ELAM1,ELAM;endothelial adhesion molecule 1;ESEL,CD62E	ATGAGGCCAGTGCTTATTGTCAGCAAAGGTACACACACCTGGTTGCAATTCAAAACAAAGAAGAGATTGAGTACCTAAACTCCATATTGAGCTATTCACC	selectin E

SELL	NM_000655.4	LYAM1,LNHR;lymphocyte adhesion molecule 1;LSEL,LAM1,LAM-1,hLHRc,Leu-8,Lyam-1,PLNHR,CD62L	GCACTGTAGCCTCGCCGTCTGTGAATTGGACCATCCTATTTAACTGGCTTCAGCCTCCCCACCTTCTTCAGCCACCTCTCTTTTTCAGTTGGCTGACTTC	selectin L
SELP	NM_003005.3	GRMP;selectin P (granule membrane protein 140kD, antigen CD62),selectin P (granule membrane protein 140kDa, antigen CD62);CD62,PSEL,PADGEM,GMP140,CD62P;antigen CD62,granule membrane protein 140kDa	GAATTTCCCAACTCCTTTGCTTCAGTGCCCTGATCTCTGAACTAACAAACCAGAAAGAAGTGGCAGCATGGACTTATCATTACAGCACAAAAGCATACTC	selectin P
SERPINA1	NM_001127702.1	PI;serine (or cysteine) proteinase inhibitor, clade A (alpha-1 antiproteinase, antitrypsin), member 1,serpin peptidase inhibitor, clade A (alpha-1 antiproteinase, antitrypsin), member 1;AAT,A1A,PI1,alpha-1-antitrypsin,A1AT,alpha1AT;protease inhibitor 1 (anti-elastase), alpha-1-antitrypsin	GATTTGGTCAAGGAGCTTGACAGAGACACAGTTTTTGCTCTGGTGAATTACATCTTCTTTAAAGGCAAATGGGAGAGACCCTTTGAAGTCAAGGACACCG	serpin family A member 1
SERPINB5	NM_002639.4	PI5;serine (or cysteine) proteinase inhibitor, clade B (ovalbumin), member 5,serpin peptidase inhibitor, clade B (ovalbumin), member 5;maspin;protease inhibitor 5 (maspin)	GCCTGTTCCTTTTCCACGCATTTTCCAGGATAACTGTGACTCCAGGCCCGCAATGGATGCCCTGCAACTAGCAAATTCGGCTTTTGCCGTTGATCTGTTC	serpin family B member 5
SERPINH1	NM_001207014.1	CBP1,CBP2,SERPINH2;serine (or cysteine) proteinase inhibitor, clade H (heat shock protein 47), member 2,serine (or cysteine) proteinase inhibitor, clade H (heat shock protein 47), member 1, (collagen binding protein 1),serpin peptidase inhibitor, clade H (heat shock protein 47), member 1, (collagen binding protein 1);HSP47,colligen;collagen binding protein 1	CATTCTGCCTGCCCTGAAAGTCCCAGATCAAGCCTGCCTCAATCAGTATTCATATTTATAGCCAGGTACCTTCTCACCTGTGAGACCAAATTGAGCTAGG	serpin family H member 1
SFRP1	NM_003012.3	SARP2,FRP,FRP-1	GTGGGTCACACACACGCACTGCGCCTGTCAGTAGTGGACATTGTAATCCAGTCGGCTTGTTCTTGCAGCATTCCCGCTCCCTTCCCTCCATAGCCACGCT	secreted frizzled related protein 1
SFR	NM_0	secreted frizzled-related protein 4;frpHE,FRP-4,FRPHE,FRZB-2	AATGGAGAGATCAGCTTAGTAAAAGATCCATACAGTGGGAAGAGAGG	secreted frizzled related protein 4

P4	3014.2		CTGCAGGAACAGCGGAGAACAGTTCAGGACAAGAAGAAAACAGCCGGGCGCAC	
SFXN1	NM_001322977.1	FLJ12876,SLC56A1	CTACCACCAAACATTAACATCAAGGAACCTCGATGGGATCAAAGCACTTTCATTGGACGAGCCAATCATTTCTTCACTGTAACTGACCCCAGGAACATTC	sideroflexin 1
SGK1	NM_005627.3	SGK;serum/glucocorticoid regulated kinase	ACGAGCGTTAGAGTGCCGCCTTAGACGGAGGCAGGAGTTTCGTTAGAAAGCGGACGCTGTTCTAAAAAAGGTCTCCTGCAGATCTGTCTGGGCTGTGATG	serum/glucocorticoid regulated kinase 1
SH2D1A	NM_001114937.2	IMD5,LYP;lymphoproliferative syndrome,SH2 domain protein 1A;XLP,MTCP1,DSHP,XLPD,EBVS,SAP;Duncan's disease	CTGCTTGCCACTGGGCTGGATGGCAGCTATTTGCTGAGGGACAGCGAGAGCGTGCCAGGCGTGTACTGCCTATGTGTGCTGTATCACGGTTACATTTATA	SH2 domain containing 1A
SHC2	NM_012435.2	SHC (Src homology 2 domain containing) transforming protein 2;SLI,SCK,SHCB;neuronal Shc adaptor homolog	ACTGATGGCCTCAGCCTCTCCGTGCCTGCCACGCGCCAGGTCATCGCCAACCACCACATGCCGTCCATCTCCTTCGCGTCAGGCGGAGACACGGACATGA	SHC adaptor protein 2
SIGLEC1	NM_023068.3	SN;sialoadhesin,sialic acid binding Ig-like lectin 1, sialoadhesin;SIGLEC-1,CD169,FLJ00051,FLJ00055,FLJ00073,FLJ32150,dJ1009E24.1,sialoadhesin	TGCCTCTGCTCCAGTCATGCTCCGTGTGCTCTACCCTCCCAAGACGCCCACCATGATGGTCTTCGTGGAGCCTGAGGGTGGCCTCCGGGGCATCCTGGAT	sialic acid binding Ig like lectin 1
SIGLEC5	NM_003830	CD33L2;sialic acid binding Ig-like lectin 5;OB-BP2,SIGLEC-5,CD170	ACATGGAAATCAACCAACATGGGTCCTGGAACAGGGCGTTGTGCTCAGTGCTTTCTGGTCTCTCTTCCTTGAATAGAAAGGTCCTG	sialic acid binding Ig like lectin 5

	0.3		CTGGCAAGTTCTCT	
SIGLEC8	NM_014442.2	sialic acid binding Ig-like lectin 8;SIGLEC-8,SAF2,SIGLEC8L,MGC59785	GGCTACCAGGGACCCATCCCTGCCTCTAGCTTCTACTACCCACCATTCTCCTCTCGACCTCTCTGAGGTTGACTATTTTAGATTCCACATAGAGATGAGG	sialic acid binding Ig like lectin 8
SIRPA	NM_001040023.1	PTPNS1;protein tyrosine phosphatase, non-receptor type substrate 1,signal-regulatory protein alpha;SHPS1,SIRP,MYD-1,BIT,P84,SHPS-1,SIRPalpha,CD172a,SIRPalpha2,MFR,SIRP-ALPHA-1	CCCAGGGCAAGCAGATGTCGCAAGCCCTATTTATTCAGTCTTCACTATAACTCTTAGAGTTGAGACGCTAATGTTCATGACTCCTGGCCTTGGGATGCCC	signal regulatory protein alpha
SIRPB2	NM_001134836.1	PTPN1L,PTPNS1L3;protein tyrosine phosphatase, non-receptor type 1-like,protein tyrosine phosphatase, non-receptor type substrate 1-like 3,signal-regulatory protein beta 2;dJ776F14.2	AATGACTTCAGCATTCTTCTGCAAAACGTCTCCAGTGAGGATGCAGGCACCTATTACTGTGTAAAGTTTCAGAGGAAACCCAACAGGCAATACCTGTCTG	signal regulatory protein beta 2
SLAMF7	NM_021181.3	CRACC,19A,CS1,CD319	GGGCACTATCATAGTGACCCAAAATCGTAATAGGGAGAGAGTAGACTTCCCAGATGGAGGCTACTCCCTGAAGCTCAGCAAACTGAAGAAGAATGACTCA	SLAM family member 7
SLC11A1	NM_000578.3	LSH,NRAMP,NRAMP1;solute carrier family 11 (proton-coupled divalent metal ion transporter), member 1;natural resistance-associated macrophage protein 1	TCACGTTCACCAGCATGCCCACCCTCATGCAGGAGTTTGCCAATGGCCTGCTGAACAAGGTCGTCACCTCTTCCATCATGGTGCTAGTCTGCGCCATCAA	solute carrier family 11 member 1
SLC16A1	NM_003051.3	solute carrier family 16 (monocarboxylic acid transporters), member 1,solute carrier family 16, member 1 (monocarboxylic acid transporter 1),solute carrier family 16 (monocarboxylate transporter), member 1;MCT,MCT1	TGGTGGCTGCTTGTCAGGCTGTGGCTTGATTGCAGCTTCTTTCTGTAACACCGTACAGCAACTATACGTCTGTATTGGAGTCATTGGAGGTCTTGGGCTT	solute carrier family 16 member 1
SL	NM	RDRC,M7V1;solute carrier family 1 (neutral amino acid transporter), member 5;AAAT,ASCT2	GGTGCCGCGCTCTTCCAGTGCGTG	solute carrier family 1 member 5

C1A5	_001145144.1		GCCGCAGTGTTCATTGCACAGCTCAGCCAGCAGTCCTTGGACTTCGTAAAGATCATCACCATCCTGGTCACGGCCA	
SLC2A1	NM_006516.2	GLUT1,GLUT,HTLVR,CSE;human T-cell leukemia virus (I and II) receptor,choreoathetosis/spasticity, episodic (paroxysmal choreoathetosis/spasticity),solute carrier family 2 (facilitated glucose transporter), member 1;DYT18,DYT9	ATGGCCGGGGTCCTATAAACGCTACGGTCCGCGCGCTCTCTGGCAAGAGGCAAGAGGTAGCAACAGCGAGCGTGCCGGTCGCTAGTCGCGGGTCCCCGAG	solute carrier family 2 member 1
SLC7A5	NM_003486.6	solute carrier family 7 (amino acid transporter light chain, L system), member 5;LAT1,E16,D16S469E,MPE16,CD98	TACTGAGACTTGGAACCTCATTGCTACCACAGACTTGCACTGAAGCCGGACAGCTGCCCAGACACATGGGCTTGTGACATTCGTGAAAACCAACCCTGTG	solute carrier family 7 member 5
SMAD5	NM_005903.5	MADH5;MAD, mothers against decapentaplegic homolog 5 (Drosophila),SMAD, mothers against DPP homolog 5 (Drosophila);Dwfc,JV5-1	AATTGGTTGGGTTTCTGAGGTGAAATCCAGAGTAAGAGTACTAGACAGTTCAACAAGCCACATCTAATGGCACAGATAGAGGATGTAGCTATTTTATACC	SMAD family member 5
SMAP1	NM_001044305.2	stromal membrane-associated protein 1,stromal membrane-associated GTPase-activating protein 1;FLJ13159,SMAP-1	GAAAAGCTGCAGAAGAAAGATCAGCAACTGGAGCCTAAAAAAAGTACCAGCCCTAAAAAAGCTGCGGAGCCCACTGTGGATCTTTTAGGACTTGATGGCC	small ArfGAP 1
SNAI1	NM_005985.3	snail 1 (drosophila homolog), zinc finger protein,snail homolog 1 (Drosophila),snail family zinc finger 1;SNA,SLUGH2,SNAH,SNAIL1,SNAIL	TTCTCACTGCCATGGAATTCCCTCCTGAGTGCCCCACTTCTGGCCACATCAGCCCCACAGGACTTTGATGAAGACCATTTTCTGGTTCTGTGTCCTCTGC	snail family transcriptional repressor 1
SNCA	NM_0011	PARK1,PARK4;Parkinson disease (autosomal dominant, Lewy body) 4,synuclein, alpha (non A4 component of amyloid precursor);NACP,PD1;non A4 component of amyloid precursor,alpha-synuclein,Î±-synuclein	ACCTGAAGCCTAAGAAATATCTTTGCTCCCAGTTTCTTGAGATCTGCTGACAGATGTTCCATCCTGTACAAGT	synuclein alpha

	46055.1		GCTCAGTTCCAATGTGCCCAGTCATGA	
SOCS1	NM_003745.1	SOCS-1,SSI-1,JAB,TIP3,Cish1	AGCTTAACTGTATCTGGAGCCAGGACCTGAACTCGCACCTCCTACCTCTTCATGTTTACATATACCCAGTATCTTTGCACAAACCAGGGGTTGGGGGAGG	suppressor of cytokine signaling 1
SOX10	NM_006941.3	SRY (sex determining region Y)-box 10,SRY-box 10;DOM,WS4,WS2E;dominant megacolon, mouse, human homolog of	GTTGCTGTCGGCAGGCTGAAGACACTAGAATCCTGACCTGTACATTCTGCCCTTGCCTCTTACCCCTTGCCTCCCAGTGGTATTTGAATAAAGTATGTAG	SRY-box transcription factor 10
SOX11	NM_003108.3	SRY (sex determining region Y)-box 11,SRY-box 11;SRY-related HMG-box gene 11	CCGCAGCGGAGGAGGTTTTCAGTGGCTGATTGAAACTCACTGCAAAATCACCACGACTCTTTCACCTACTGAGATGATTGACCGAGGTTTGGCCTTCCAT	SRY-box transcription factor 11
SOX2	NM_003106.2	SRY (sex determining region Y)-box 2,SRY-box 2	CTTAAGCCTTTCCAAAAAATAATAATAACAATCATCGGCGGCGGCAGGATCGGCCAGAGGAGGAGGGAAGCGCTTTTTTTGATCCTGATTCCAGTTTGCC	SRY-box transcription factor 2
SPIB	NM_003121.3	Spi-B transcription factor (Spi-1/PU.1 related);SPI-B	CTTTGTCATGTACAGACTCCCTGGGATCCTCATGTTTTGGGTGACAGGACCTATGGACCACTATACTCGGGGAGGCAGGGTAGCAGTTCTTCCAGAATCC	Spi-B transcription factor
SPP1	NM_000582.2	BNSP,OPN;osteopontin,bone sialoprotein I;BSPI,ETA-1;early T-lymphocyte activation 1	CGCCTTCTGATTGGGACAGCCGTGGGAAGGACAGTTATGAAACGAGTCAGCTGGATGACCAGAGTGCTGAAACCCACAGCCACAAGCAGTCCAGATTATA	secreted phosphoprotein 1
SPR	NM_03	sprouty homolog 4 (Drosophila)	CTCCCTTCCAACTGCATCAACTAACTCTCGGGGGTGTTCTGCTCACCAC	sprouty RTK signaling antagonist 4

Y4	09664.3		ACCGTCCTTCGGTTCTTACTGAGTCACAGACTCGCCTGCCCACTACGTGTC	
SREBF1	NM_001005291.1	SREBP1,bHLHd1,SREBP-1c,SREBP1a	TTCGCTTTCTGCAACACAGCAACCAGAAACTCAAGCAGGAGAACCTAAGTCTGCGCACTGCTGTCCACAAAAGCAAATCTCTGAAGGATCTGGTGTCGGC	sterol regulatory element binding transcription factor 1
SRP54	NM_001146282.1	signal recognition particle 54kDa	CCAGGCTAAGGCTTTTAAAGATAAAGTAGATGTAGCCTCAGTAATAGTGACAAAACTTGATGGCCATGCAAAAGGAGGTGGTGCACTCAGTGCAGTCGCT	signal recognition particle 54
STAT1	NM_007315.3	signal transducer and activator of transcription 1, 91kD,signal transducer and activator of transcription 1, 91kDa;STAT91,ISGF-3;transcription factor ISGF-3 components p91/p84	TCATTTGCTGTATGCCATCCTCGAGAGCTGTCTAGGTTAACGTTCGCACTCTGTGTATATAACCTCGACAGTCTTGGCACCTAACGTGCTGTGCGTAGCT	signal transducer and activator of transcription 1
STAT2	NM_005419.2	signal transducer and activator of transcription 2, 113kD,signal transducer and activator of transcription 2, 113kDa;STAT113	CCGTACACGAAGGAGGTGCTGCAGTCACTCCCGCTGACTGAAATCATCCGCCATTACCAGTTGCTCACTGAGGAGAATATACCTGAAAACCCACTGCGCT	signal transducer and activator of transcription 2
STAT3	NM_003150.3	signal transducer and activator of transcription 3 (acute-phase response factor);APRF	AATGCCACAGGCCACCTATAGCTACATACTCCTGGCATTGCACTTTTTAACCTTGCTGACATCCAAATAGAAGATAGGACTATCTAAGCCCTAGGTTTCT	signal transducer and activator of transcription 3
STAT4	NM_001243		ATCAGAGTGACAAGAATAGTGCCATGGTGAATCAGGAAGTTTTGACACTGCAGGAAATGCTTAACAGCCTCGATTTCAAGAGAA	signal transducer and activator of transcription 4

	835.1		AGGAGGCTCTCAGTAA	
STC1	NM_003155.2	STC	GGACCACTTACAGTGTGATAAGGCCTACTATACATTAGGAAGTGGCAGTTCTTTACTCGTCCCCTTTCATCGGTGCCTGGTACTCTGGCAAATGATGATG	stanniocalcin 1
SYK	NM_003177.5	spleen tyrosine kinase	TCCTACGCCCTGTGCCTGCTGCACGAAGGGAAGGTGCTGCACTATCGCATCGACAAAGACAAGACAGGGAAGCTCTCCATCCCCGAGGGAAAGAAGTTCG	spleen associated tyrosine kinase
TAF3	NM_031923.3	TAF3 RNA polymerase II, TATA box binding protein (TBP)-associated factor, 140 kD,TAF3 RNA polymerase II, TATA box binding protein (TBP)-associated factor, 140kDa;TAF140,TAFII140	CAAAAGATCCCACCAATGCTTTCTCCAGTCCATGTACAGGACAGTACAGACTTGGCACCTCCCTCACCCGAGCCGCCAATGTTGGCTCCAGTTGCAAAAT	TATA-box binding protein associated factor 3
TAP1	NM_000593.5	ABCB2;transporter 1, ATP-binding cassette, sub-family B (MDR/TAP);PSF1,RING4,D6S114E	TACCTTCACTCGAAACTTAACTCTCATGTCCATTCTCACCATAGCCAGTGCAGTGCTGGAGTTCGTGGGTGACGGGATCTATAACAACACCATGGGCCAC	transporter 1, ATP binding cassette subfamily B member
TAP2	NM_018833.2	ABCB3;transporter 2, ATP-binding cassette, sub-family B (MDR/TAP);PSF2,RING11,D6S217E	TTCCTTTAAATGCCAATGTGCTCTTGCGAAGCCTGGTGAAAGTGGTGGGGCTGTATGGCTTCATGCTCAGCATATCGCCTCGACTCACCCTCCTTTCTCT	transporter 2, ATP binding cassette subfamily B member
TAPBP	NM_003190.4	TAP binding protein (tapasin);TAPA;tapasin	CATGGGGCCCATGGACCGGAAATGGGACCTTCTGGCTGCCTACAGTTCAACCCTTTCAGGAGGGCACCTATCTGGCCACCATACACCTGCCATACCTGCA	TAP binding protein
TAPBPL	NM_0180	TAP binding protein-like;TAPBP-R,FLJ10143,TAPBPR	CACCCAGGTTGTCCCACCAGAGCGGAGAACAGCCTTGGGAGTCATCTTTGCCAGCAGTCTCTTCCTTCTTGCA	TAP binding protein like

	09.4		CTGATGTTCCTGGGGCTTCAGAGACGG	
TBX21	NM_013351.1	T-box 21;TBLYM,T-bet	CCGGGACCACAGCTATGAGGCTGAGTTTCGAGCAGTCAGCATGAAGCCTGCATTCTTGCCCTCTGCCCCTGGGCCCACCATGTCCTACTACCGAGGCCAG	T-box transcription factor 21
TBXAS1	NM_001061.4	thromboxane A synthase 1 (platelet, cytochrome P450, subfamily V),thromboxane A synthase 1 (platelet);CYP5,CYP5A1,THAS,TXS,TXAS,TS;cytochrome P450, family 5, subfamily A, polypeptide 1	GCCTGCGACCTTCTCCTGGCTCATTTAAAACGCTATGCGGAATCTGGGGACGCATTTGACATCCAGAGGTGCTACTGCAATTACACCACAGATGTGGTTG	thromboxane A synthase 1
TCF3	NM_003200.3	E2A,ITF1,MGC129647,MGC129648,bHLHb21,VDIR,E47,p75;transcription factor E2-alpha,immunoglobulin transcription factor 1,kappa-E2-binding factor,E2A immunoglobulin enhancer-binding factor E12/E47,VDR interacting repressor	GACCCCAGCCGGACCTTCAGCGAGGGCACCCACTTCACTGAGTCGCACAGCAGCCTCTCTTCATCCACATTCCTGGGACCGGGACTCGGAGGCAAGAGCG	transcription factor 3
TCL1A	NM_001098725.1	T cell leukemia/lymphoma 1A;TCL1	GATGGACGATACCGATCCTCAGACTCCAGTTTCTGGCGCTTAGTGTACCACATCAAGATTGACGGCGTGGAGGACATGCTTCTCGAGCTGCTGCCAGATG	TCL1 family AKT coactivator A
TDO2	NM_005651.3	TDO,TPH2	AAGCAAATCCTCTGGGAGTTGGATTCTGTTCGAGAGATCTTTCAGAATGGCCATGTCAGAGATGAAAGGAACATGCTTAAGGTTGTTTCTCGGATGCACC	tryptophan 2,3-dioxygenase
TGFB1	NM_000660.4	TGFB,DPD1;transforming growth factor, beta 1;CED,TGFbeta;Camurati-Engelmann disease,prepro-transforming growth factor beta-1,Diaphyseal dysplasia 1, progressive	TTTGATGTCACCGGAGTTGTGCGGCAGTGGTTGAGCCGTGGAGGGGAAATTGAGGGCTTTCGCCTTAGCGCCCACTGCTCCTGTGACAGCAGGGATAACA	transforming growth factor beta 1
TGF	NM_00	transforming growth factor, beta 2;prepro-transforming growth factor beta-2	AAAAGCCAGAGTGCCTGAACAACGGATTGAGCTATATCAGATTCTCAA	transforming growth factor beta 2

B2	1135599.3		GTCCAAAGATTTAACATCTCCAACCCAGCGCTACATCGACAGCAAAGTTGTG	
TGFB3	NM_001329938.1	ARVD1,ARVD;arrhythmogenic right ventricular dysplasia 1,transforming growth factor, beta 3;prepro-transforming growth factor beta-3	GAGAAGCAGGGGACAGAAGCAATGGCCGAGGCAGAAGACAAGCCGAGGTGCTGGTGACCCTGGGCGTCTGAGTGGATGATTGGGGCTGCTGCGCTCAGAG	transforming growth factor beta 3
TGFBR1	NM_001130916.2	MSSE,ESS1;multiple self-healing squamous epithelioma,transforming growth factor beta receptor I;ALK-5,ACVRLK4,ALK5,TBRI,TBR-i;activin A receptor type II-like kinase, 53kDa	ATCACTGTGATCTTATTCTGAGGGGAGAAAAAACTATCATAGCTCTGAGGCAAGACTTCGACTTTATAGTGCTATCAGTTCCCCGATACAGGGTCAGAGT	transforming growth factor beta receptor 1
TGFBR2	NM_001024847.2	MFS2;transforming growth factor, beta receptor II (70/80kDa),transforming growth factor beta receptor II;TBRII,TBR-ii	TAGCTCTGATGAGTGCAATGACAACATCATCTTCTCAGAAGAATATAACACCAGCAATCCTGACTTGTTGCTAGTCATATTTCAAGTGACAGGCATCAGC	transforming growth factor beta receptor 2
THBD	NM_000361.2	CD141	GACTCCTCCGGCCGTGGGGCTCGTGCATTCGGGCTTGCTCATAGGCATCTCCATCGCGAGCCTGTGCCTGGTGGTGGCGCTTTTGGCGCTCCTCTGCCAC	thrombomodulin
THBS1	NM_003246.2	TSP1,THBS,TSP,THBS-1,TSP-1;thrombospondin-1p180	TCAGTGTGGACTCCTAGAACGTGCGACCTGCCTCAAGAAAATGCAGTTTTCAAAAACAGACTCAGCATTCAGCCTCCAATGAATAAGACATCTTCCAAGC	thrombospondin 1

THY1	NM_006288.3	CD90	CCAGATCCAGGACTGAGATCCCAGAACCATGAACCTGGCCATCAGCATCGCTCTCCTGCTAACAGTCTTGCAGGTCTCCCGAGGGCAGAAGGTGACCAGC	Thy-1 cell surface antigen
TICAM1	NM_182919.3	toll-like receptor adaptor molecule 1;TRIF,TICAM-1,MGC35334,PRVTIRB;TIR domain-containing adapter molecule 1	CTTCATTTAGGACAAAACGGGCGCGATGATGCCCTGGCTTTCAGGGTGGTCAGAACTGGATACGGTGTTTACAATTCCAATCTCTCTATTTCTGGGTGAA	toll like receptor adaptor molecule 1
TIE1	NM_005424.4	TIE;tyrosine kinase with immunoglobulin and epidermal growth factor homology domains 1,tyrosine kinase with immunoglobulin-like and EGF-like domains 1;JTK14	TACGGGAACCTGCTAGATTTTCTGCGGAAAAGCCGGGTCCTAGAGACTGACCCAGCTTTTGCTCGAGAGCATGGGACAGCCTCTACCCTTAGCTCCCGGC	tyrosine kinase with immunoglobulin like and EGF like domains 1
TIGIT	NM_173799.2	VSIG9,VSTM3;VSIG9, VSTM3;FLJ39873,DKFZp667A205	TGGATCTTAGAAGACTTTTATCCTTCCACCATCTCTCTCAGAGGAATGAGCGGGGAGGTTGGATTTACTGGTGACTGATTTTCTTTCATGGGCCAAGGAA	T cell immunoreceptor with Ig and ITIM domains
TLR1	NM_003263.3	toll-like receptor 1;rsc786,KIAA0012,CD281	AAAATTTCTGGGGTTGAGCACCACACACTTAGAAAAATCTAGTGTGCTGCCAATTGCTCATTTGAATATCAGCAAGGTCTTGCTGGTCTTAGGAGAGACT	toll like receptor 1
TLR2	NM_001318787.1	toll-like receptor 2;TIL4,CD282	CCACATACTTTGTGGATGGTGTGGGTCTTGGGGGTCATCATCAGCCTCTCCAAGGAAGAATCCTCCAATCAGGCTTCTCTGTCTTGTGACCGCAATGGTA	toll like receptor 2
TLR3	NM_00326	toll-like receptor 3;CD283	CTGATGAAATGTCTGGATTTGGACTAAAGAAAAAAGGAAAGGCTAGCAGTCATCCAACAGAATCATGAGACAGACTTTGCCTTG	toll like receptor 3

	5.2		TATCTACTTTTGGGGG	
TLR4	NR_024168.1	toll-like receptor 4;hToll,CD284,TLR-4,ARMD10	TCATCCATGAAGGTTTCCATAAAAGCCGAAAGGTGATTGTTGTGGTGTCCCAGCACTTCATCCAGAGCCGCTGGTGTATCTTTGAATATGAGATTGCTCA	toll like receptor 4
TLR5	NM_003268.5	SLEB1;systemic lupus erythematosus susceptibility 1,toll-like receptor 5;TIL3,FLJ10052,MGC126430,MGC126431;Toll/interleukin-1 receptor-like protein 3	ATTGTATGCACTGTCACTCTGACTCTGTTCCTCATGACCATCCTCACAGTCACAAAGTTCCGGGGCTTCTGTTTTATCTGTTATAAGACAGCCCAGAGAC	toll like receptor 5
TLR7	NM_016562.3	toll-like receptor 7	TGTGGGCACCACACAGGTGGTTGCTGCTTCAGTGCTTCCTGCTCTTTTTCCTTGGGCCTGCTTCTGGGTTCCATAGGGAAACAGTAAGAAAGAAAGACAC	toll like receptor 7
TLR8	NM_138636.4	toll-like receptor 8;CD288	TGACTTTACATCTTCCCTTCGGACACTGCTGCTGAGTCATAACAGGATTTCCCACCTACCCTCTGGCTTTCTTTCTGAAGTCAGTAGTCTGAAGCACCTC	toll like receptor 8
TLR9	NM_017442.2	toll-like receptor 9;CD289	ACCTTCTTGGCTGTGCCCACCCTGGAAGAGCTAAACCTGAGCTACAACAACATCATGACTGTGCCTGCGCTGCCCAAATCCCTCATATCCCTGTCCCTCA	toll like receptor 9
TMEM140	NM_018295.3	FLJ11000	CTCTGGGAGGCTGGCAACCTCACTGACCTGCCCAACCTGAGAATCGGCTTCTATAACTTCTGCCTGTGGAATGAGGACACCAGCACCCTACAGTGTCACC	transmembrane protein 140
TMEM173	NM_198282.8	TMEM173;transmembrane protein 173;FLJ38577,NET23,ERIS,MPYS,STING,MITA;stimulator of interferon genes,endoplasmic reticulum IFN stimulator	CTGGCATGGTCATATTACATCGGATATCTGCGGCTGATCCTGCCAGAGCTCCAGGCCCGGATTCGAACTTACAATCAGCATTAC	stimulator of interferon response cGAMP interactor 1

	2.1		AACAACCTGCTACGGG	
TNF	NM_000594.3	TNFA;tumor necrosis factor (TNF superfamily, member 2);TNFSF2,DIF,TNF-alpha;TNF superfamily, member 2	TATTTACAGATGAATGTATTTATTTGGGAGACCGGGGTATCCTGGGGGACCCAATGTAGGAGCTGCCTTGGCTCAGACATGTTTTCCGTGAAAACGGAGC	tumor necrosis factor
TNFAIP3	NM_001270508.1	tumor necrosis factor, alpha-induced protein 3;A20,OTUD7C	TCAGGGAAAATGGACGTATTCAGAGAGTGTTTGTAGTTCATGGTTTTTCCCTACCTGCCCGGTTCCTTTCCTGAGGACCCGGCAGAAATGCAGAACCATC	TNF alpha induced protein 3
TNFAIP6	NM_007115.3	tumor necrosis factor, alpha-induced protein 6;TSG6,TSG-6	CTTGAACGAGCAGCCGGTGTGTACCACAGAGAAGCACGGTCTGGCAAATACAAGCTCACCTACGCAGAAGCTAAGGCGGTGTGTGAATTTGAAGGCGGCC	TNF alpha induced protein 6
TNFRSF10B	NM_003842.3	tumor necrosis factor receptor superfamily, member 10b;DR5,KILLER,TRICK2A,TRAIL-R2,TRICKB,CD262,TRAILR2	GCATCTCCTGCAAATATGGACAGGACTATAGCACTCACTGGAATGACCTCCTTTTCTGCTTGCGCTGCACCAGGTGTGATTCAGGTGAAGTGGAGCTAAG	TNF receptor superfamily member 10b
TNFRSF10C	NM_003841.3	tumor necrosis factor receptor superfamily, member 10c, decoy without an intracellular domain;DcR1,TRAILR3,LIT,TRID,CD263	CCGGAAGTGTAGCAGGTGCCCTAGTGGGGAAGTCCAAGTCAGTAATTGTACGTCCTGGGATGATATCCAGTGTGTTGAAGAATTTGGTGCCAATGCCACT	TNF receptor superfamily member 10c
TNFRSF10D	NM_003840.4	tumor necrosis factor receptor superfamily, member 10d, decoy with truncated death domain;DcR2,TRUNDD,TRAILR4,CD264	AGGAGGTCCCGAACGTGTGCACAGAGTCCTTTTCCGGCGGCGTTCATGTCCTTCACGAGTTCCTGGGGCGGAGGACAATGCCCGCAACGAGACCCTGAGT	TNF receptor superfamily member 10d
TNFRS	NM_01	PDB2,LOH18CR1;tumor necrosis factor receptor superfamily, member 11a, activator of NFKB,Paget disease of bone 2,loss of heterozygosity, 18, chromosomal region 1,tumor necrosis factor	CAGTGTGTGTTCATTGTAAACACTTTTGGGAAAGGGCTAAACATGTGAGGCCTGGAGATA	TNF receptor superfamily member 11a

F11A	270949.1	receptor superfamily, member 11a, NFKB activator;RANK,CD265,FEO	GTTGCTAAGTTGCTAGGAACATGTGGTGGGACTTTCATAT	
TNFRSF11B	NM_002546.3	OPG;osteoprotegerin,tumor necrosis factor receptor superfamily, member 11b;OCIF,TR1	AGCCCCGTGTGCAAGGAGCTGCAGTACGTCAAGCAGGAGTGCAATCGCACCCACAACCGCGTGTGCGAATGCAAGGAAGGGCGCTACCTTGAGATAGAGT	TNF receptor superfamily member 11b
TNFRSF14	NM_003820.3	tumor necrosis factor receptor superfamily, member 14 (herpesvirus entry mediator),tumor necrosis factor receptor superfamily, member 14;HVEM,ATAR,TR2,LIGHTR,HVEA,CD270;herpesvirus entry mediator	GGAGCTGACGGGCACAGTGTGTGAACCCTGCCCTCCAGGCACCTACATTGCCCACCTCAATGGCCTAAGCAAGTGTCTGCAGTGCCAAATGTGTGACCCA	TNF receptor superfamily member 14
TNFRSF17	NM_001192.2	BCMA;tumor necrosis factor receptor superfamily, member 17;BCM,CD269,TNFRSF13A	TCTAATACTCCTCCTCTAACATGTCAGCGTTATTGTAATGCAAGTGTGACCAATTCAGTGAAAGGAACGAATGCGATTCTCTGGACCTGTTTGGGACTGA	TNF receptor superfamily member 17
TNFRSF18	NM_148901.1	tumor necrosis factor receptor superfamily, member 18;AITR,GITR,CD357	TGCTGCCGGGTTCACACGACGCGCTGCTGCCGCGATTACCCGGGCGAGGAGTGCTGTTCCGAGTGGGACTGCATGTGTGTCCAGCCTGAATTCCACTGCG	TNF receptor superfamily member 18
TNFRSF1A	NM_001065.2	TNFR1;tumor necrosis factor receptor superfamily, member 1A;TNF-R,TNFAR,TNFR60,TNF-R-I,CD120a,TNF-R55	ACGGACTGCAGGGAGTGTGAGAGCGGCTCCTTCACCGCTTCAGAAAACCACCTCAGACACTGCCTCAGCTGCTCCAAATGCCGAAAGGAAATGGGTCAGG	TNF receptor superfamily member 1A
TNFRSF1B	NM_001066.2	TNFR2;tumor necrosis factor receptor superfamily, member 1B;TNFBR,TNFR80,TNF-R75,TNF-R-II,p75,CD120b	TCCAACACGACTTCATCCACGGATATTTGCAGGCCCCACCAGATCTGTAACGTGGTGGCCATCCCTGGGAATGCAAGCATGGATGCAGTCTGCACGTCCA	TNF receptor superfamily member 1B
TNF	NM_1	TNFRSF12;tumor necrosis factor receptor superfamily, member 12 (translocating chain-association membrane protein),tumor necrosis	GCCCGGGCCCAGGGCGGCACTCGTAGCCCCAGGTGT	TNF receptor superfamily member 25

RSF25	48970.1	factor receptor superfamily, member 25;DR3,TRAMP,WSL-1,LARD,WSL-LR,DDR3,TR3,APO-3	GACTGTGCCGGTGACTTCCACAAGAAGATTGGTCTGTTTTGTTGCAGAGGCTGCCCAGCGGATG	
TNFRSF4	NM_003327.3	TXGP1L;tumor necrosis factor receptor superfamily, member 4;ACT35,OX40,CD134	CAACTCTGCACCGTTCTAGGTGCCGATGGCTGCCTCCGGCTCTCTGCTTACGTATGCCATGCATACCTCCTGCCCCGCGGGACCACAATAAAAACCTTGG	TNF receptor superfamily member 4
TNFRSF8	NM_001243.3	CD30,D1S166E;tumor necrosis factor receptor superfamily, member 8;KI-1	CCCGGCATGTTCTGTTCCACGTCTGCCGTCAACTCCTGTGCCCGCTGCTTCTTCCATTCTGTCTGTCCGGCAGGGATGATTGTCAAGTTCCCAGGCACGG	TNF receptor superfamily member 8
TNFRSF9	NM_001561.5	ILA;tumor necrosis factor receptor superfamily, member 9;CD137,4-1BB	GAACAGGATTGTAAACAAGGTCAAGAACTGACAAAAAAAGGTTGTAAAGACTGTTGCTTTGGGACATTTAACGATCAGAAACGTGGCATCTGTCGACCCT	TNF receptor superfamily member 9
TNFSF10	NM_003810.3	tumor necrosis factor (ligand) superfamily, member 10;TRAIL,Apo-2L,TL2,CD253	CCTGGGACAGACCTGCGTGCTGATCGTGATCTTCACAGTGCTCCTGCAGTCTCTCTGTGTGGCTGTAACTTACGTGTACTTTACCAACGAGCTGAAGCAG	TNF superfamily member 10
TNFSF12	NM_003809.2	tumor necrosis factor (ligand) superfamily, member 12;TWEAK,DR3LG,APO3L	GCGCCTTTCCTGAACCGACTAGTTCGGCCTCGCAGAAGTGCACCTAAAGGCCGGAAAACACGGGCTCGAAGAGCGATCGCAGCCCATTATGAAGTTCATC	TNF superfamily member 12
TNFSF13	NM_003808.3	tumor necrosis factor (ligand) superfamily, member 13;APRIL,CD256	AGTCAGAGAGCCGGCACTCTCAGTTGCCCTCTGGTTGAGTTGGGGGGCAGCTCTGGGGGCCGTGGCTTGTGCCATGGCTCTGCTGACCCAACAAACAGAG	TNF superfamily member 13
TNFS	NM_0	TNFSF20;tumor necrosis factor (ligand) superfamily, member 13b;BAFF,THANK,BLYS,TALL-1,TALL1,CD257;B-cell-activating factor,TNF and ApoL-related leukocyte expressed ligand 1,TNF	AGGGGTGAGCCAAGCCCTGCCATGTAGTGCACGCAGGACATCAACAA	TNF superfamily member 13b

F13B	1145645.2	homolog that activates apoptosis,B-lymphocyte stimulator	ACACAGATAACAGGAAATGATCCATTCCCTGTGGTCACTTATTCTAAAGGCCC	
TNFSF18	NM_005092.3	tumor necrosis factor (ligand) superfamily, member 18;AITRL,TL6,hGITRL	ATTTAATTTATGGCCAAGTGGCTCCCAATGCAAACTACAATGATGTAGCTCCTTTTGAGGTGCGGCTGTATAAAAACAAAGACATGATACAAACTCTAAC	TNF superfamily member 18
TNFSF4	NM_001297562.1	TXGP1;tax-transcriptionally activated glycoprotein 1, 34kD,tumor necrosis factor (ligand) superfamily, member 4;OX-40L,gp34,CD252	CAAAAGGAGGATGAAATCATGAAGGTGCAGAACAACTCAGTCATCATCAACTGTGATGGGTTTTATCTCATCTCCCTGAAGGGCTACTTCTCCCAGGAAG	TNF superfamily member 4
TNFSF8	NM_001244.3	CD30LG;tumor necrosis factor (ligand) superfamily, member 8;CD153	CAGCTATTTCTATTTGACCACAGCCACTCTGGCTCTGTGCCTTGTCTTCACGGTGGCCACTATTATGGTGTTGGTCGTTCAGAGGACGGACTCCATTCCC	TNF superfamily member 8
TNFSF9	NM_003811.3	tumor necrosis factor (ligand) superfamily, member 9;4-1BB-L,4-1BBL;receptor 4-1BB ligand,homolog of mouse 4-1BB-L	CCGAGACTCCGCGAGGGTCCCGAGCTTTCGCCCGACGATCCCGCCGGCCTCTTGGACCTGCGGCAGGGCATGTTTGCGCAGCTGGTGGCCCAAAATGTTC	TNF superfamily member 9
TNKS	NM_003747.2	tankyrase, TRF1-interacting ankyrin-related ADP-ribose polymerase;TIN1,TINF1,TNKS1,PARP-5a,PARP5A,pART5,ARTD5;TRF1-interacting ankyrin-related ADP-ribose polymerase 1	TGATGTTGATTATCGACTCTTAGAGGCATCTAAAGCTGGAGACTTGGAAACTGTGAAGCAACTTTGCAGCTCTCAAAATGTGAATTGTAGAGACTTAGAG	tankyrase
TP53	NM_00054	p53,LFS1;Li-Fraumeni syndrome	GGGGAGCAGGGCTCACTCCAGCCACCTGAAGTCCAAAAAGGGTCAGTCTACCTCCCGCCATAAAAAACTCATGTTCAAGACAG	tumor protein p53

	6.2		AAGGGCCTGACTCAGAC	
TPI1	NM_000365.5		AGGTGGTTTGTGCTCCCCCTACTGCCTATATCGACTTCGCCCGGCAGAAGCTAGATCCCAAGATTGCTGTGGCTGCGCAGAACTGCTACAAAGTGACTAA	triosephosphate isomerase 1
TPM1	NM_000366.5	C15orf13,CMH3;chromosome 15 open reading frame 13,cardiomyopathy, hypertrophic 3,tropomyosin 1 (alpha)	TACTCGCAGAAGGAAGACAGATATGAGGAAGAGATCAAGGTCCTTTCCGACAAGCTGAAGGAGGCTGAGACTCGGGCTGAGTTTGCGGAGAGGTCAGTAA	tropomyosin 1
TPSAB1/B2	NM_003294.3	TPSB1,TPS1,TPS2;tryptase beta 1;tryptase alpha II,tryptase beta I,tryptase-I,tryptase-II,tryptase-III	CTCCCACCGCCATTTCCTCTGAAGCAGGTGAAGGTCCCCATAATGGAAAACCACATTTGTGACGCAAAATACCACCTTGGCGCCTACACGGGAGACGACG	TPSB2 (NM_024164), TPSAB1 (NM_003294)
TRAF1	NM_001190945.1	EBI6	CTTCAAGGGAAGCCCACAGTCTGTGCAAGAGCATGAGGTCACCTCCCAGACCTCCCACCTAAACCTGCTGTTGGGGTTCATGAAACAGTGGAAGGCCCGG	TNF receptor associated factor 1
TRAT1	NM_016388.3	TCRIM;TCRIM;HSPC062,TRIM	GTTAAGGGGAAGCGTAGAAAGCCCAGGAAACAGAATACTCATTTCTCAGACAAGGATGGAGATGAGCAACTACATGCAATAGATGCCAGCGTTTCTAAGA	T cell receptor associated transmembrane adaptor 1
TREM1	NM_001242589.2	TREM-1,CD354	CAGGCTCTGGGGGCTGCTGTGGATGCTCTTTGTCTCAGAACTCCGAGCTGCAACTAAATTAACTGAGGAAAAGTATGAACTGAAAGAGGGGCAGACCCTG	triggering receptor expressed on myeloid cells 1
TR	NM	triggering receptor expressed on myeloid cells 2a;TREM-2,Trem2a,Trem2b,Trem2c	CAGCCTGCATACTTGCCACTTGGC	triggering receptor expressed on myeloid cells 2

EM2	_018965.3		CACCAGGACTCCTTGTTCTGCTCTGGCAAGAGACTACTCTGCCTGAACACTGCTTCTCCTGGACCCTGGAAGCAGG	
TRIM21	NM_031141.3	SSA1;Sjogren syndrome antigen A1 (52kDa, ribonucleoprotein autoantigen SS-A/Ro),tripartite motif-containing 21;RNF81,RO52;Ro/SSA 52kDa,E3 ubiquitin-protein ligase TRIM21	AGAGACTCTGTGCGCAGGAAGGGGCACTTTTTGCTTAGTTCCAAGAGTGGCTTCTGGACAATTTGGTTGTGGAACAAACAAAAATATGAGGCTGGCACCT	tripartite motif containing 21
TSLP	NM_033035.4		TGACTGGCTTCATGGCAGTAATTCTCGGCTGTAGTTGCATAAGCATTGCTCAAGAGGAAAATCCAAAAGTGCAGCAGGAGAACTCTTTTCCCTGAAAAAG	thymic stromal lymphopoietin
TTC30A	NM_152275.3	FLJ13946,IFT70A,FAP259	TGCCCTCAAGCAACAATTGCTAGAGTAACATCTTTGTATAAGCAAGTAACCCCAGATAGAGTTGACGTTTCAGCTTTGGGCTGTCAAAAGGGTATGTCAT	tetratricopeptide repeat domain 30A
TWF1	NM_002822.4	PTK9;protein tyrosine kinase 9,PTK9 protein tyrosine kinase 9,twinfilin, actin-binding protein, homolog 1 (Drosophila);A6	CCAGGCTAGAGTATTTTCTAAGCTGGAAACTTAGTGTGCCTTGGAAAAGGCCGCAAGTTGCTTACTCCGAGTAGCTGTGCTAGCTCTGTCAGACTGTAGG	twinfilin actin binding protein 1
TWIST1	NM_000474.3	ACS3,BPES3,TWIST,CRS;blepharophimosis, epicanthus inversus and ptosis 3,acrocephalosyndactyly 3,twist homolog 1 (Drosophila),twist basic helix-loop-helix transcription factor 1,craniosynostosis;SCS,H-twist,BPES2,bHLHa38,CRS1;Saethre-Chotzen syndrome	GCGCCCCGCTCTTCTCCTCTGCCCCGGGCCCGCGAGGCCACGCGTCGCCGCTCGAGAGATGATGCAGGACGTGTCCAGCTCGCCAGTCTCGCCGGCCGAC	twist family bHLH transcription factor 1
TWIST2	NM_001271893.3	twist homolog 2 (Drosophila),twist basic helix-loop-helix transcription factor 2;DERMO1,Dermo-1,bHLHa39	TCTCCGTGTGGCGCATGGAGGGCGCGTGGTCCATGTCCGCCTCCCACTAGCGCCGCGCCACCCACCTCCGGACCGGCGCGCCAGGGCTGTCCGTCGCGTC	twist family bHLH transcription factor 2

TYMP	NM_001113755.2	MNGIE,ECGF1;endothelial cell growth factor 1 (platelet-derived);gliostatin	CTGGGGCACACAGGAGGCACCTTGGATAAGCTGGAGTCTATTCCTGGATTCAATGTCATCCAGAGCCCAGAGCAGATGCAAGTGCTGCTGGACCAGGCGG	thymidine phosphorylase	
TYMS	NM_001071.1	TS;Tsase,TMS,HsT422	TGCTAAAGAGCTGTCTTCCAAGGGAGTGAAAATCTGGGATGCCAATGGATCCCGAGACTTTTTGGACAGCCTGGGATTCTCCACCAGAGAAGAAGGGGAC	thymidylate synthetase	
UBA7	NM_003335.2	UBE1L;ubiquitin-activating enzyme E1-like,ubiquitin-like modifier activating enzyme 7;D8,UBE2,UBA1B;UBA1, ubiquitin-activating enzyme E1 homolog B (yeast),UBA7, ubiquitin-activating enzyme E1	CCTTGTGTCATAAGCATGGAGTTTGCTTTCTGGCGGCTGACACCCGGGGCCTCGTGGGGCAGTTGTTCTGTGACTTTGGTGAGGACTTCACTGTGCAGGA	ubiquitin like modifier activating enzyme 7	
UBE2C	NM_007019.2	ubiquitin-conjugating enzyme E2C;UBCH10	GTCTGCCCTGTATGATGTCAGGACCATTCTGCTCTCCATCCAGAGCCTTCTAGGAGAACCCAACATTGATAGTCCCTTGAACACACATGCTGCCGAGCTC	ubiquitin conjugating enzyme E2 C	
UBE2T	NM_014176.1	ubiquitin-conjugating enzyme E2T;HSPC150,FANCT	GTGTCAGCTCAGTGCATCCCAGGCAGCTCTTAGTGTGGAGCAGTGAACTGTGTGTGGTTCCTTCTACTTGGGGATCATGCAGAGAGCTTCACGTCTGAAG	ubiquitin conjugating enzyme E2 T	
ULBP2	NM_025217.2	RAET1H	TGCCCACGACCTACGGTGTATGTCCAGTGGCCTCCAGCAGATCATGATGACATCATGGACCCAATAGCTCATTCACTGCCTTGATTCCTTTTGCCAACAA	UL16 binding protein 2	RAET1L (NM_130900)
VCAM1	NM_00107	CD106	CAGACTTCCCTGAATGTATTGAACTTGGAAAGAAATGCCCATCTATGTCCCTTGCTGTGAGCAAGAAGTCAAAGTAAAACTTGC	vascular cell adhesion molecule 1	

	8.3		TGCCTGAAGAACAGTA	
VCAN	NM_001126336.2	CSPG2;chondroitin sulfate proteoglycan 2;PG-M;versican proteoglycan	AACCTTAATAGTAACCCATGCGCTACATAAAGTCAAAGTGGGAAAAAGCCCACCGGTGAGGGGCTCCCTCTCTGGAAAAGTCAGCCTACCTTGTCATTTT	versican
VEGFA	NM_001171623.1	VEGF;vascular endothelial growth factor;VEGF-A,VPF	TATTTGACTGCTGTGGACTTGAGTTGGGAGGGGAATGTTCCCACTCAGATCCTGACAGGGAAGAGGAGGAGATGAGAGACTCTGGCATGATCTTTTTTTT	vascular endothelial growth factor A
VEGFB	NM_003377.3	VRF;VEGFL	TGCCGGAAGCTGCGAAGGTGACACATGGCTTTTCAGACTCAGCAGGGTGACTTGCCTCAGAGGCTATATCCCAGTGGGGGAACAAAGAGGAGCCTGGTAA	vascular endothelial growth factor B
VEGFC	NM_005429.4	VRP,VEGF-C;vascular endothelial growth factor-related protein	CAAGCAAAGATCTGGAGGAGCAGTTACGGTCTGTGTCCAGTGTAGATGAACTCATGACTGTACTCTACCCAGAATATTGGAAAATGTACAAGTGTCAGCT	vascular endothelial growth factor C
VHL	NM_198156.1	von Hippel-Lindau syndrome,von Hippel-Lindau tumor suppressor, E3 ubiquitin protein ligase;VHL1	GAGCCCTCCCAGGTCATCTTCTGCAATCGCAGTCCGCGCGTCGTGCTGCCCGTATGGCTCAACTTCGACGGCGAGCCGCAGCCCTACCCAACGCTGCCGC	von Hippel-Lindau tumor suppressor
VSIR	NM_022153.1	C10orf54;chromosome 10 open reading frame 54;SISP1,GI24,B7-H5,B7H5,VISTA,PD-1H,Dies1;stress induced secreted protein 1,V-domain Ig suppressor of T cell activation,PDCD1 homolog	ATCCCTGCTCTTCGCTCTCTTCCTGGCTGCGTCCCTAGGTCCGGTGGCAGCCTTCAAGGTCGCCACGCCGTATTCCCTGTATGTCTGTCCCGAGGGGCAG	V-set immunoregulatory receptor
VT	NM	B7-H4,FLJ22418,B7S1,B7x,B7H4;B7 family member, H4,B7 superfamily member 1	AATGACATTGCCAAAGCAACAGG	V-set domain containing T cell activation inhibitor 1

CN1	_001253849.1		GGATATCAAAGTGACAGAATCGGAGATCAAAAGGCGGAGTCACCTACAGCTGCTAAACTCAAAGGCTTCTCTGTGTG	
WDR76	NM_024908.3	FLJ12973	CGTTTGGTGGAGAATACCTTGTCTCTGTGTGTTCCATCAATGCCATGCACCCAACTCGGTATATTTTGGCTGGAGGTAATTCCAGCGGGAAGATACATGT	WD repeat domain 76
WNT10A	NM_025216.2	wingless-type MMTV integration site family, member 10A	CGGTCTTGGCAAGGCAGCATCGCCTTGGCTCTTGGGAAGAGGAGATTGGACCACATGATCTTATAGGAACCCCTCAGCTCTGAGGTCTGTGATCGCCGGA	Wnt family member 10A
WNT11	XM_011545241.2	wingless-type MMTV integration site family, member 11	CTCTGCTTGTGAATTCCAGATGCCAGGCATGGGAGGCGGCTTGTGCTTTGCCTTCACTTGGAAGCCACCAGGAACAGAAGGTCTGGCCACCCTGGAAGGA	Wnt family member 11
WNT2	NM_003391.2	INT1L1;wingless-type MMTV integration site family member 2;IRP;secreted growth factor	GATGTAACAAGGTGGGGACGTGTGTCCTTTGGTACTATGGTGTGTTGTATCTTTGTAAGAGCAAAAGCCTCAGAAAGGGATTGCTTTGCATTACTGTCCC	Wnt family member 2
WNT2B	NM_001291880.1	WNT13;wingless-type MMTV integration site family, member 2B;XWNT2;XWNT2, Xenopus, homolog of,wingless-type MMTV integration site family, member 13	CGAGAATGGATCCGAGAGTGTCAGCACCAATTCCGCCACCACCGCTGGAACTGTACCACCCTGGACCGGGACCACACCGTCTTTGGCCGTGTCATGCTCA	Wnt family member 2B
WNT	NM_0	wingless-type MMTV integration site family, member 3A	GTCGGCCTTTGTCCACGCCATTGCCTCAGCCGGTGT	Wnt family

3A	33131.3		GGCCTTTGCAGTGACACGCTCATGTGCAGAAGGCACGGCCGCCATCTGTGGCTGCAGCAGCCGC	member 3A
WNT4	NM_030761.4	wingless-type MMTV integration site family, member 4;WNT-4	TCCAAGCTCCTGGCTCCTGTGTTCAGTGGCTGAATTCAACGTCCGAGATTCGGAATATGGGCTCCAGCCTGCAGAAACTCTTCTCCAGGCATTGTGGTTT	Wnt family member 4
WNT5A	NM_003392.3	wingless-type MMTV integration site family, member 5A;hWNT5A;WNT-5A protein	GAGGCCGGCCGCAGGACGGTGTACAACCTGGCTGATGTGGCCTGCAAGTGCCATGGGGTGTCCGGCTCATGTAGCCTGAAGACATGCTGGCTGCAGCTGG	Wnt family member 5A
WNT5B	NM_030775.2	wingless-type MMTV integration site family, member 5B	CCGAGATGTTTATCATCGGTGCCCAGCCCGTGTGCAGTCAGCTTCCCGGGCTCTCCCCTGGCCAGAGGAAGCTGTGCCAATTGTACCAGGAGCACATGGC	Wnt family member 5B
WNT7B	NM_058238.2	wingless-type MMTV integration site family, member 7B	TGGGCCCTGGGGCGGTGGGGACAGATGTTGACACAAATTATTTATGTTTTCTTAGTATCAGAAGAGGATTCTCGGCACTAACACATAGCCAGTCCTAACT	Wnt family member 7B
XCL1/2	NM_002995.2	SCYC2;small inducible cytokine subfamily C, member 2,chemokine (C motif) ligand 2;SCM-1b	CCTCACTACCCAGCGACTGCCGGTTAGCAGAATCAAGACCTACACCATCACGGAAGGCTCCTTGAGAGCAGTAATTTTTATTACCAAACGTGGCCTAAAA	X-C motif chemokine ligand 2
ZAP70	NM_001079.3	SRK;SRK;ZAP-70,STD;tyrosine-protein kinase ZAP-70	GGAGCTCAAGGACAAGAAGCTCTTCCTGAAGCGCGATAACCTCCTCATAGCTGACATTGAACTTGGCTGCGGCAACTTTGGCTCAGTGCGCCAGGGCGTG	zeta chain of T cell receptor associated protein kinase 70
ZC3H	NM_02	FLJ23231,MCPIP1,Regnase-1;MCP induced protein 1	CAGTGGCAGCAGCTTTGGGCCCACAGACTGGCTCCCACAGACGCTGGA	zinc finger CCCH-type containing 12A

12A	5079.2		CTCACTCCCGTACGTCTCCCAGGATTGCCTGGACTCGGGCATTGGCTCCCTG	
ZEB1	NM_001128128.2	TCF8,PPCD3;transcription factor 8 (represses interleukin 2 expression),posterior polymorphous corneal dystrophy 3;BZP,ZEB,AREB6,NIL-2-A,Zfhep,Zfhx1a,FECD6	CAAAGTAAATATCCCTGCCAAGAACAATGATCAGCCTCAATCTGCAAATGCAAATGAACCCCAGGACAGCACAGTAAATCTACAAAGTCCTTTGAAGATG	zinc finger E-box binding homeobox 1
ZEB2	NM_001171653.1	ZFHX1B;zinc finger homeobox 1b;KIAA0569,SIP-1,SIP1;SMAD interacting protein 1	ACTGCAAGGCTGAAGAAATTTCAAAGTTGAAAGGTTATCACATGAAGGATCCATGCTCTCAACCTGAGGAACAAGGAGTTACTTCTCCTAATATTCCGCC	zinc finger E-box binding homeobox 2
**Internal Reference Genes**				
ABCF1	NM_001090.2	ABC50;ATP-binding cassette, sub-family F (GCN20), member 1;EST123147	TCCCGCCAAGCCATGTTAGAAAATGCATCTGACATCAAGCTGGAGAAGTTCAGCATCTCCGCTCATGGCAAGGAGCTGTTCGTCAATGCAGACCTGTACA	ATP binding cassette subfamily F member 1
DNAJC14	NM_032364.5	DnaJ (Hsp40) homolog, subfamily C, member 14;DNAJ,DRIP78,HDJ3,LIP6,FLJ32792	GGAGACAGCGGCACCGCTTTCCAACGAAGGAGGATACTCGGGAGGGTGGACGTAGGGATCCCAGGTCCCCTGGTCGACATCGGCTGGGTCGGAAACGAAG	DnaJ heat shock protein family (Hsp40) member C14
ERCC3	NM_000122.1	excision repair cross-complementing rodent repair deficiency, complementation group 3,excision repair cross-complementation group 3;XPB,BTF2,RAD25;xeroderma pigmentosum group B complementing	CTGGTGCCCTATGTCTCCTGAATTTTACCGGGAATATGTGGCAATCAAAACCAAGAAACGAATCTTGCTGTACACCATGAACCCCAACAAATTTAGAGCT	ERCC excision repair 3, TFIIH core complex helicase subunit
G6PD	NM_00	G6PD1	ACATCGCCTGCGTTATCCTCACCTTCAAGGAGCCCTTTGGCACTGAGG	glucose-6-phosphate dehydrogenase

	0402.4		GTCGCGGGGGCTATTTCGATGAATTTGGGATCATCCGGGACGTGATGCAGAA	
GUSB	NM_000181.1	glucuronidase, beta	CGGTCGTGATGTGGTCTGTGGCCAACGAGCCTGCGTCCCACCTAGAATCTGCTGGCTACTACTTGAAGATGGTGATCGCTCACACCAAATCCTTGGACCC	glucuronidase beta
MRPL19	NM_014763.3	MRP-L15,RPML15,KIAA0104,RLX1;39S ribosomal protein L19	ACAGCTGACCCATATGCCAGTGGAAAAATCAGCCAGTTTCTGGGGATTTGCATTCAGAGATCAGGAAGAGGACTTGGAGCTACTTTCATCCTTAGGAATG	mitochondrial ribosomal protein L19
NRDE2	NM_017970.3	C14orf102;chromosome 14 open reading frame 102;FLJ14051	TGGAGGTCCTATGTACAGATTCAGAATAAGTCCCACAGTGCCAGCAAAACCAGGAGATTTTTTGACACAATCACCAGGTCTGCCAAACCCTTGGAGCCTT	NRDE-2, necessary for RNA interference, domain containing
OAZ1	NM_004152.2	OAZ;AZI,MGC138338,AZ1;antizyme 1	GGTGGGCGAGGGAATAGTCAGAGGGATCACAATCTTTCAGCTAACTTATTCTACTCCGATGATCGGCTGAATGTAACAGAGGAACTAACGTCCAACGACA	ornithine decarboxylase antizyme 1
POLR2A	NM_000937.2	POLR2;polymerase (RNA) II (DNA directed) polypeptide A, 220kDa;POLRA,RPB1;DNA-directed RNA polymerase II largest subunit, RNA polymerase II 220 kd subunit,RNA polymerase II subunit B1	TTCCAAGAAGCCAAAGACTCCTTCGCTTACTGTCTTCCTGTTGGGCCAGTCCGCTCGAGATGCTGAGAGAGCCAAGGATATTCTGTGCCGTCTGGAGCAT	RNA polymerase II subunit A
PSMC4	NM_006503.2	MIP224;proteasome (prosome, macropain) 26S subunit, ATPase, 4;TBP7,S6,MGC8570,MGC13687,MGC23214,TBP-7;protease 26S subunit 6,Tat-binding protein 7,MB67 interacting protein	CATCGGACAATTTCTGGAGGCTGTGGATCAGAATACAGCCATCGTGGGCTCTACCACAGGCTCCAACTATTATGTGCGCATCCTGAGCACCATCGATCGG	proteasome 26S subunit, ATPase 4
PUM1	NM_0010	pumilio homolog 1 (Drosophila);PUMH1,KIAA0099	CTGGGGAACATCAGATCATTCAGTTTCCCAGCCAATCATGGTGCAGAGAAGACCTGGTCAGAGTTTCCATGT	pumilio RNA binding family member 1

	20658.1		GAACAGTGAGGTCAATTCTGTACTGTCC	
SDHA	NM_004168.3	SDH2;succinate dehydrogenase complex, subunit A, flavoprotein (Fp),succinate dehydrogenase complex subunit A, flavoprotein (Fp);FP,SDHF;succinate dehydrogenase [ubiquinone] flavoprotein subunit,flavoprotein subunit of complex II	AGGGGCAGGCTTGCGAGCTGCATTTGGCCTTTCTGAGGCAGGGTTTAATACAGCATGTGTTACCAAGCTGTTTCCTACCAGGTCACACACTGTTGCAGCA	succinate dehydrogenase complex flavoprotein subunit A
SF3A1	NM_005877.4	splicing factor 3a, subunit 1, 120kD,splicing factor 3a, subunit 1, 120kDa;SF3a120,SAP114,PRPF21,Prp21	GATGATGAGGTGTACGCACCAGGTCTGGATATTGAGAGCAGCTTGAAGCAGTTGGCTGAGCGGCGTACTGACATCTTCGGTGTAGAGGAAACAGCCATTG	splicing factor 3a subunit 1
STK11IP	NM_052902.2	LIP1,KIAA1898,LKB1IP,STK11IP1;LKB1 interacting protein	GCTCTGCTTTCTGCCAACTTCAGCTACAATGCACTGACCGCCTTAGACAGCTCCCTGCGCCTCTTGTCAGCTCTGCGTTTCTTGAACCTAAGCCACAATC	serine/threonine kinase 11 interacting protein
TBC1D10B	NM_015527.3	TBC1 domain family, member 10B;DKFZP434P1750,Rab27A-GAPbeta,FLJ13130,EPI64B	GCGTTGGCCTGGGGTGGGGGTGCTCGCTTTGTCTTCTGTCCTTTGGTTCTCCTTCCATAATGCTCCTGTACCCAGTTTATTTAAGGGGACATGCACTGGA	TBC1 domain family member 10B
TBP	NM_001172085.1	GTF2D1,SCA17;TFIID	ACAGTGAATCTTGGTTGTAAACTTGACCTAAAGACCATTGCACTTCGTGCCCGAAACGCCGAATATAATCCCAAGCGGTTTGCTGCGGTAATCATGAGGA	TATA-box binding protein
TFRC	NM_003234.1	transferrin receptor (p90, CD71);CD71,TFR1,p90	CAGTTTCCACCATCTCGGTCATCAGGATTGCCTAATATACCTGTCCAGACAATCTCCAGAGCTGCTGCAGAAAAGCTGTTTGGGAATATGGAAGGAGACT	transferrin receptor

TLK2	NM_006852.3	tousled-like kinase 2;PKU-ALPHA,MGC44450	TACGATTCTTAAAGCTACTGAAGTGCAGTTCCCGCCAAAGCCAGTAGTAACACCTGAAGCAAAGGCGTTTATTCGACGATGCTTGGCCTACCGAAAGGAG	tousled like kinase 2
TMUB2	NM_177441.2	transmembrane and ubiquitin-like domain containing 2;MGC3123	CTGTCTTTGTGGTGCTGTTGGGTGTGGTCTGGTACTTCCGAATCAATTACCGCCAATTCTTCACAGCACCTGCCACTGTCTCCCTGGTGGGAGTCACCGT	transmembrane and ubiquitin like domain containing 2
UBB	NM_018955.3	MGC8385,FLJ25987;polyubiquitin B	ACCTGGTCCTGCGCCTGAGGGGTGGCTGTTAATTCTTCAGTCATGGCATTCGCAGTGCCCAGTGATGGCATTACTCTGCACTATAGCCATTTGCCCCAAC	ubiquitin B
